# Borohydride Oxidation as Counter Reaction in Reductive Electrosynthesis

**DOI:** 10.1002/anie.202501653

**Published:** 2025-04-03

**Authors:** Julius Kuzmin, Malin Lill, Guillermo Ahumada, Ellymay Goossens, Astrid Kjær Steffensen, Anders Riisager, Helena Lundberg

**Affiliations:** ^1^ Department of Chemistry KTH Royal Institute of Technology 10044 Stockholm Sweden; ^2^ Department of Chemistry Technical University of Denmark Kgs. Lyngby Denmark

**Keywords:** Organic electrosynthesis, tetrabutylammonium borohydride, counter reaction, reductive transformations

## Abstract

An efficient reaction at the counter electrode is of key importance for the success of net oxidative and net reductive electrochemical transformations. For electrooxidative processes, cathodic proton reduction to H_2_ serves as the benchmark counter reaction. In contrast, net reductive electrochemical transformations have less attractive oxidative counter reactions to choose from and commonly rely on dissolution of a sacrificial anode that effectively results in stoichiometric metal consumption for the processes. In this study, we demonstrate that anodic borohydride oxidation has great potential to successfully replace the use of such sacrificial anodes for a variety of electroreductive organic transformations. This anodic transformation effectively serves as the inverse of cathodic proton reduction, producing H_2_ using inert carbon‐based electrode materials.

## Graphical abstract

Herein, anodic borohydride oxidation is demonstrated to have great potential for successfully replacing sacrificial metal anodes in a variety of electroreductive organic transformations. This anodic counter reaction effectively serves as the inverse of cathodic proton reduction, producing H_2_ at inert carbon‐based electrode materials.

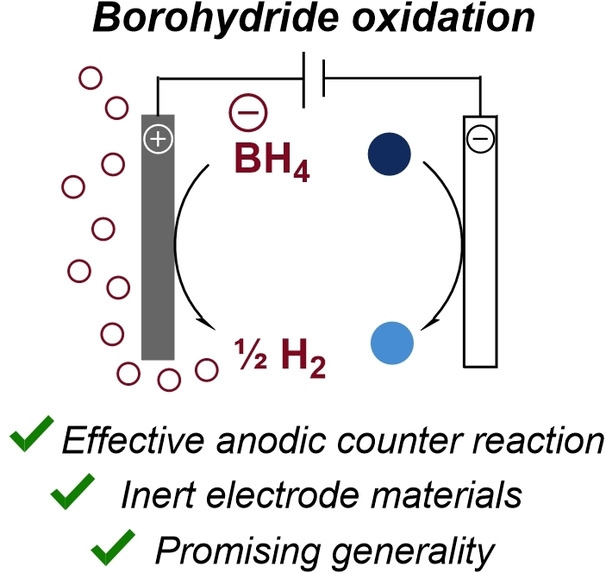

In recent years, electrosynthesis has emerged as a highly promising strategy for chemical synthesis.^[1]^ Due to the tunable nature, electrosynthesis can offer synthetic avenues with new reactivity and selectivity compared to traditional strategies. Furthermore, the use of (renewable) electricity to drive chemical transformations can result in sustainability benefits such as higher atom economy, less chemical waste, lower energy consumption and improved safety.^[2]^ Electrochemically driven transformations can proceed via electron transfer to or from an organic substrate at the electrode surface (direct electrolysis) or in solution via a redox‐active catalyst that is (re)generated at the electrode (indirect electrolysis).^[3]^ The electrode at which a targeted transformation occurs is denoted as the working electrode—e.g., the anode in an electrooxidative reaction—and the opposite electrode is called the counter electrode. To close the circuit in an electrochemical setting, the net number of electrons needs to be balanced between anode and cathode.

Various strategies have been developed to balance oxidative and reductive electroorganic transformations. In the ideal case, high‐value products are generated on both electrodes in a paired electrolysis to maximize the Faradaic efficiency. The simplest version of this strategy is parallel paired electrolysis of two different substrates to generate two distinct products (Figure 1, top left).[^4^, ^5^] Commonly, such parallel paired electrolytic processes are carried out in divided cells where the anodic and cathodic reaction chambers are separated from each other. While divided cells can allow good control over reaction pathways, their design may negatively impact user‐friendliness, result in challenging process scale‐ups and low overall energy‐efficiency due to the increased resistance that the physical barrier in the cell induces.[^5^, ^6^, ^7^, ^8^, ^9^] Provided that the organic products from the parallel paired electrolysis are easy to separate from each other, a more straight‐forward undivided electrolytic cell setup may be used. This strategy has, for example, been successfully implemented in an industrial setting, producing *p*‐tert‐butylbenzaldehyde dimethyl acetal and phthalide on multi‐ton scale.[^10^, ^11^] Following the same logic, cathodic hydrogen evolution (HER) via proton reduction is attractive to couple with anodic oxidation of organic compounds to generate H_2_ for fuel purposes at a lower cell potential compared to water electrolysis, along with the formation of valorized organic products.[^12^, ^13^, ^14^, ^15^, ^16^] Overall, cathodic proton reduction represents the benchmark counter reaction for net oxidative electroorganic transformations (Figure 1, top right). For net reductive transformations, however, the choices of anodic counter reactions are more diverse. In a laboratory setting, sacrificial anodes made from readily oxidized metals such as zinc (Zn), magnesium (Mg) or aluminum (Al) are commonly employed.[^17^, ^18^] This approach results in continuous release of metal ions from the dissolving anode (Figure 1, top right), thereby typically promoting high selectivity and yields as parasitic oxidative transformations of starting materials, intermediates and/or products are suppressed. Nevertheless, the stoichiometric use of metal is not ideal from a sustainability perspective and can result in undesired side‐reactions, such as Grignard reagent formation with aryl halides.[^19^, ^20^, ^21^, ^22^, ^23^, ^24^, ^25^, ^26^, ^27^, ^28^, ^29^] Furthermore, the anode erosion inevitably results in a continuous change in inter‐electrode distance and the formation of passivating layers of inorganic salts on the electrode is common, both of which negatively impact electrolysis outcome, not the least for large scale setups and flow processes.[^30^, ^31^] As an alternative strategy, oxidation of chemical species, such as solvent or stoichiometric reagents, can be used to balance a net cathodic transformation (Figure 1, top right).[^32^, ^33^, ^34^] For example, direct oxidation of amines,[^35^, ^36^, ^37^, ^38^, ^39^, ^40^, ^41^, ^42^] carboxylic acids,^[43]^ phosphines,[^31^, ^32^] thioureas,^[44]^ bromide ions,[^45^, ^46^] as well as the use of H_2_ as anodic fuel by means of quinone mediation in a divided cell,^[47]^ have been successfully employed. With each system having its own merits, the scarcity of unified approaches to replace sacrificial metal anodes as counter reaction for electroreductive transformations can result in excessive screening of reaction conditions. For this reason, the elegant system recently disclosed by Lennox and co‐workers was a welcome addition to the field.^[48]^ In this case, anodic bromide oxidation was coupled with silane trapping, providing an efficient alternative to the use of sacrificial anodes for pinacol couplings and disilylation of alkenes. In addition, the system enabled the transition from divided to more user‐friendly undivided cell setups for electroreductive hydrodefluorinations and ketone acylation.

**Figure 1:**
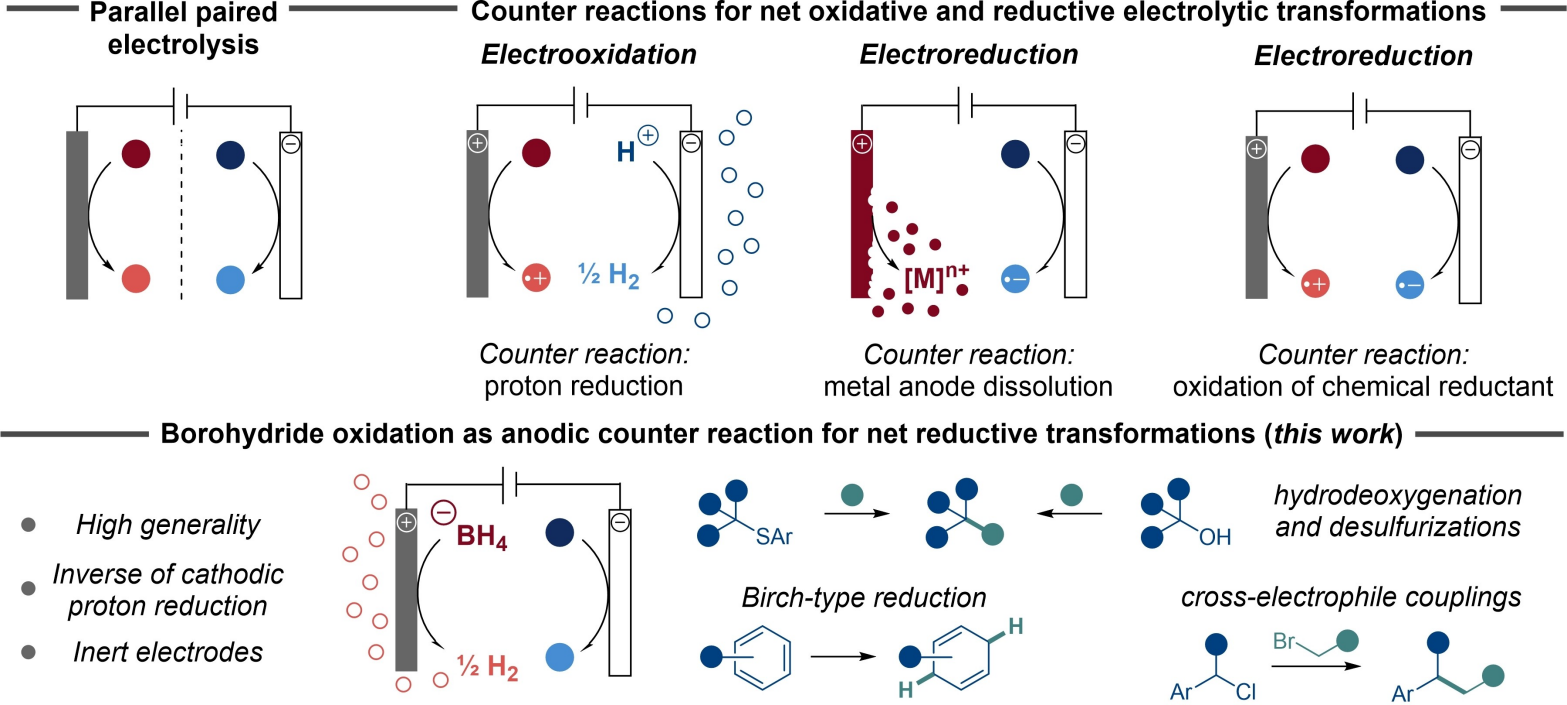
Top left: Parallel paired electrolysis. Top right: Various counter reactions in electrosynthesis. Bottom: Borohydride oxidation as anodic counter reaction (this work).

Borohydride (BH_4_) reagents remains severely underdeveloped in electrosynthetic transformations,[^49^, ^50^] despite their use as terminal reductant in fuel cell applications.[^33^, ^34^, ^51^, ^52^, ^53^] We recently demonstrated that borohydride oxidation can serve as an efficient anodic counter reaction in electroreductive synthesis, effectively serving as the inverse of cathodic proton reduction that commonly balance electrooxidative transformations.^[54]^ Here, we show that such anodic borohydride oxidation has the potential to serve as a general, effective and user‐friendly counter reaction to electroreductive transformations that can replace the use of sacrificial metal anodes (Figure 1, bottom).

Five net reductive organic transformations were chosen to assess the interchangeability between the use of a sacrificial metal anode and the use of an inert carbon‐based anode with tetrabutylammonium borohydride (NBu_4_BH_4_) as anodic fuel: hydrodesulfurization of thioethers, hydrodeoxygenation of alcohols, Birch‐type reduction of arenes, cross‐electrophile coupling of alkyl halides and desulfurative borylation. The first transformation—hydrodesulfurization of aryl alkyl thioethers—was recently reported by us.^[55]^ Gratifyingly, the transformation could successfully be carried out using either a sacrificial magnesium anode (conditions A) or a glassy carbon (GC) anode with NBu_4_BH_4_ as electrolyte (conditions B) in acetonitrile (MeCN) with a tin (Sn) cathode, producing near identical yields of alkanes **2 a**–**e** using either counter reaction strategy (Figure 2, top left). Control reactions using conditions B in the absence of either NBu_4_BH_4_ or current did not result in any product formation, confirming that the transformation is electrochemically driven, and that the borohydride is required for electroreductive product formation.

**Figure 2:**
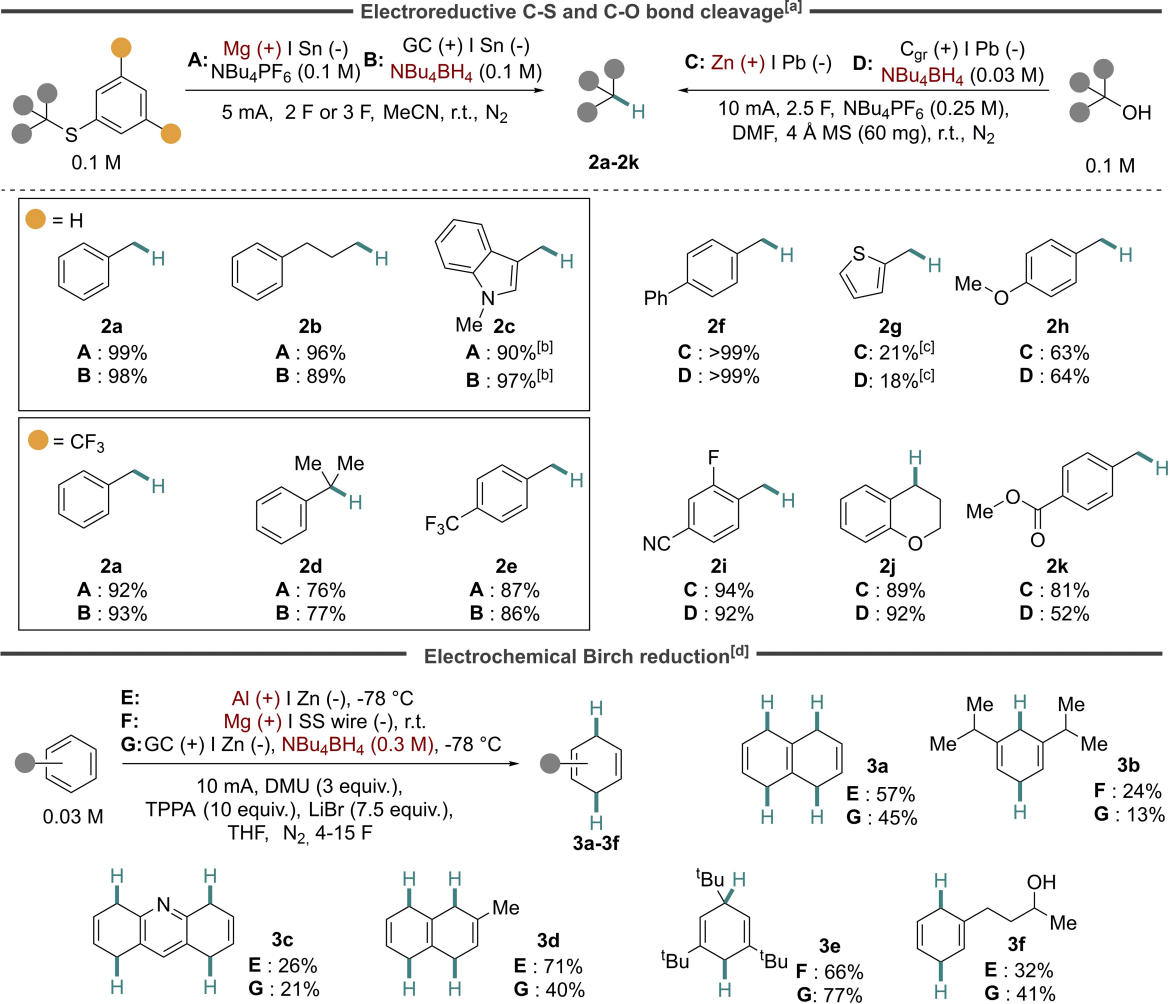
Top: Electrochemical C−S and C−O bond cleavage. Bottom: Electrochemical Birch reduction. [a] Yield determined by HPLC. [b] Yield determined by GC‐FID. [c] Isolated yield. [d] Yield determined by ^1^H NMR using 1,3,5‐trimethoxybenzene as internal standard.

Similar to the hydrodesulfurization protocol, hydrodeoxygenation of benzylic alcohols was also recently demonstrated by us.^[54]^ Comparable yields were obtained for a variety of substrates also in this case, using either a Zn anode (conditions C) or a graphite (C_gr_) anode with a substoichiometric amount of NBu_4_BH_4_ (conditions D) in a *N,N*‐dimethylformamide (DMF) electrolyte, resulting in alkanes **2 f**–**k** (Figure 2, top right). Here, the functional group tolerance encompassed moieties such as ethers, nitriles and aryl fluorides, whereas the ester‐substituted **2 k** resulted in a lower yield using the NBu_4_BH_4_ strategy. A control experiment for conditions D in the absence of current failed to furnish alkane **2 f**, whereas the absence of NBu_4_BH_4_ resulted in a significantly lower yield of **2 f** compared to benchmark conditions D (32 % versus >99 %) along with oxidation products of the parent alcohol.

We continued our assessment of borohydride oxidation as anodic counter reaction for electrochemical Birch‐type reduction, developed by Baran and co‐workers.^[56]^ In this case, the reported conditions that included tris(pyrrolidine)phosphine oxide (TPPA), dimethyl urea (DMU) and lithium bromide (LiBr) in tetrahydrofuran (THF) at either −78 °C with an aluminum anode and a zinc cathode (conditions E) or at 25 °C with a magnesium anode and a stainless steel (SS) wire as cathode (conditions F) were used as starting points. As the alternative set of conditions, the Al or Mg anode was replaced by a glassy carbon electrode and NBu_4_BH_4_ (1 equiv.) (conditions G). As shown in Figure 2, the reduced Birch‐type products were formed in all cases using either set of conditions. Products **3 a**–**3 c** formed in similar yields using the two protocols, while substrate **3 d** resulted in a lower yield using conditions G. In contrast, compound **3 e** and **3 f** formed in higher yields under conditions G compared to conditions F. Notably, these results were obtained without further optimization of conditions as the switch from a sacrificial anode (conditions E and F) to a glassy carbon anode and borohydride (conditions G) was made. As such, these results clearly demonstrate the potential of the borohydride oxidation strategy in terms of general applicability if applied in the early stages of method development. Control experiments for conditions G revealed that compound **3 a** did not form in the absence of current, and only in 17 % without NBu_4_BH_4_, clearly demonstrating that both electricity and borohydride reagent are required for a successful outcome.

Next, cross‐electrophile C(sp^3^)‐C(sp^3^) coupling (XEC) of alkyl halides was assessed using the protocol developed by Lin and co‐workers, using a sacrificial magnesium anode under the published benchmark conditions (conditions H).^[57]^ For comparison, the anodic counter reaction was switched from anode dissolution to borohydride oxidation at a glassy carbon electrode (conditions I). As evident from Figure 3, these conditions were able to deliver the targeted products **4 a**–**c**, although in lower yields compared to conditions H. Again, no optimization of reaction parameters such as solvent or supporting electrolyte was carried out. Considering that borohydride reagents are known to induce hydrodehalogenation of alkyl halides under certain conditions,[^58^, ^59^, ^60^, ^61^] adjustments of experimental parameters may result in higher yields of the targeted XEC products.^[62]^ Control experiments using conditions I in the absence of either NBu_4_BH_4_ or current failed to furnish product **4 a**.

**Figure 3:**
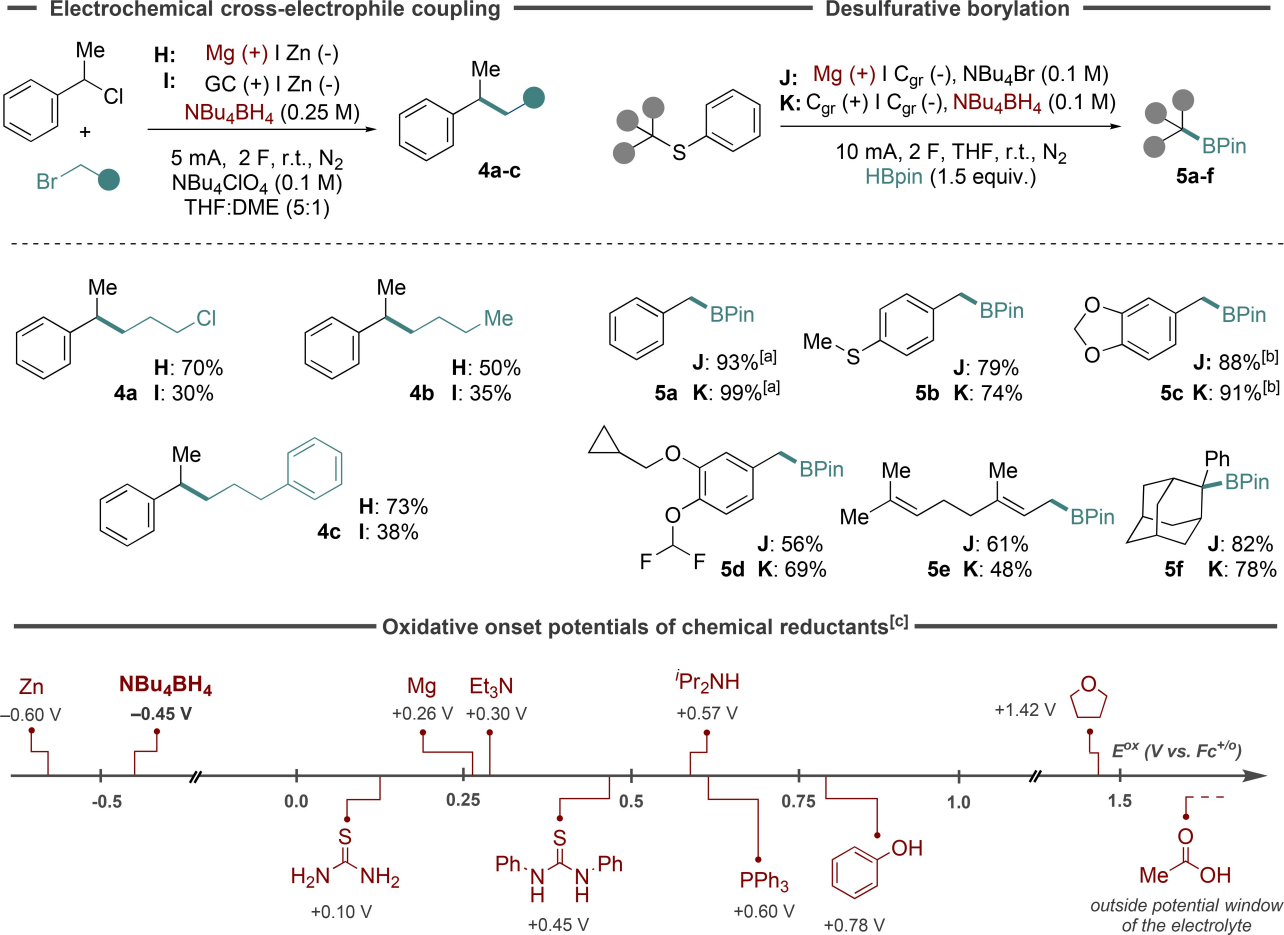
Top left: Cross‐electrophile coupling (XEC) of alkyl halides. Top right: Desulfurative borylation of thioethers. Bottom: Oxidative onset potentials of chemical reductant and sacrificial anodes. [a] Isolated yield. [b] 3 equiv. HBpin. [c] 4 F. [d] Determined by cyclic voltammetry (see section S3 in Supporting Information).

Lastly, desulfurative borylation to furnish alkylboronic esters was assessed using either a sacrificial magnesium anode (conditions J) or a graphite anode in presence of NBu_4_BH_4_ (conditions K).^[63]^ For this cross‐coupling protocol, good to excellent yields of the corresponding borylated products from a selection of benzylic and allylic phenyl thioethers were obtained using either set of conditions (Figure 3, top right), clearly demonstrating the complementary nature of the two anodic counter reaction strategies. Control experiments for conditions K revealed that compound **5 a** did not form in the absence of current and in mere 9 % with NBu_4_Br in absence of NBu_4_BH_4_, confirming that both electricity and borohydride reagents are required for successful reaction outcome.

To probe the reactivity of borohydride reagents relative to that of other chemical reductants, a selection of compounds was assessed with cyclic voltammetry (CV). As shown in Figure 3, the sacrificial anode material Zn displayed the lowest oxidative onset potentials of around −0.6 V vs Fc^+/0^. Closely following this value, the oxidative onset potential of NBu_4_BH_4_ was determined to −0.45 V vs Fc^+/0^, whereas the sacrificial anode material Mg and all organic reductants were oxidized at considerably more positive potentials. These measurements were carried out in a THF electrolyte (0.1 M NBu_4_PF_6_) (see Supporting Information, section S3) and it should be noted that redox potentials can be highly condition dependent.^[64]^ Nevertheless, the trend from these measurements demonstrate the potential of borohydrides as anodic fuel to replace sacrificial metal anodes in net reductive electrochemical transformations. Provided that chemical compatibility of starting materials and borohydride reagents is at hand, the low oxidative onset potential of the latter suggests that they would be more efficient in suppressing unwanted anodic transformations compared to other common chemical reductants such as amines, thioureas and phosphines.

In summary, we showcase tetrabutylammonium borohydride as a competent fuel for the anodic counter electrode process for various net reductive electroorganic transformations. We demonstrate that this reagent, in combination with an inert carbon‐based anode, may successfully replace sacrificial metal anodes to furnish the desired products. While the approach resulted in lower product yields for electroreductive cross‐electrophile coupling of alkyl halides compared to the use of a sacrificial Mg anode, electrochemical Birch‐type reduction displayed a high degree of substrate dependence as to which anodic counter reaction that was optimal. Notably, no optimization was carried out in these cases and improved yields may thus result upon tuning of conditions. For electroreductive hydrodeoxygenation of benzylic alcohols and desulfurative transformations of thioethers, the desired products were formed in comparable yields using either counter reaction strategy. As such, the results from this study demonstrate the promising generality of anodic borohydride oxidation as counter reaction to electroreductive transformations. Due to the user‐friendly setup and high efficiency, this inverse of proton reduction—the benchmark counter reaction for electrooxidative transformations—shows potential for serving as a future go‐to counter reaction in an electroreductive manifold, provided that chemical compatibility of reagents and reactants with the anode fuel is at hand.

## Associated Content

Supporting Information. This material is available free of charge via the Internet.

## Funding Sources

This work was financially supported by the Swedish Research Council (grant no. 2021‐05551), the European Research Council (grant no. 101164660), the Swedish Foundation for Strategic Research (grant no. FFL21‐0005), Stiftelsen Olle Engkvist Byggmästare, Magnus Bergvalls stiftelse, Stiftelsen Lars Hiertas Minne, KTH Royal Institute of Technology and the Technical University of Denmark.

## Author Contributions

The manuscript was written through contributions of all authors. All authors have given approval to the final version of the manuscript.

## Conflict of Interests

The authors declare no conflict of interest.

## Supporting information

As a service to our authors and readers, this journal provides supporting information supplied by the authors. Such materials are peer reviewed and may be re‐organized for online delivery, but are not copy‐edited or typeset. Technical support issues arising from supporting information (other than missing files) should be addressed to the authors.

Supporting Information

## Data Availability

The data that support the findings of this study are openly available in ChemRxiv at https://chemrxiv.org/engage/chemrxiv/article‐details/676191f3fa469535b9e78189, reference number 1.
