# Supplementary material for: Borohydride Oxidation as Counter Reaction in Reductive Electrosynthesis
Source: Angew Chem Int Ed Engl. 2025 Apr 3;64(20):e202501653. doi: 10.1002/anie.202501653 (PMC12070360; doi:10.1002/anie.202501653)

Angewandte Chemie
Eine Zeitschrift der Gesellschaft Deutscher Chemiker

Supporting Information

Borohydride Oxidation as Counter Reaction in Reductive Electrosynthesis

*J. Kuzmin, M. Lill, G. Ahumada, E. Goossens, A. Kjær Steffensen, A. Riisager, H. Lundberg**

Supporting Information

Borohydride Oxidation as Counter Reaction in Reductive Electrosynthesis

Julius Kuzmin,[‡a] Malin Lill,[‡a] Guillermo Ahumada,[a] Ellymay Goossens,[a] Astrid Kjær Steffensen,[a,b] Anders Riisager,[b] Helena Lundberg[*a]

[†] Department of Chemistry, KTH Royal Institute of Technology, SE-10044, Stockholm (Sweden).

[b] Department of Chemistry, Technical University of Denmark, Kgs. Lyngby, Denmark

[‡] Indicates equal contribution

KEYWORDS. Organic electrosynthesis, tetrabutylammonium borohydride, counter reaction, reductive transformations

Table of contents

S1 General Information 3

S2 Synthesis and characterization of substrates 4

S2.1 ... Comparison of reaction conditions 8

S2.1.1 ... Hydrodesulfurization of thioethers 8

S2.1.2 ... Hydrodeoxygenation of alcohols 8

S2.1.3 ... Electrochemical Birch reduction 9

S2.1.4 ... Cross-electrophile coupling of alkyl halides 9

S2.1.5 ... Desulfurative borylation of thioethers 10

S2.2 ... Synthetic details and data for products 11

S2.2.1 ... Products of hydrodesulfurization of thioethers 11

S2.2.2 ... Products of hydrodeoxygenation of alcohols 12

S2.2.3 ... Products of electrochemical Birch reduction 14

S2.2.4 ... Products of cross-electrophile coupling of alkyl halides 41

S2.2.5 ... Products of desulfurative borylation of thioethers 42

S3 Electrochemical measurements 44

S4 NMR data 72

S4.1 ... Hydrodesulfurization of thioethers ^{1}H- and ^{13}C-NMR spectrum 72

S4.2 ... Cross-electrophile coupling ^{1}H- and ^{13}C-NMR spectrum 74

S4.3 ... 4a 74

S4.4 ... 4b 76

S4.5 ... 4c 78

S4.6 ... Borylation ^{1}H-, ^{13}C, and ^{11}B-NMR spectrum 80

S4.7 ... 5b 80

S4.8 ... 5c 83

S4.9 ... 5d 86

S4.10 . 5e 90

S4.11 . 5f 93

S1 General Information

All reactions were carried out in glassware that was not pre-dried unless otherwise stated. THF or DMF was dried using a solvent dispensing system, where solvent is passed through activated alumina columns, stored under N_2 and over activated 4 Å molecular sieves when needed. Water used for reactions and HPLC analysis was obtained from Milli-Q® system. Trifluoromethanesulfonic acid (triflic acid, TfOH) was stored and handled under N_2 atmosphere, using Hamilton syringes. Molecular sieves (4 Å, powder) were heat gun-dried under vacuum for 20 minutes and cooled under N_2 atmosphere before use. All other solvents and reagents were purchased from commercial suppliers and used without further purification unless otherwise noted. Thioethers used in protocols were either bought commercially or synthesized according to literature procedures.[1] In the preparation of the supporting electrolyte Bu_4NPF_6, it was first recrystallized from an ethanol/water (EtOH/H_2O, 3:1 v/v) mixture. This step was followed by three additional recrystallizations using ethanol. The product was then dried at 120 °C for 48 h under vacuum, achieving an anhydrous state. TLC analyses were performed on pre-coated silica gel 60 F254 plates and visualized using UV light, $KMnO_4$ (solution in a mixture of $KMnO_4$/K_2CO_3/NaOH in H_2O), phosphomolybdic acid stain (solution in EtOH), vanillin (solution in 1% H_2SO_4 in EtOH) or hydrazine (solution in EtOH). Flash column chromatography was conducted using 40-60 µm, 230-400 mesh, 60 Å silica gel as stationary phase. NMR spectra were recorded using either a Bruker Avance II 400 MHz or a Bruker Avance 500 MHz spectrometer at 298 K (unless otherwise stated) using $CDCl_3$, acetone-d_6, CD_3OD, D_2O, or toluene-d_8 as solvents. Chemical shifts are given in ppm relative to the residual solvent peak (^1H NMR: $CDCl_3$ δ 7.26; acetone-d_6 δ 2.05; toluene-d_8 δ 2.09; ^{13}C NMR: $CDCl_3$ δ 77.16; acetone-d_6 δ 29.84; toluene-d_8 δ 20.40) with multiplicity (br = broad, s = singlet, d = doublet, t = triplet, q = quartet, quin = quintuplet, sext = sextet, m = multiplet), coupling constants (in Hz) and integration. HPLC analysis was done with an Agilent 1260 Infinity Quaternary LC (Eclipse Plus 18C column) with a gradient of acetonitrile and 0.1% formic acid in Milli-Q water at a flow rate of 1.0 mL/min, using 4,4'-di-tert-butylbiphenyl (DTBB) as internal standard. High-resolution mass spectrometry analyses were performed using a Thermo Scientific Q Exactive HF Hybrid Quadrupole-Orbitrap HESI. Full analytical data is given if the compound is novel. Commercial IKA® ElectraSyn vials and caps were used for electrochemical experiments using the Electrasyn 2.0 or the Aim TTi MX100QP as the power supply. GC analysis was done by an Agilent Technologies 6850 instrument with FID using a chiral column Cyclosil-B (30 m x 0.25 mm x 0.25 um) from Agilent Technologies Inc.

S2 Synthesis and characterization of substrates

Hydrodesulfurization of thioethers

Method A: To an oven-dried 10 mL IKA® ElectraSyn vial equipped with a magnetic stir bar the corresponding starting material (1.0 equiv., 0.50 mmol) and NBu_4PF_6 (1.0 equiv., 0.5 mmol, 194 mg) were added. The vial was sealed with an IKA ElectraSyn vial cap equipped with a tin cathode and magnesium anode. The vial was evacuated and back flushed with nitrogen three times before adding anhydrous MeCN (5 mL). The reaction was carried out by applying 5 mA at room temperature for 3 F. After electrolysis, the solvent was evaporated under vacuum, and the resulting product was purified by column chromatography.

Method B: To an oven-dried 10 mL IKA® ElectraSyn vial equipped with a magnetic stir bar the corresponding starting material (1.0 equiv., 0.50 mmol) and NBu_4PF_6 (1.0 equiv., 0.5 mmol, 194 mg) and NBu_4BH_4 (1.0 equiv., 0.5 mmol, 131 mg) were added. The vial was sealed with an IKA ElectraSyn vial cap equipped with a tin cathode and glassy carbon anode. The vial was evacuated and back flushed with nitrogen three times before adding anhydrous MeCN (5 mL). The reaction was carried out by applying 5 mA at room temperature for 3 F. After electrolysis, the solvent was evaporated under vacuum, and the resulting product was purified by column chromatography.

Hydrodeoxygenation of alcohols

Method C: To an oven-dried 5 mL IKA® ElectraSyn vial equipped with a magnetic stir bar the alcohol (1 equiv., 0.3 mmol), NBu_4PF_6 (2.5 equiv., 0.75 mmol, 290.6 mg), and activated powdered 4 Å molecular sieves (60 mg) were added. To ensure airtight sealing, the screw joints of the vial were wrapped with PTFE tape, and the IKA® ElectraSyn cap, equipped with a zinc anode and a lead cathode were securely fastened. Before the reaction, the sealed vial was subjected to nitrogen gas flushing for 10 minutes. Anhydrous DMF (3.0 mL) was then added to the reaction vial. The reaction parameters were set on the IKA® ElectraSyn 2.0 interface, selecting 'New Experiments' and 'Constant Current' modes. The desired current was set to 10.0 mA, with no reference electrode used. The reaction time was fixed at 2.0 h, and the charge was adjusted to correspond to 0.30 mmol of the substrate. The option to alternate polarity was set to 'No'. Throughout the reaction, a continuous flow of nitrogen gas was maintained. The reaction system was connected to an N_2 Schlenk line with an inlet and outlet, ensuring an inert environment during the electrochemical process.

Method D: To an oven-dried 5 mL IKA® ElectraSyn vial equipped with a magnetic stir bar the alcohol (1 equiv., 0.3 mmol), NBu_4PF_6 (2.5 equiv., 0.75 mmol, 290.6 mg), NBu_4BH_4 (0.3 equiv., 0.09 mmol, 23.2 mg) and activated powdered 4 Å molecular sieves (60 mg) were added. To ensure airtight sealing, the screw joints of the vial were wrapped with PTFE tape, and the IKA® ElectraSyn cap, equipped with a graphite anode and a lead cathode were securely fastened. Before the reaction, the sealed vial was subjected to nitrogen gas flushing for 10 minutes. Anhydrous DMF (3.0 mL) was then added to the reaction vial. The reaction parameters were set on the IKA® ElectraSyn 2.0 interface, selecting 'New Experiments' and 'Constant Current' modes. The desired current was set to 10.0 mA, with no reference electrode used. The reaction time was fixed at 2.0 h, and the charge was adjusted to correspond to 0.30 mmol of the substrate. The option to alternate polarity was set to 'No'. Throughout the reaction, a continuous flow of nitrogen gas was maintained. The reaction system was connected to an N_2 Schlenk line with an inlet and outlet, ensuring an inert environment during the electrochemical process.

Electrochemical Birch reduction

DMU and LiBr preparation:[2] 1.5 g of LiBr (solid) was dried by heating it at 110 °C for 2 hours under a high vacuum in an oven-dried round-bottom flask. After that, the solid was sealed and kept under nitrogen. A LiBr stock solution (1.5 M, THF) was used for five days. Azeotropic drying was used to dry DMU. In an oven-dried 100 ml round-bottom flask, DMU (5 g) was dissolved in 5.0 mL methanol, and 5.0 ml toluene was added and then removed by rotary evaporation. The solid was then dissolved in 5 mL of toluene and removed using rotary evaporation. This procedure was repeated three times, and the dried solid was stored in a sealed vial.

Method E: To an oven-dried 5.0 mL IKA® ElectraSyn vial with a magnetic stir bar the substrate (1 equiv., 0.1 mmol), N,N'-dimethylurea (DMU, 3 equiv., 0.3 mmol, 26.4 mg) and tri(pyrrolidin-1-yl)phosphine oxide (10 equiv., 1.0 mmol, 230 µL) was added. The vial joints were wrapped with PTFE tape and further sealed with an IKA® ElectraSyn cap equipped with aluminium anode and zinc cathode. The vial was flushed with nitrogen gas for 10 minutes, then 0.5 mL of a 1.5 M LiBr solution was added. Lastly, dry THF (3.0 mL) was added. The stirring speed was set to 700 rpm. The reaction was run at -78 °C. The reaction parameters were set on the IKA® ElectraSyn 2.0 interface, selecting 'New Experiments' and 'Constant Current' modes. The desired current was set to 10.0 mA, with no reference electrode used. The substrate loading was set to 0.1 mmol and the charge was fixed and varied depending on the starting material (see S2.1 Synthetic details and data for products). The option to alternate polarity was set to 'No'. Throughout the reaction, a continuous flow of nitrogen gas was maintained. The reaction system was connected to an N_2 Schlenk line, ensuring an inert environment during the electrochemical process. When the reaction was finished, the volatiles were removed under reduced pressure using a rotatory evaporator. The crude was dissolved in 6 mL of a pentane/ethyl acetate (3:1) mixture and then passed through a silica gel plug (Note: ^{1}H-NMR analyzed supernatants, and no starting material or product signal was detected). The volatiles were carefully removed under reduced pressure before adding 1,3,5-trimethoxybenzene (5-10 mg). Finally, the crude reaction mixture was dissolved $CDCl_3$, and the conversion and yield were determined by ^{1}H-qNMR analysis of integral values.

Method F: To an oven-dried 5.0 mL IKA® ElectraSyn vial with a magnetic stir bar the substrate (1 equiv., 0.1 mmol), N,N'-dimethylurea (DMU, 3 equiv., 0.3 mmol, 26.4 mg) and tri(pyrrolidin-1-yl)phosphine oxide (10 equiv., 1.0 mmol, 230 µL) was added. The vial joints were wrapped with PTFE tape and further sealed with an IKA® ElectraSyn cap equipped with magnesium anode and stainless-steel wire cathode. The vial was flushed with nitrogen gas for 10 minutes, then 0.5 mL of a 1.5 M LiBr solution was added. Lastly, dry THF (3.0 mL) was added. The stirring speed was set to 700 rpm. The reaction was run at 25 °C. The reaction parameters were set on the IKA® ElectraSyn 2.0 interface, selecting 'New Experiments' and 'Constant Current' modes. The desired current was set to 10 mA, with no reference electrode used. The substrate loading was set to 0.1 mmol and the charge was fixed and varied depending on the starting material (see S2.1 Synthetic details and data for products). The option to alternate polarity was set to 'No'. Throughout the reaction, a continuous flow of nitrogen gas was maintained. The reaction system was connected to an N_2 Schlenk line, ensuring an inert environment during the electrochemical process. When the reaction was finished, the volatiles were removed under reduced pressure using a rotatory evaporator. The crude was dissolved in 6 mL of a pentane/ethyl acetate (3:1) mixture and then passed through a silica gel plug (Note: ^{1}H-NMR analyzed supernatants, and no starting material or product signal was detected). The volatiles were carefully removed under reduced pressure before adding 1,3,5-trimethoxybenzene (5-10 mg). Finally, the crude reaction mixture was dissolved $CDCl_3$, and the conversion and yield were determined by ^{1}H-qNMR analysis of integral values.

Method G: To an oven-dried 5.0 mL IKA® ElectraSyn vial with a magnetic stir bar the substrate (1 equiv., 0.1 mmol), N,N'-dimethylurea (DMU, 3 equiv., 0.3 mmol, 26.4 mg), NBu_4BH_4 (1 equiv., 0.1 mmol, 25.7 mg) and tri(pyrrolidin-1-yl)phosphine oxide (10 equiv., 1.0 mmol, 230 µL) was added. The vial joints were wrapped with PTFE tape and further sealed with an IKA® ElectraSyn cap equipped with a glassy carbon anode and zinc cathode. The vial was flushed with nitrogen gas for 10 minutes, then 0.5 mL of a 1.5 M LiBr solution was added. Lastly, dry THF (3.0 mL) was added. The stirring speed was set to 700 rpm. The reaction was run at -78 °C. The reaction parameters were set on the IKA® ElectraSyn 2.0 interface, selecting 'New Experiments' and 'Constant Current' modes. The desired current was set to 10 mA, with no reference electrode used. The substrate loading was set to 0.1 mmol and the charge was fixed and varied depending on the starting material (see S2.1 Synthetic details and data for products). The option to alternate polarity was set to 'No'. Throughout the reaction, a continuous flow of nitrogen gas was maintained. The reaction system was connected to an N_2 Schlenk line, ensuring an inert environment during the electrochemical process. When the reaction was finished, the volatiles were removed under reduced pressure using a rotatory evaporator. The crude was dissolved in 6 mL of a pentane/ethyl acetate (3:1) mixture and then passed through a silica gel plug (Note: ^{1}H-NMR analyzed supernatants, and no starting material or product signal was detected). The volatiles were carefully removed under reduced pressure before adding 1,3,5-trimethoxybenzene (5-10 mg.). Finally, the crude reaction mixture was dissolved $CDCl_3$, and the conversion and yield were determined by ^{1}H-qNMR analysis of integral values.

Cross-electrophile coupling of alkyl halides

Method H: To an oven-dried 10 ml IKA® ElectraSyn vial with a magnetic stir bar, NBu_4ClO_4 (2.0 equiv., 2.0 mmol, 0.68 g,) was weighed in. Subsequently, the vial was sealed with an IKA® ElectraSyn cap equipped with an magnesium anode and a zinc cathode and flushed with nitrogen gas. 1,2-dimethoxyethane (DME, 1.0 mL) and anhydrous THF (8.0 mL) was added and the mixture was stirred for 2.0 min with a rate of 700 rpm, ensuring complete dissolution of the electrolyte. After stirring the mixture, it was submitted to constant current electrolysis of 5 mA at room temperature for 2 F. After 1 min of electrolysis, the alkyl chloride (1 equiv., 2.0 mmol) and the alkyl bromide (2 equiv., 4.0 mmol) were sequentially added to the mixture. After electrolysis the crude reaction mixture was extracted with diethyl ether and washed with brine. The organic phase was dried over sodium sulfate and purified by column chromatography on silica gel.

Method I: To an oven-dried 10 ml IKA® ElectraSyn vial with a magnetic stir bar, NBu_4ClO_4 (2.0 equiv., 2.0 mmol, 0.68 g) and NBu_4BH_4 (1 equiv., 2 mmol, 415.6 mg) was weighed in. Subsequently, the vial was sealed with an IKA® ElectraSyn cap equipped with a glassy carbon anode and a zinc cathode and flushed with nitrogen gas. 1,2-dimethoxyethane (DME, 1.0 mL) and anhydrous THF (8.0 mL) was added and the mixture was stirred for 2.0 min with a rate of 700 rpm, ensuring complete dissolution of the electrolyte. After stirring the mixture, it was submitted to constant current electrolysis of 5 mA at room temperature for 2 F. After 1 min of electrolysis, the alkyl chloride (1 equiv., 2.0 mmol) and the alkyl bromide (2 equiv., 4.0 mmol) were sequentially added to the mixture. After electrolysis the crude reaction mixture was extracted with diethyl ether and washed with brine. The organic phase was dried over sodium sulfate and purified by column chromatography on silica gel.

Desulfurative borylation of thioethers

Method J: To an oven-dried 10 mL IKA® ElectraSyn vial equipped with a magnetic stir bar, the corresponding starting material (1.0 equiv., 0.50 mmol) and NBu_4Br (1.0 equiv., 0.50 mmol, 161 mg) were weighed in. The vial was sealed with an IKA® ElectraSyn vial cap equipped graphite cathode and magnesium anode. The vial was evacuated and back flushed with nitrogen three times before adding anhydrous stabilizer-free THF (5 mL) followed by HBPin (1.5 equiv., 0.75 mmol, 110 µL). The reaction was carried out by applying 10 mA (~10 mA/cm^2) at room temperature for 2 F under stirring (750 rpm). After electrolysis, the solvent was evaporated under vacuum and the crude reaction mixture was dissolved in EtOAc and extracted with an aqueous solution of NH_4Cl (30 mL) and EtOAc (15 mL x3). The combined organic phases were dried over sodium sulfate and purified by column chromatography on oven-dried silica gel.

Method K: To an oven-dried 10 mL IKA® ElectraSyn vial equipped with a magnetic stir bar, the corresponding starting material (1.0 equiv., 0.50 mmol), NBu_4Br (1.0 equiv., 0.50 mmol, 161 mg) and NBu_4BH_4 (1.0 equiv., 0.50 mmol, 130 mg) were weighed in. The vial was sealed with an IKA® ElectraSyn vial cap equipped with graphite cathode and graphite anode. The vial was evacuated and back flushed with nitrogen three times before adding anhydrous stabilizer-free THF (5 mL) followed by HBPin (1.5 equiv., 0.75 mmol, 110 µL). The reaction was carried out by applying 10 mA (~10 mA/cm^2) at room temperature for 2 F under stirring (750 rpm). After electrolysis, the solvent was evaporated under vacuum and the crude reaction mixture was dissolved in EtOAc and extracted with an aqueous solution of NH_4Cl (30 mL) and EtOAc (15 mL x3). The combined organic phases were dried over sodium sulfate and purified by column chromatography on oven-dried silica gel

S2.1 Comparison of reaction conditions

S2.1.1 Hydrodesulfurization of thioethers

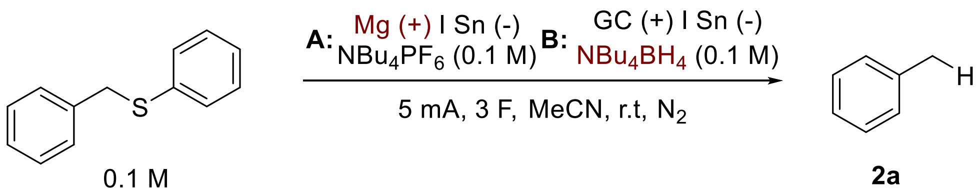

Table S1. Hydrodesulfurization of benzylphenyl thioether under different conditions.

**Entry**	**Method**	**Deviation from above**	**Yield 2a [%]**[a]
**1**	A	-	99
**2**	B	-	98
**3**	B	NBu_4PF_6 (0.1 M)	0
**4**	B	No applied current	0

[a]Determined through HPLC analysis

S2.1.2 Hydrodeoxygenation of alcohols

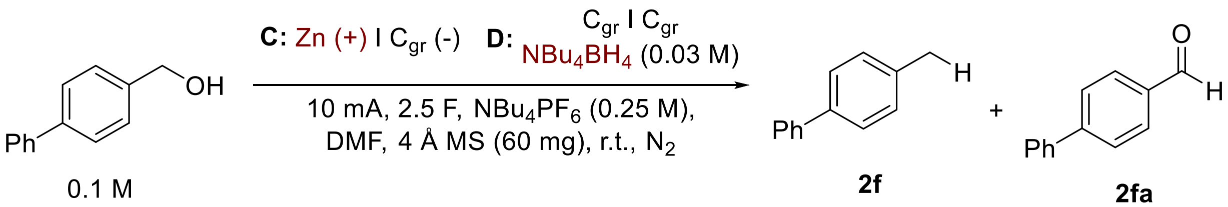

Table S2. Hydrodeoxygenation of 4-biphenylmethanol under different conditions.

**Entry**	**Method**	**Deviation from above**	**Yield 2f [%]**[a]	**Yield 2fa [%]**[a]	**Conversion [%]**[a]
**1**	C; D	none	<99; <99	n.d.	<99; <99
**2**	D	No NBu_4BH_4	32	36	<99
**3**	C	No applied current	0	0	0
**4**	D	No applied current	0	0	0

[a]Determined through HPLC analysis
n.d. – Not determined

S2.1.3 Electrochemical Birch reduction

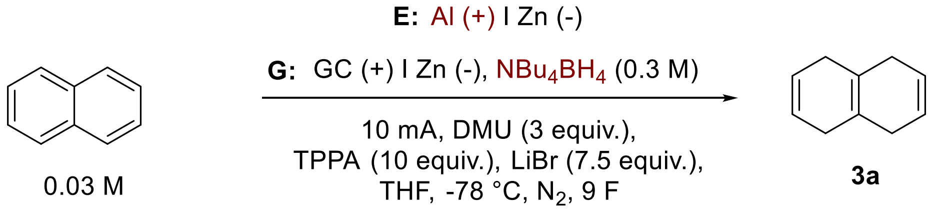

Table S3. Electrochemical Birch reduction of napththalene under different conditions.

Entry	Method	Deviation from above	Yield 3a [%][a]	Conversion [%][a]
**1**	G	None	46	97
**2**	G	0.5 equiv. NBu_4BH_4	38	97
**3**	G	No NBu_4BH_4	17	98
**4**	G	4.5 F	32	75
**5**	G	C_{Gr} as anode	44	97
**6**	G	BDD as anode	20	98
**7**	G	Room temp.	18	82
**8**	G	No applied current	n.d.	0

[a]Determined through qNMR analysis using 1,3,5-trimethoxybenzene as internal standard (see S1. Synthesis and characterisation of product – Method E-G)

S2.1.4 Cross-electrophile coupling of alkyl halides

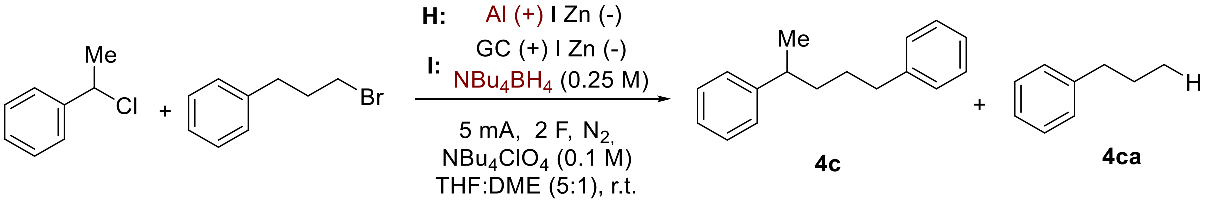

Table S4. Cross-electrophile coupling of 1-(chloroehtly)benzene and 3-(bromopropyl)benzene under different conditions.

Entry	Method	Deviation from above	Yield 4c [%]	Yield 4ca [%]	Conversion R^1-Cl [%][b]	Conversion R^2-Br [%][b]
**1**	I	None	38[a]	n.d	n.d.	n.d.
**2**	I	No Bu_4NBH_4	3[b]	n.d.	2	2
**3**	I	No current	n.d.	60[b]	0	56

[a]Isolated yield
[b]Determined through HPLC analysis
n.d. – Not determined

S2.1.5 Desulfurative borylation of thioethers

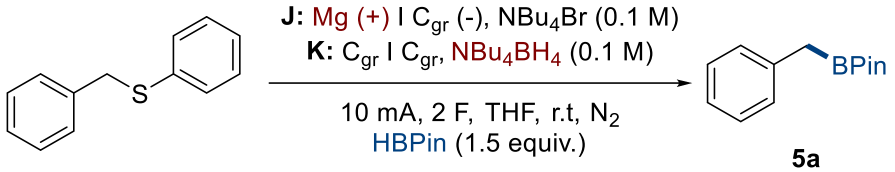

5a

Table S5. Desulfurative borylation of benzylphenyl thioether under different conditions**.**

Entry	Method	Deviation from above	Yield 5a [%][a]
**1**	J	-	93
**2**	K	-	99
**3**	K	NBu_4Br (0.1 M)	9
**4**	K	No applied current	0

[a]Determined through HPLC analysis

S2.2 Synthetic details and data for products

S2.2.1 Products of hydrodesulfurization of thioethers

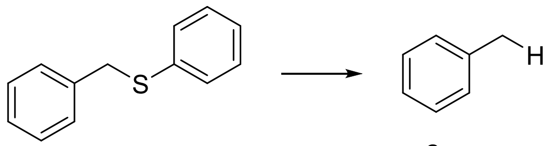

2a

Toluene (2a): Synthesis according to **Method A** and **B**. Quantification using HPLC (**Method A** = 99%, and **Method B** = 98% yield).

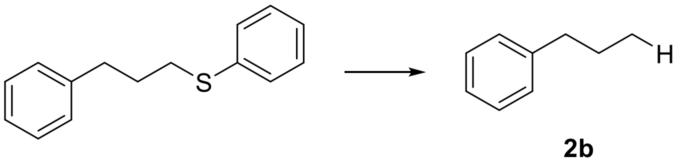

2b

n-Propylbenzene (2b): Synthesis according to **Method A** and **B**. Quantification using HPLC (**Method A** = 96%, and **Method B** = 89% yield).

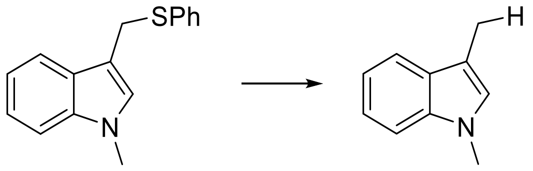

2c

1,3-Dimethylindole (2c): Synthesis according to **Method A** and **B**. Purified by column chromatography (7:3 P.E/EtOAc). **2c** was obtained as a yellow oil (**Method A** = 90%, and **Method B** = 97%). **^{1}H NMR** (400 MHz, CDCl3) δ 7.62 (d, J = 7.9 Hz, 1H), 7.36 – 7.21 (m, 2H), 7.15 (ddd, J = 7.9, 6.8, 1.2 Hz, 1H), 6.86 – 6.82 (m, 1H), 3.74 (s, 3H), 2.37 (s, 3H). **^{13}C NMR** (126 MHz, CDCl3) δ 137.1, 128.7, 126.6, 121.5, 119.0, 118.6, 110.1, 109.1, 32.5, 9.6. The spectroscopic data matched those reported in the literature.[3]

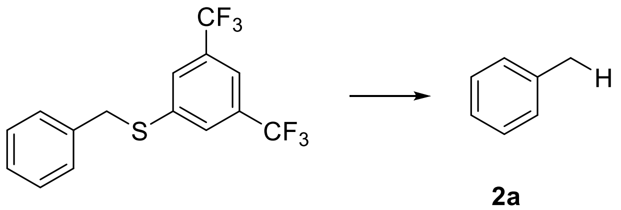

2a

Toluene (2a): Synthesis according to **Method A** and **B**. Quantification using HPLC (**Method A** = 92%, and **Method B** = 93% yield.)

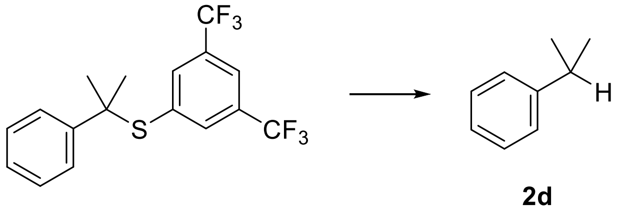

2d

Cumene (2d): Synthesis according to **Method A** and **B**. Quantification using HPLC (**Method A** = 76%, and **Method B** = 77% yield).

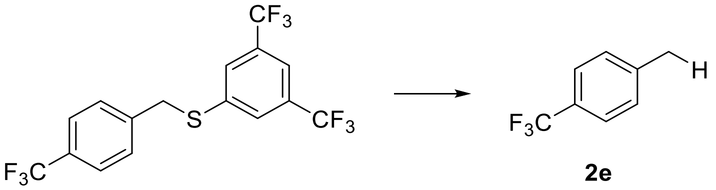

2e

4-Methylbenzotrifluoride (2e): Synthesis according to **Method A** and **B**. Quantification using HPLC (**Method A** = 87%, and **Method B** = 86% yield).

S2.2.2 Products of hydrodeoxygenation of alcohols

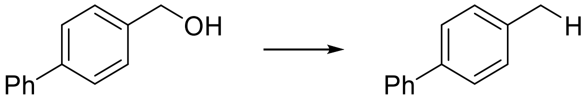

2f

4-phenyltoluene (2f): Synthesis according to **Method C** and **D**. Quantification using HPLC resulted in >99% of **2f** for both **Method C** and **D.**

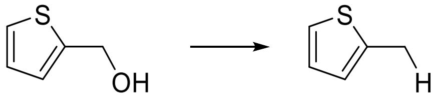

2g

2-methylthiophene (2g): Synthesis according to **Method C** and **D**. Quantification using GC-FID resulted in 18% of **2g** for **Method C** and 21% of **2g** for **conditions D**.

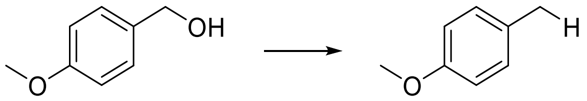

2h

4-methoxytoluene (2h): Synthesis according to **Method C** and **D**. Quantification using HPLC resulted in 63% of **2h** for **Method C** and 64% of **2h** for **conditions D**.

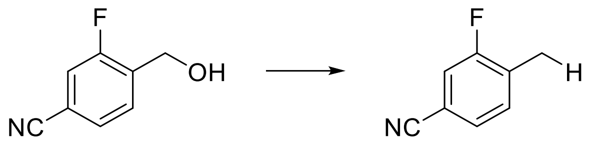

2i

3-fluoro-4-methylbenzonitrile (2i): Synthesis according to **Method C** and **D**. Quantification using HPLC resulted in 94% of **2i** for **Method C** and 92% of **2i** for **Method D**.

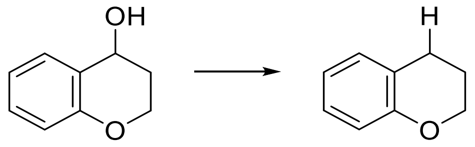

2j

Chromane (2j): Synthesis according to **Method C** and **D**. Quantification using HPLC resulted in 89% of **2j** for **Method C** and 92% of **2j** for **Method D**.

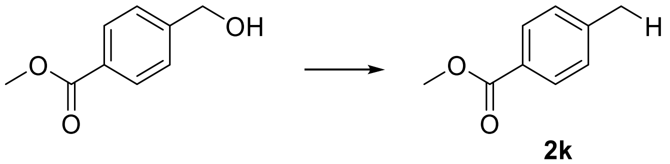

2k

Methyl p-toluate (2k): Synthesis according to **Method C** and **D**. Quantification using HPLC resulted in 81% of **2k** for **Method C** and 52% of **2k** for **Method D**.

S2.2.3 Products of electrochemical Birch reduction

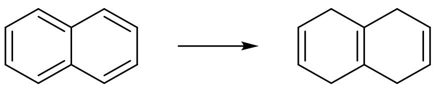

3a

1,4,5,8-tetrahydronaphthalene (3a): Synthesis according to **Method E** and **G** for 9 F. Quantification using qNMR analysis with 1,3,5-trimethoxybenzene as internal standard resulted in 57% of **3a** with **Method E** and 45% of **3a** with **Method G.**

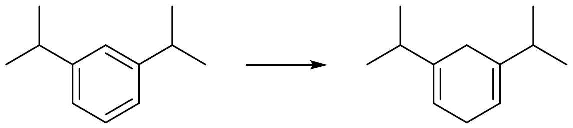

3b

1,5-diisopropylcyclohexa-1,4-diene (3b): Synthesis according to **Method F** and **G** for 5 F. Quantification using qNMR analysis with 1,3,5-trimethoxybenzene as internal standard resulted in 24% of **3b** with **Method F** and 13% of **3b** with **Method G.**

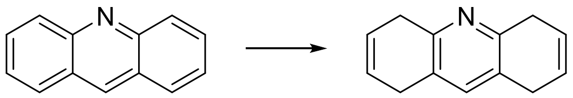

3c

1,4,4a,5,8,9a-hexahydroacridine (3c): Synthesis according to **Method E** and **G** for 15 F. Quantification using qNMR analysis with 1,3,5-trimethoxybenzene as internal standard resulted in 26% of **3c** with **Method E** and 21% of **3c** with **Method G.**

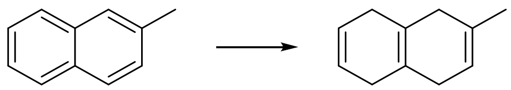

3d

2-methyl-1,4,5,8-tetrahydronaphthalene (3d): Synthesis according to **Method E** and **G** for 8 F. Quantification using qNMR analysis with 1,3,5-trimethoxybenzene as internal standard resulted in 71% of **3d** with **Method E** and 40% of **3d** with **Method G.**

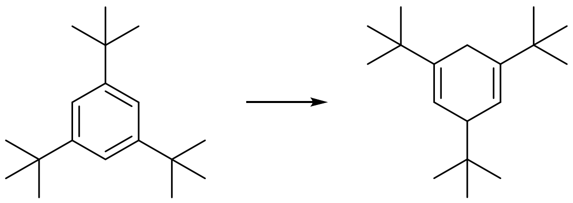

3e

1,3,5-tri-tert-butylcyclohexa-1,4-diene (3e): Synthesis according to **Method F** and **G** for 10 F. Quantification using qNMR analysis with 1,3,5-trimethoxybenzene as internal standard resulted in 66% of **3e** with **Method F** and 77% of **3e** with **Method G.**

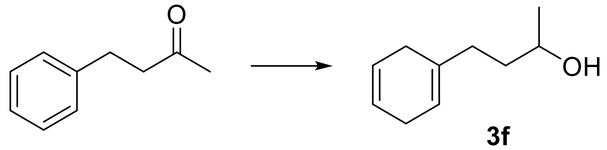

3f

4-(cyclohexa-1,4-dien-1-yl)butan-2-ol (3f): Synthesis according to **Method E** and **G** from 4-phenylbutan-2-one for 5 F. Quantification using qNMR analysis with 1,3,5-trimethoxybenzene as internal standard resulted in 32% of **3f** with **Method E** and 41% of **3f** with **Method G.**

S2.2.3.1 *NMR spectra for electrochemical Birch reduction*

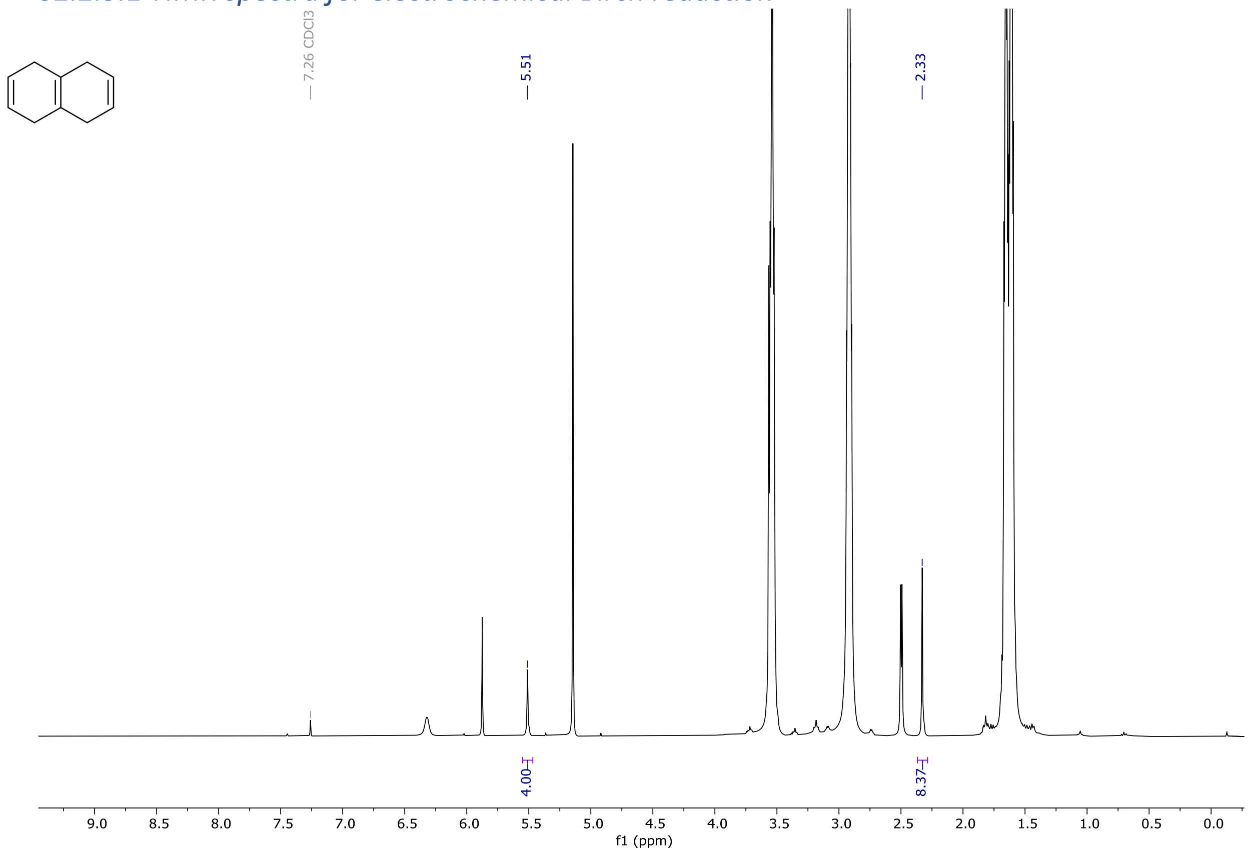

Figure S1. ^{1}H-NMR spectrum for reduction of naphthalene using **Method E**. The signals observed at 5.51 and 2.33 ppm were assigned 1,4,5,8-tetrahydronaphthalene **3a** and matched those reported in literature.[2]

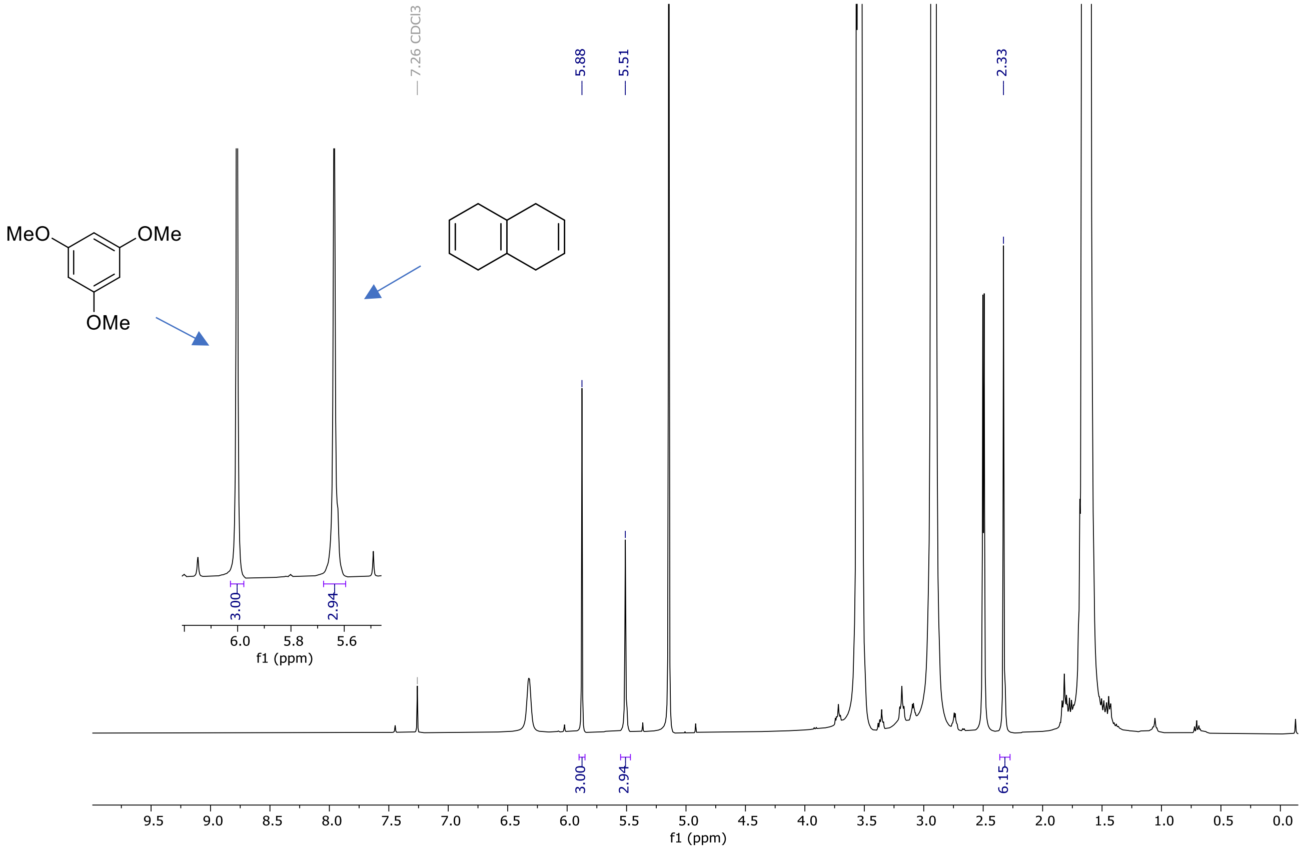

Figure S2. ^{1}H-NMR spectrum for naphthalene reduction using **Method E**. The signals observed at 6.09 and 5.51 ppm were assigned to 1,3,5-TMB and 1,4,5,8-tetrahydronaphthalene **3a**, respectively and resulted in 57% of **3a**. The spectroscopic data matched those reported in literature. [2]

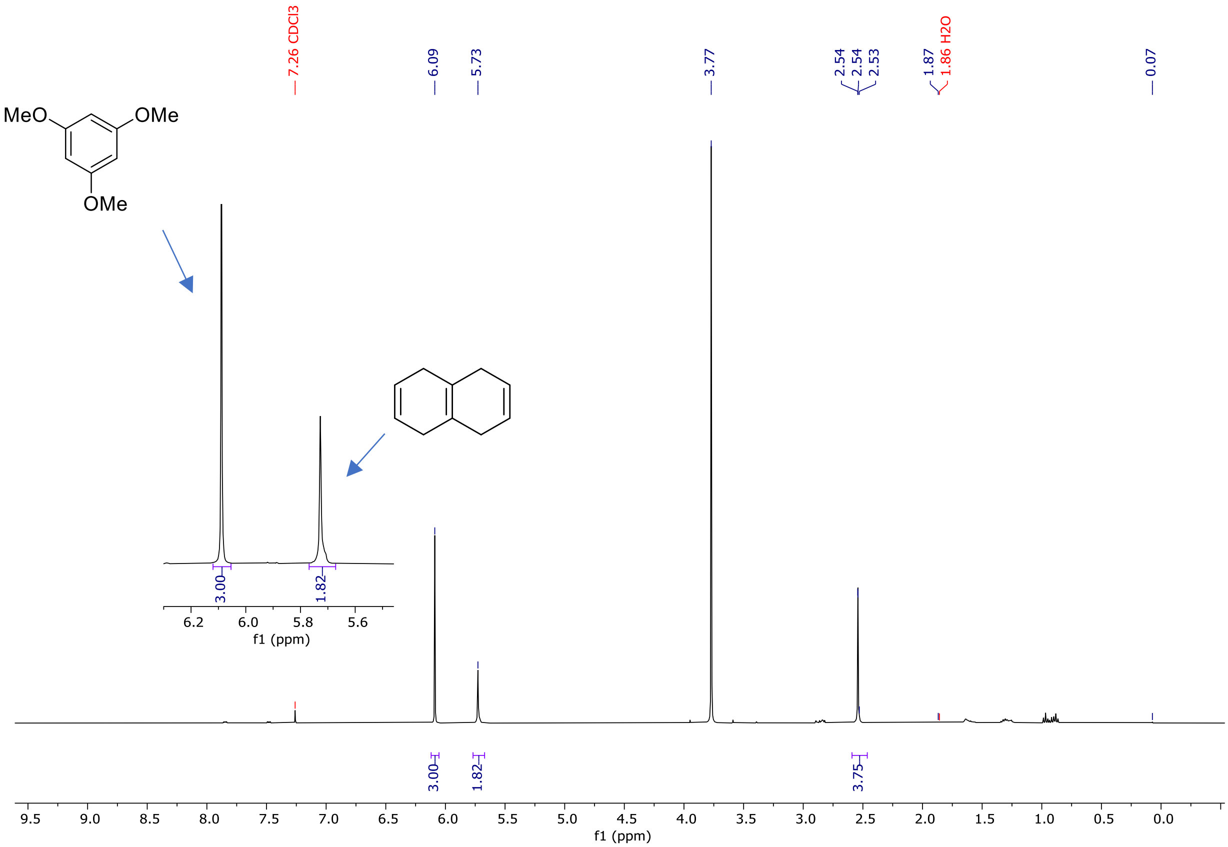

Figure S3. ^{1}H-NMR spectrum for naphthalene reduction with **Method G**. The signals observed at 6.09 and 5.73 ppm were assigned to 1,3,5-TMB and 1,4,5,8-tetrahydronaphthalene **3a**, respectively resulting in 46% of **3a**. The spectroscopic data matched those reported in literature. [2]

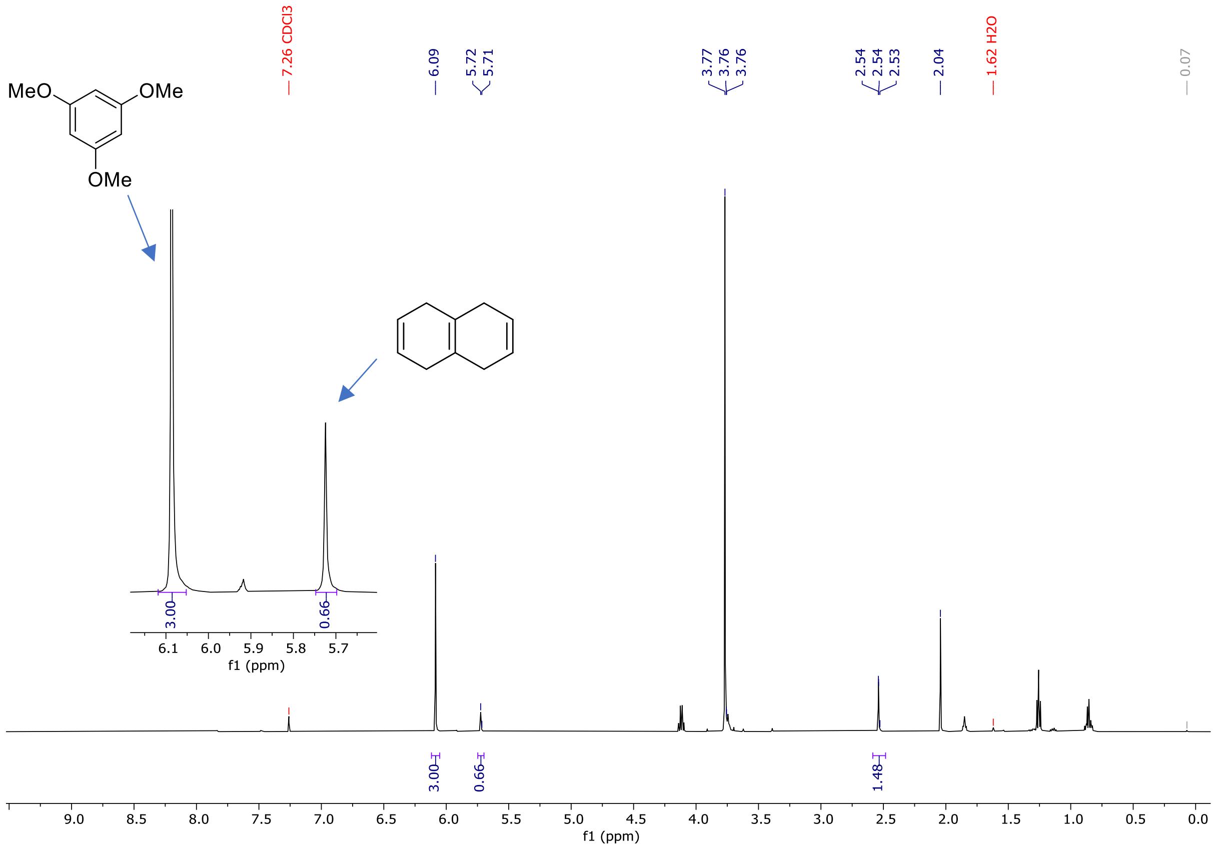

Figure S4. ^{1}H-NMR spectrum for naphthalene reduction using **Method G** without addition of Bu_4NBH_4. The signals observed at 6.09 and 5.72 ppm were assigned to 1,3,5-TMB and 1,4,5,8-tetrahydronaphthalene **3a**, respectively and resulted in 17% yield of **3a**. The spectroscopic data matched those reported in literature. [2]

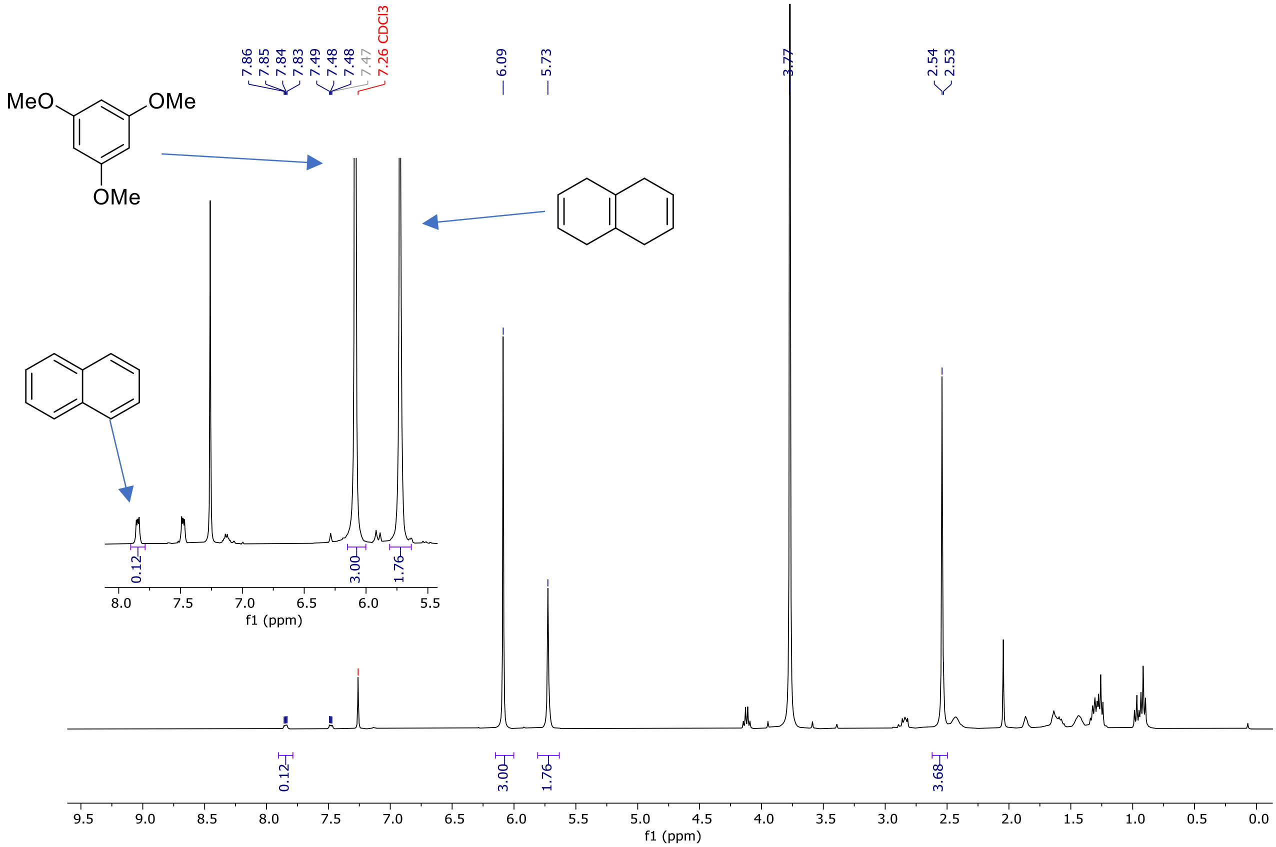

Figure S5. ^{1}H-NMR spectrum for naphthalene reduction using **Method G** with a graphite anode. The signals observed at 6.09 and 3.77 ppm were assigned to 1,3,5-TMB, the signals at 7.47-7.86 ppm were assigned to naphthalene and the signals at 5.72 and 2.54 ppm were assigned to 1,4,5,8-tetrahydronaphthalene **3a** which formed in 44% yield. The spectroscopic data matched those reported in literature. [2]

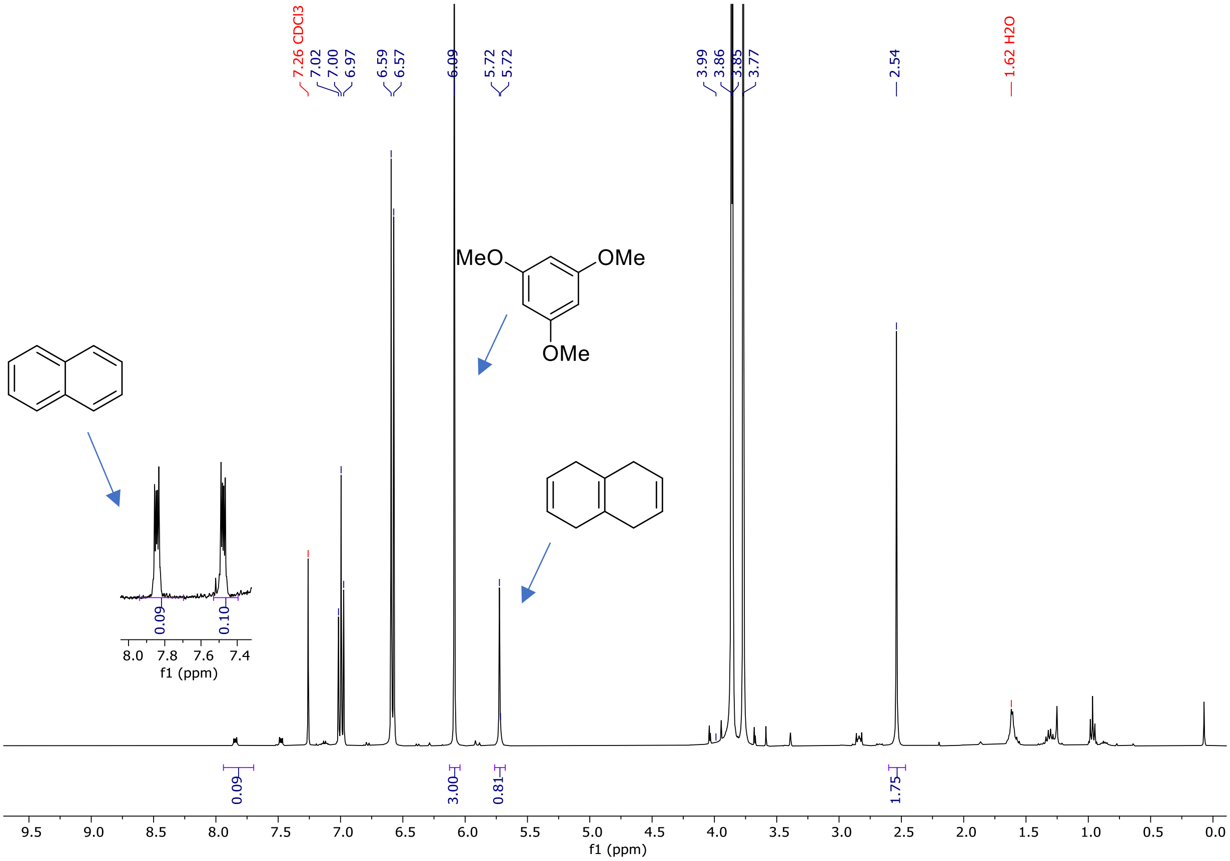

Figure S6. ^{1}H-NMR spectrum for naphthalene reduction using **Method G** with a BDD anode. The signals observed at 6.09 ppm were assigned to 1,3,5-TMB, the signals at 7.47-7.86 ppm were assigned to naphthalene and the signals at 5.72 and 2.54 ppm were assigned to 1,4,5,8-tetrahydronaphthalene **3a** which formed in 20% yield. The spectroscopic data matched those reported in literature. [2]

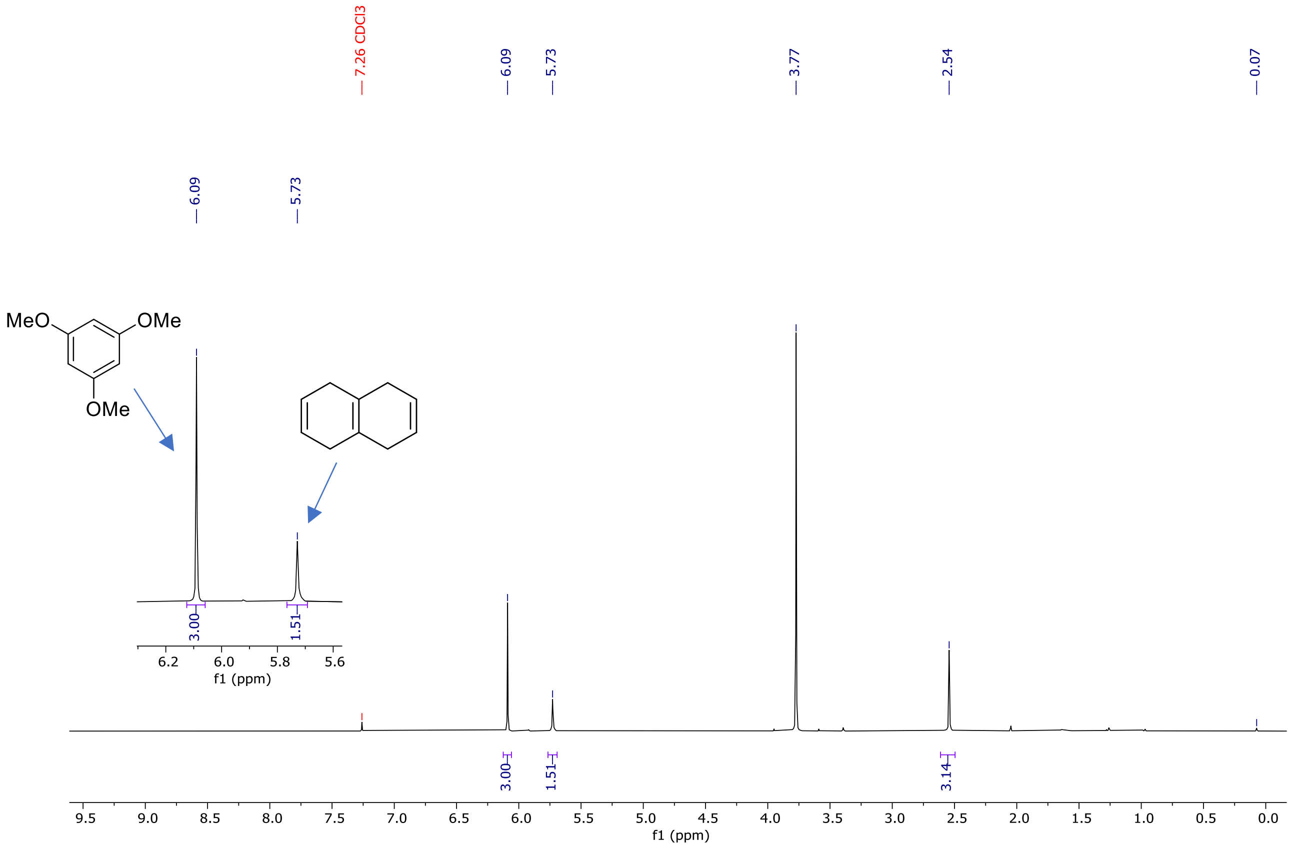

Figure S7. ^{1}H-NMR spectrum for naphthalene reduction using **Method G** with 0.5 equiv. $TBABH_4$. The signals observed at 6.09 and 3.77 ppm were assigned to 1,3,5-TMB, the signals at 7.47-7.86 ppm were assigned to naphthalene and the signals at 5.72 and 2.54 ppm were assigned to 1,4,5,8-tetrahydronaphthalene **3a** which formed in 38% yield. The spectroscopic data matched those reported in literature.[2]

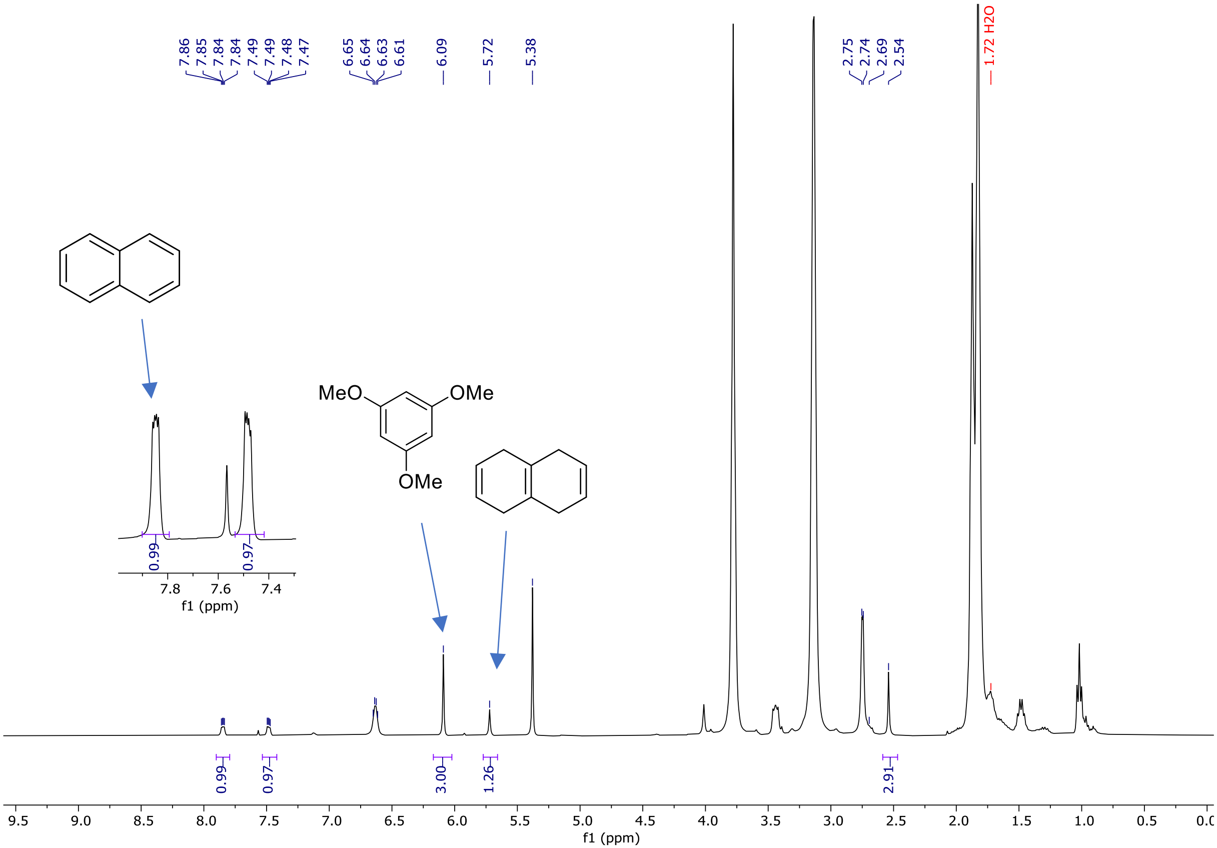

Figure S8. ^{1}H-NMR spectrum for naphthalene reduction using **Method G** with 4.5 F. The signals observed at 6.09 and 3.77 ppm were assigned to 1,3,5-TMB, the signals at 7.47-7.86 ppm were assigned to naphthalene and the signals at 5.72 and 2.54 ppm were assigned to 1,4,5,8-tetrahydronaphthalene **3a** which formed in 32% yield. The spectroscopic data matched those reported in literature. [2]

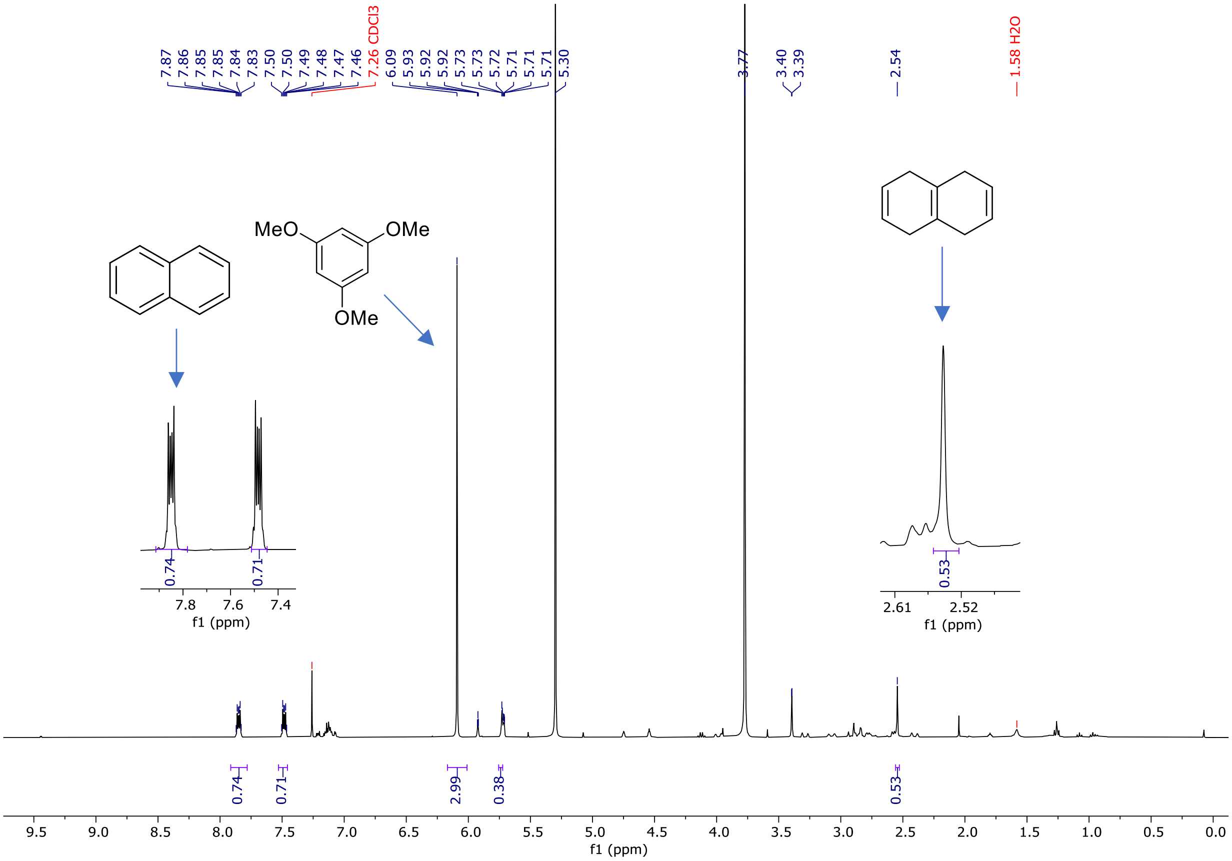

Figure S9. ^{1}H-NMR spectrum for naphthalene reduction using **Method G** at room temperature. The signals observed at 6.09 and 3.77 ppm were assigned to 1,3,5-TMB, the signals at 7.47-7.86 ppm were assigned to naphthalene and the signals at 5.72 and 2.54 ppm were assigned to 1,4,5,8-tetrahydronaphthalene **3a** which formed in 18% yield. The spectroscopic data matched those reported in literature.[2]

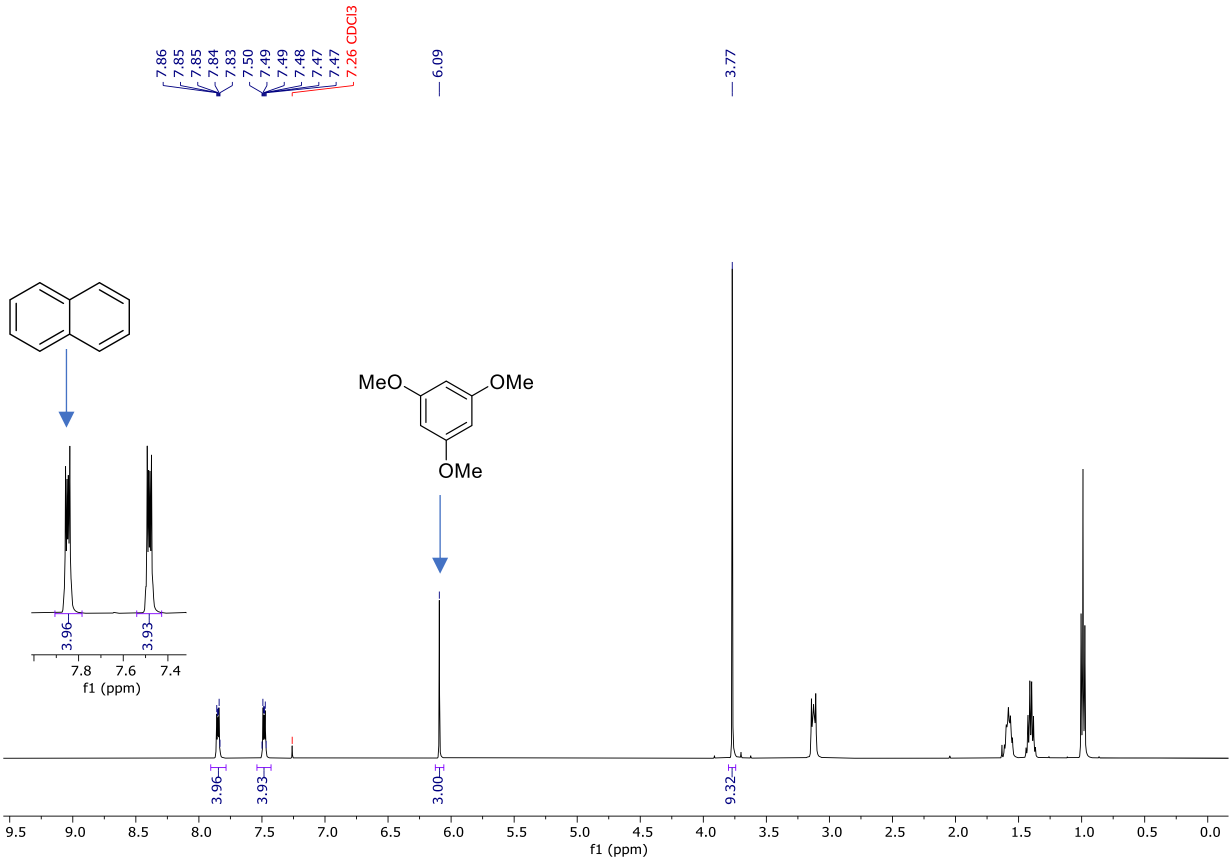

Figure S10. ^{1}H-NMR spectrum for naphthalene reduction using **Method G** without any applied current. The signals observed at 6.09 and 3.77 ppm were assigned to TMB, the signals at 7.47-7.86 ppm were assigned to naphthalene **1m.** No signals corresponding to product 1,4,5,8-tetrahydronaphthalene **3a** was identified. The spectroscopic data matched those reported in literature.[2]

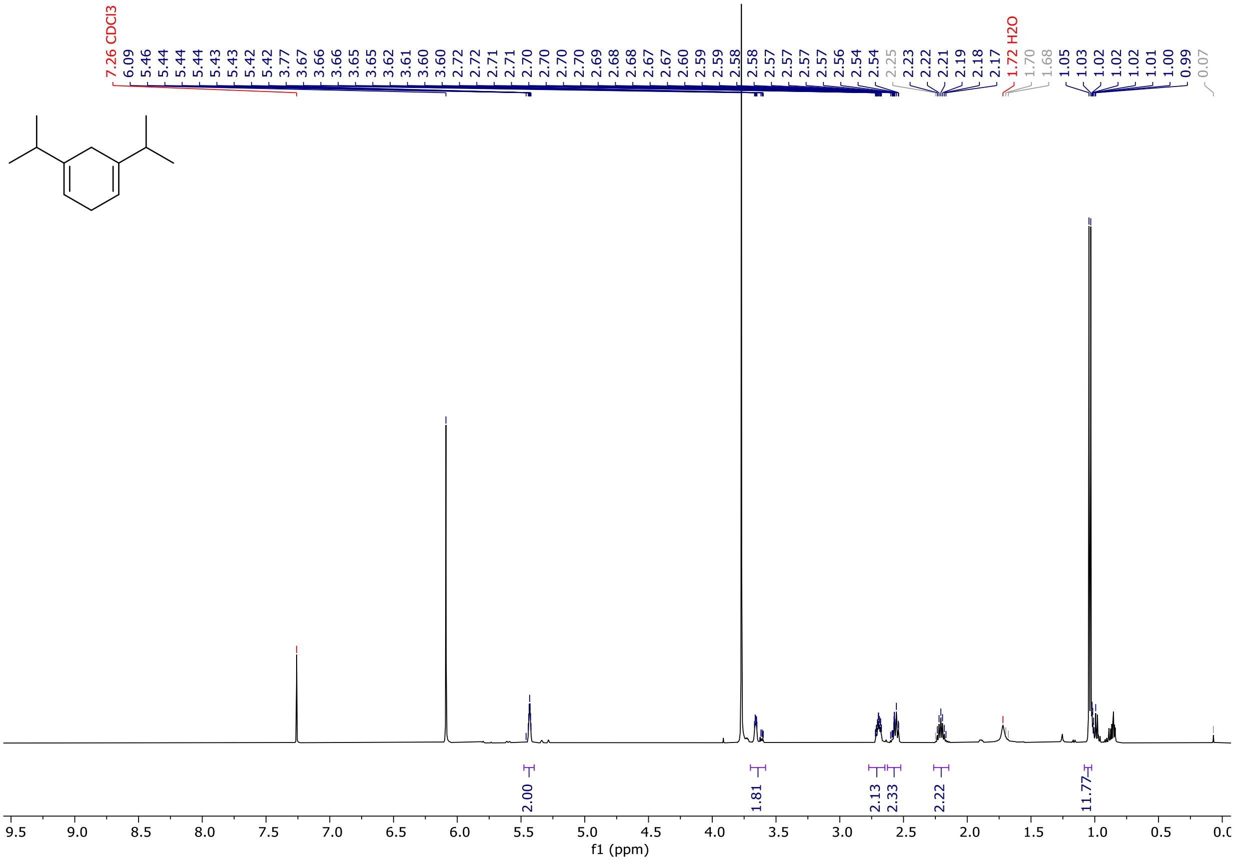

Figure S11. ^{1}H-NMR spectrum for 1,3-diisopropylbenzene reduction using **Method F**. The signals observed at δ 5.43, 2.77 – 2.65, 2.63 – 2.52, 2.20, 1.04 ppm were assigned to 1,5-diisopropylcyclohexa-1,4-diene **3b**. The spectroscopic data matched those reported in literature.[2]

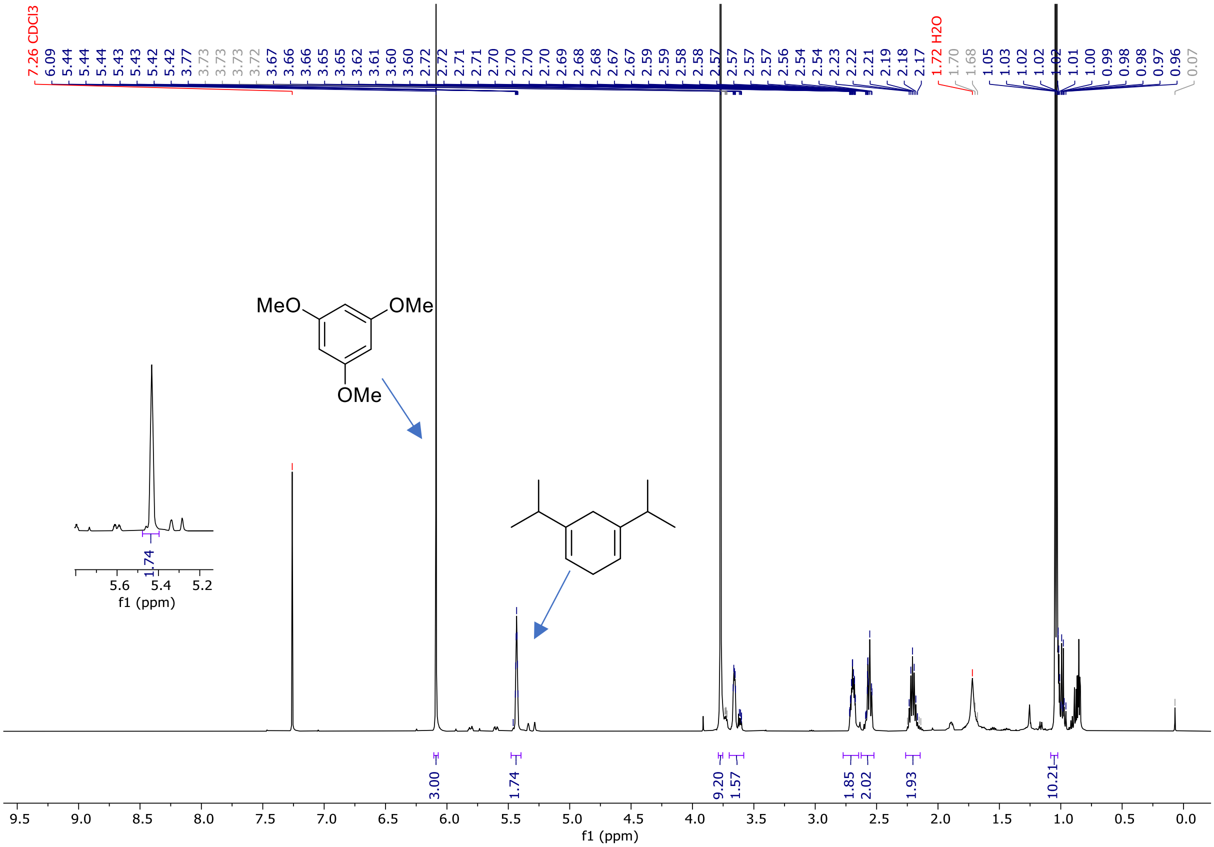

Figure S12. ^{1}H-NMR spectrum for 1,3-diisopropylbenzene reduction using **Method F**. The signals observed at 6.09 and 3.75 ppm were assigned to 1,3,5-TMB and the signals observed at 5.43, 2.77 – 2.65, 2.63 – 2.52, 2.20, 1.04 ppm were assigned to 1,5-diisopropylcyclohexa-1,4-diene **3b** which resulting in 25% yield. The spectroscopic data matched those reported in literature.[2]

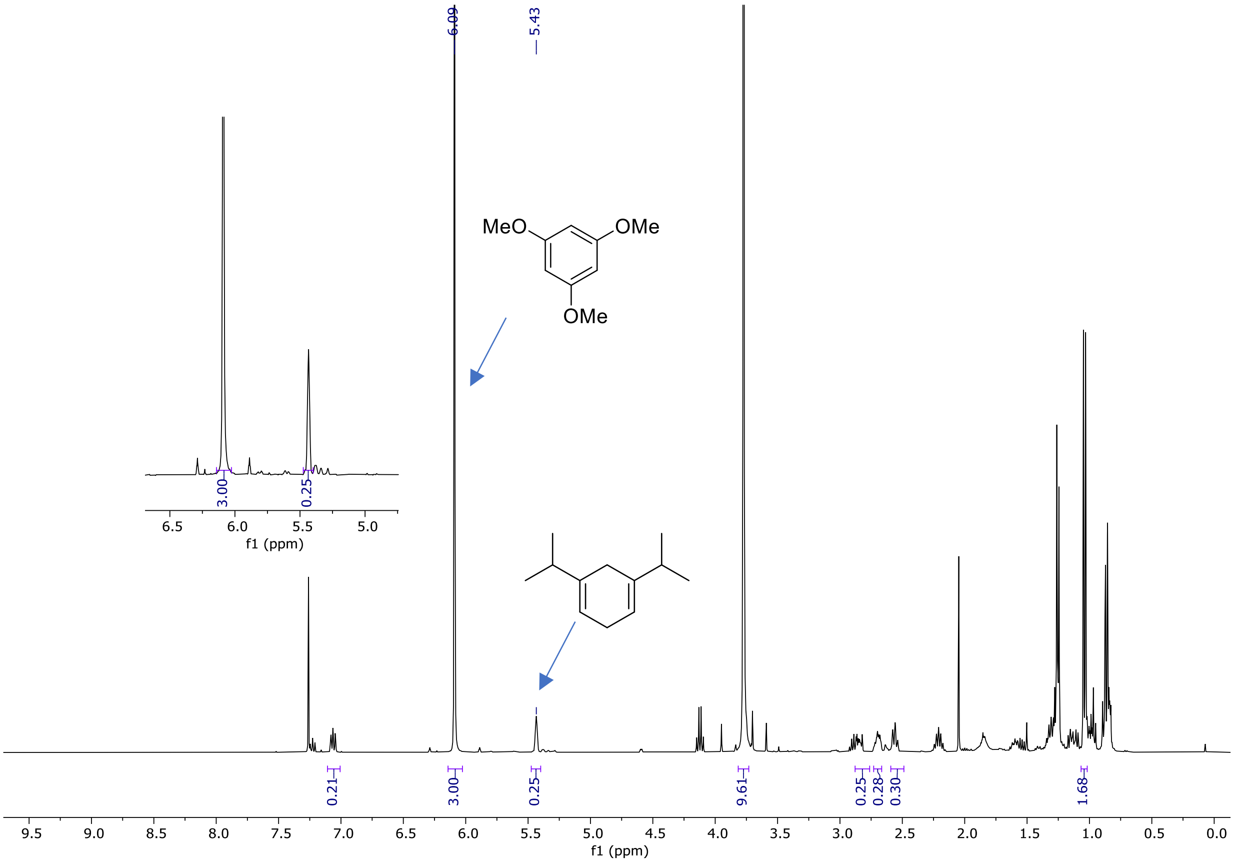

Figure S13. ^{1}H-NMR spectrum for 1,3-diisopropylbenzene reduction using **Method G**. The signals observed at 6.09 and 3.75 ppm were assigned to 1,3,5-TMB and the signals observed at 5.43, 2.77 – 2.65, 2.63 – 2.52, 2.20, 1.04 ppm were assigned to 1,5-diisopropylcyclohexa-1,4-diene **3b** which resulting in 13% yield. The spectroscopic data matched those reported in literature.[2]

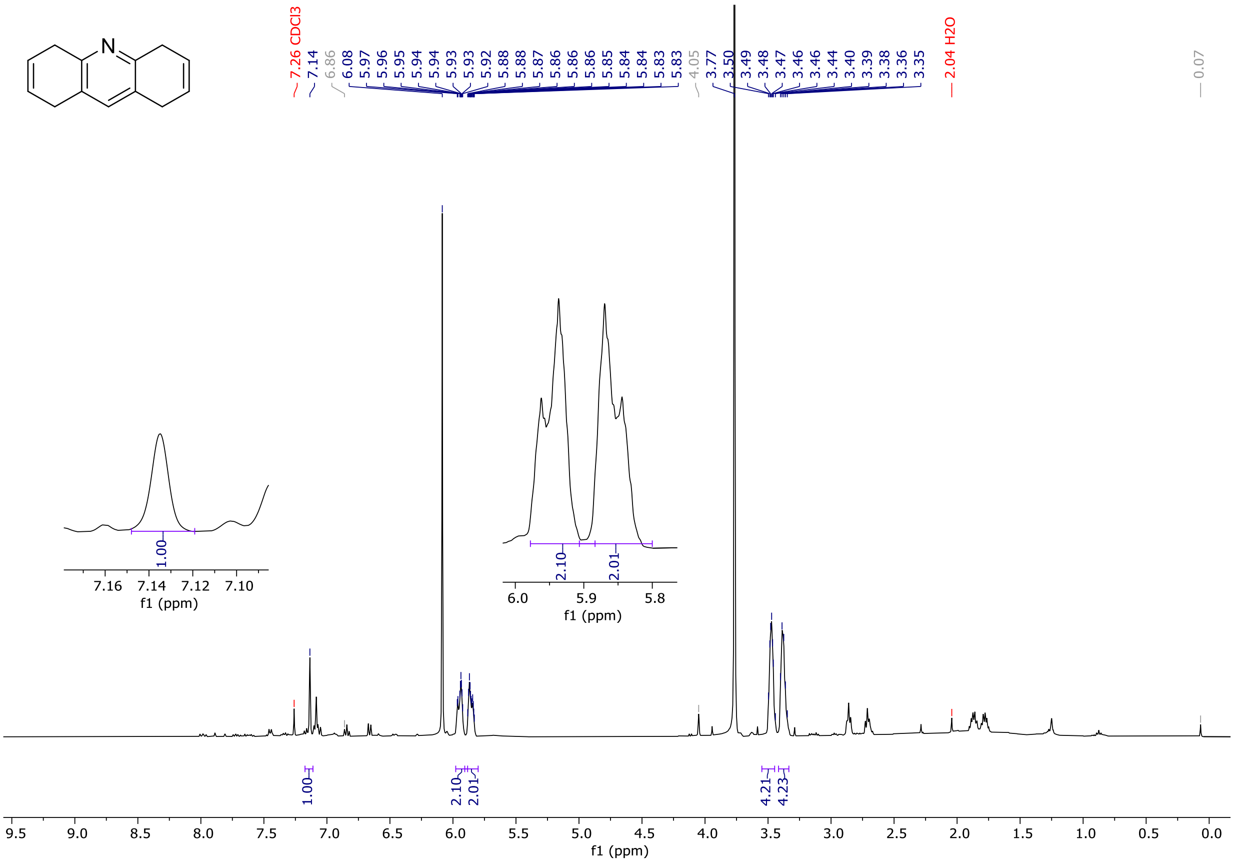

Figure S14. ^{1}H-NMR spectrum for acridine reduction using **Method E**. The signals observed at 7.14, 5.98 – 5.88, 5.91 – 5.80, 3.55 – 3.45, 3.37 ppm were assigned to and 1,4,4a,5,8,9a-hexahydroacridine **3c**. The spectroscopic data matched those reported in literature.[2]

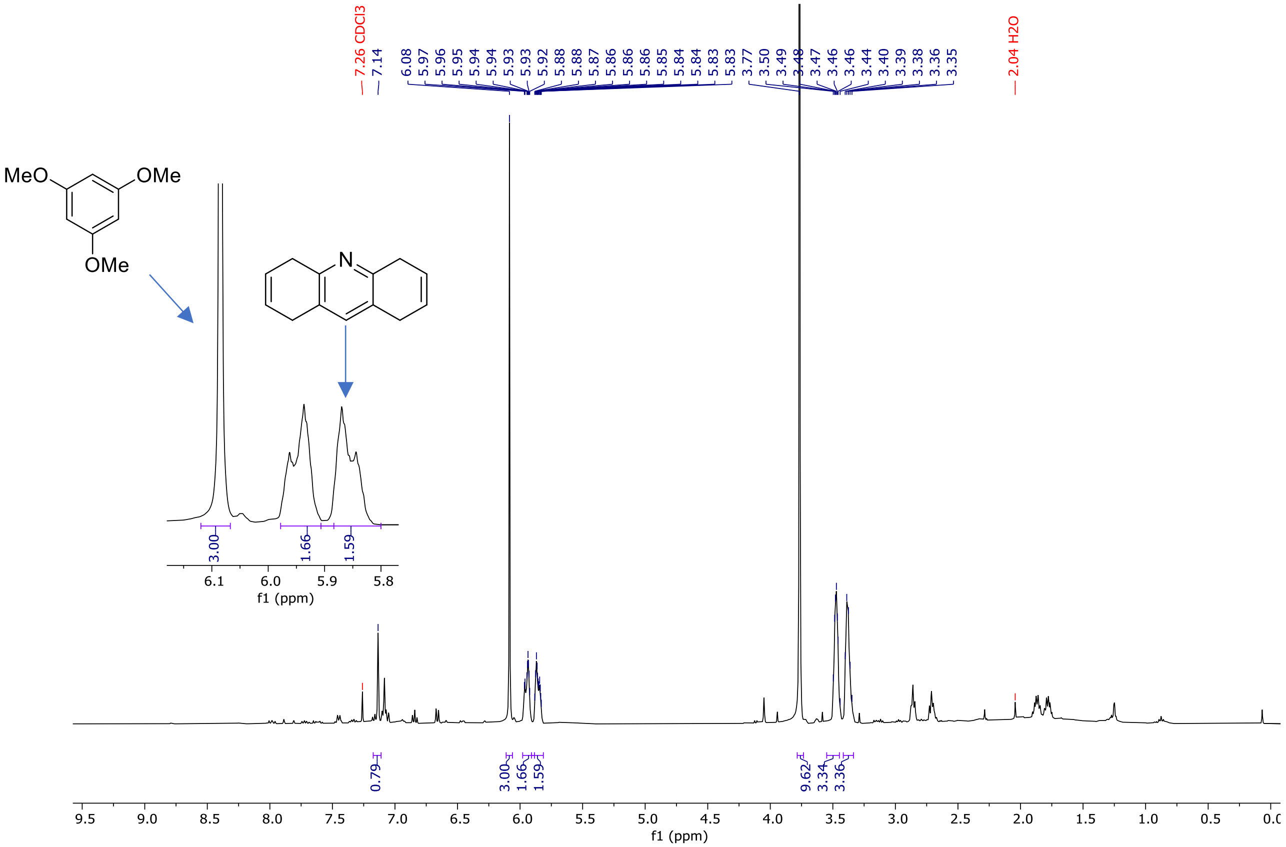

Figure S15. ^{1}H-NMR spectrum for acridine reduction using **Method E**. The signals observed at δ 6.09 and 3.75 ppm were assigned to 1,3,5-TMB and the signals observed at 7.14, 5.98 – 5.88, 5.91 – 5.80, 3.55 – 3.45, 3.37 ppm were assigned to 1,4,4a,5,8,9a-hexahydroacridine **3c** which resulted in 20% yield. The spectroscopic data matched those reported in literature. [2]

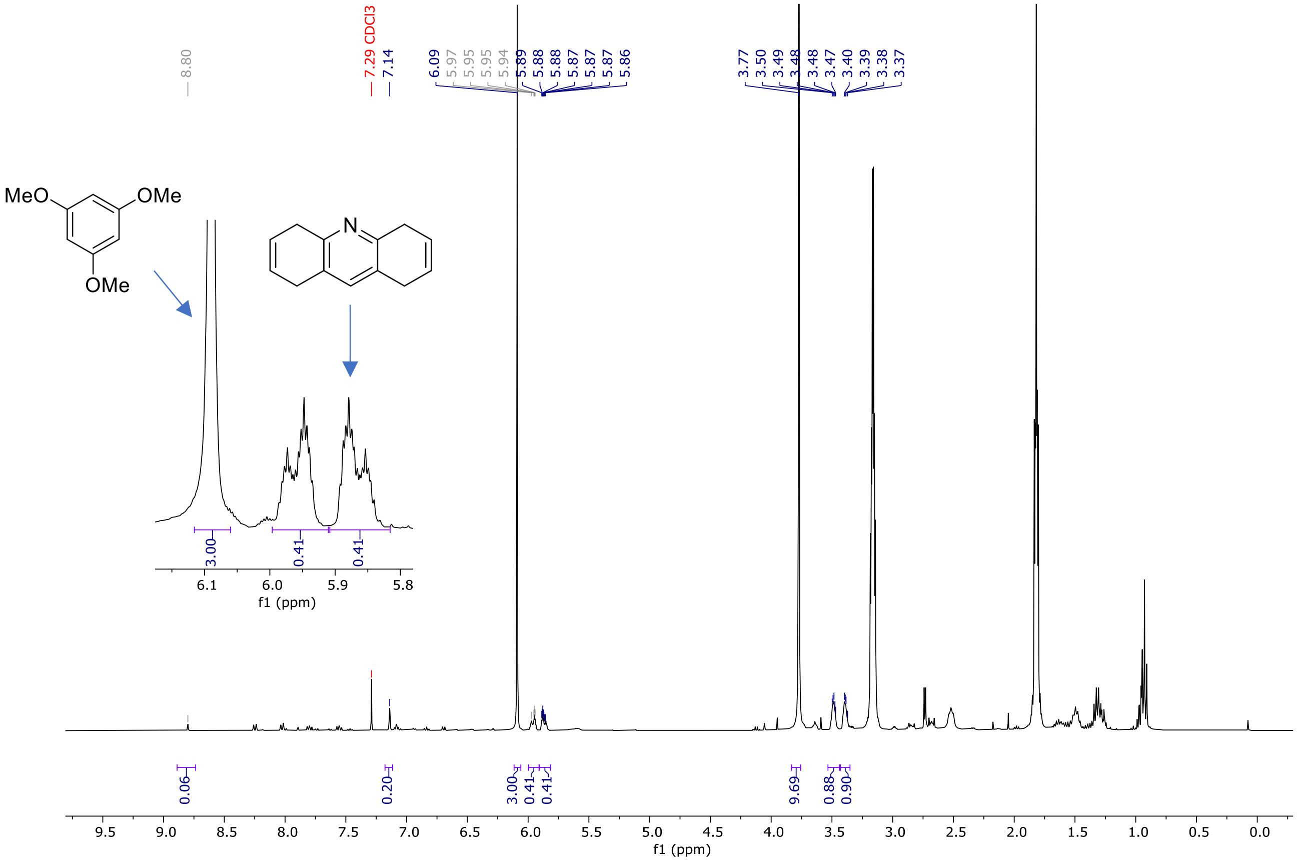

Figure S16. ^{1}H-NMR spectrum for acridine **3c** reduction using **Method G**. The signals observed at 6.09 and 3.75 ppm were assigned to 1,3,5-TMB and the signals observed at 7.14, 5.98 – 5.88, 5.91 – 5.80, 3.55 – 3.45, 3.37 ppm were assigned to 1,4,4a,5,8,9a-hexahydroacridine **3c** which resulted in 21% yield. The spectroscopic data matched those reported in literature. [2]

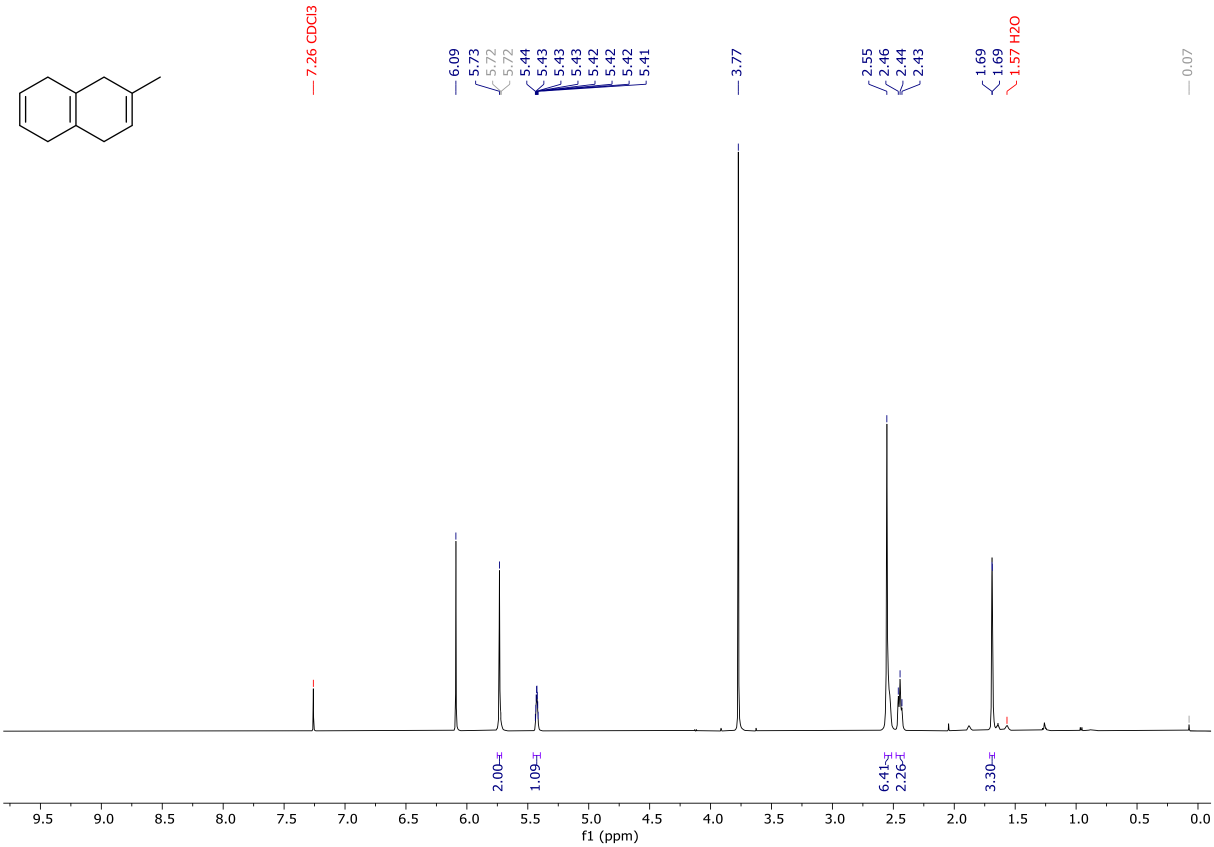

Figure S17. ^{1}H-NMR spectrum for 2-methylnaphthalene reduction using **Method E**. The signals observed at 5.73, 5.43, 2.55, 2.4, 1.69 ppm were assigned to 2-methyl-1,4,5,8-tetrahydronaphthalene **3d**. The spectroscopic data matched those reported in literature. [2]

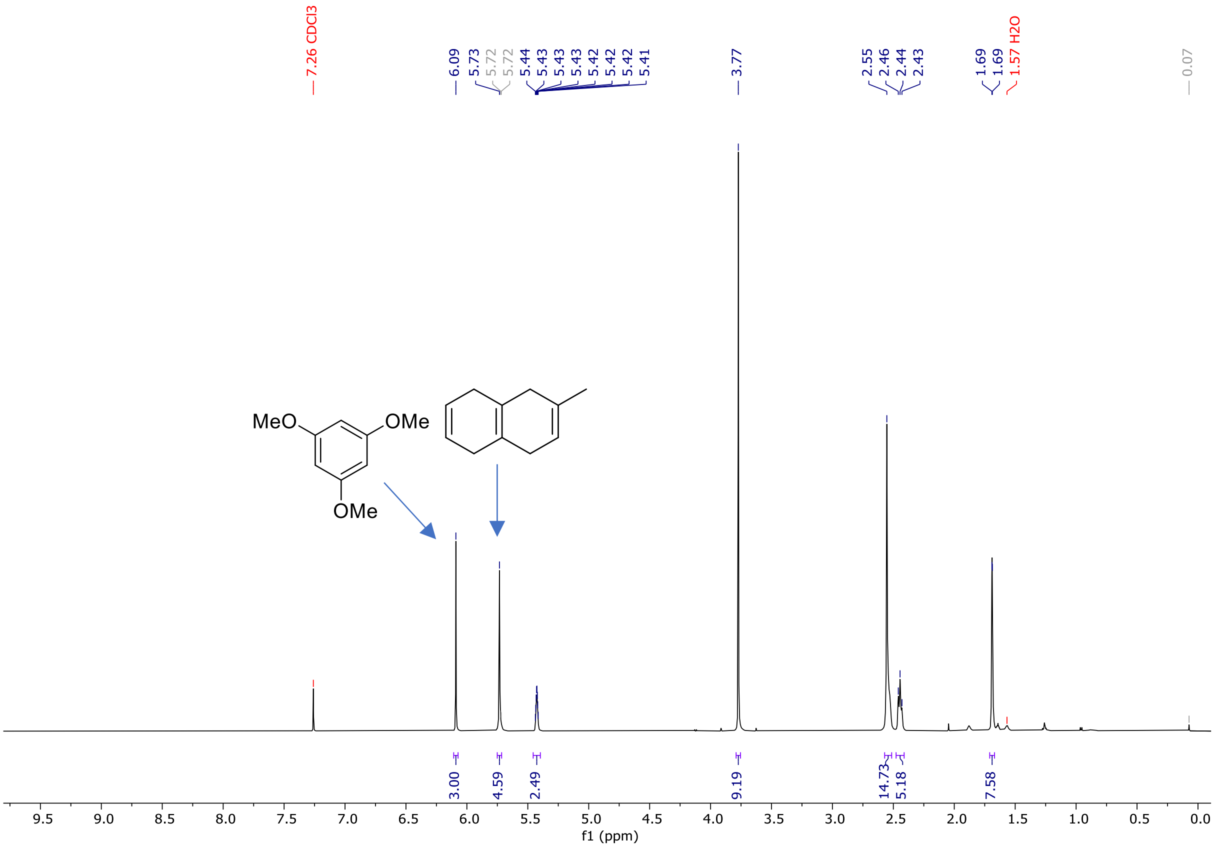

Figure S18. ^{1}H-NMR spectrum for 2-methylnaphthalene reduction using **Method E**. The signals observed at 6.09 and 3.75 ppm were assigned to 1,3,5-TMB and the signals observed at 5.73, 5.43, 2.55, 2.4, 1.69 ppm were assigned to 2-methyl-1,4,5,8-tetrahydronaphthalene **3d** which resulted in 71% yield. The spectroscopic data matched those reported in literature. [2]

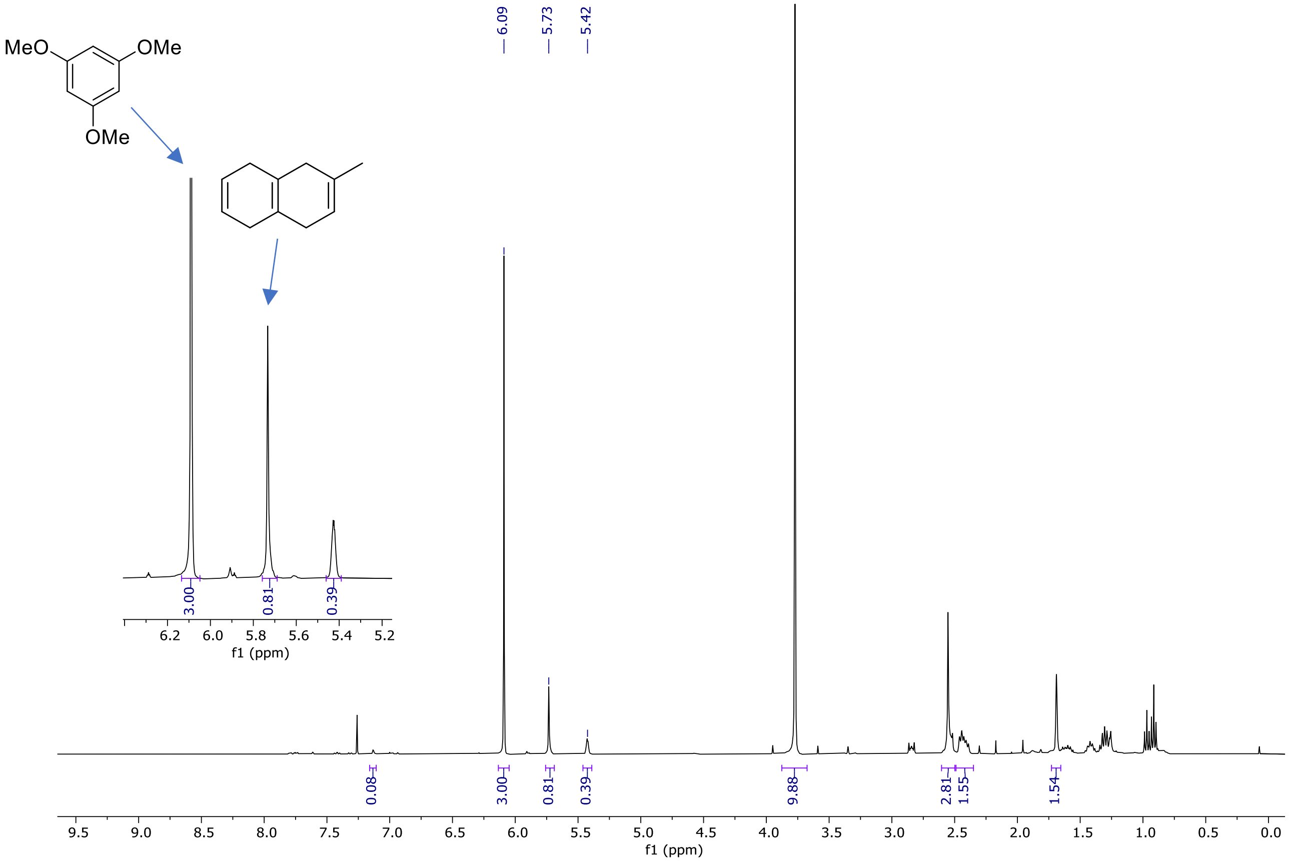

S

Figure S19. ^{1}H-NMR spectrum for 2-methylnaphthalene reduction using **Method G**. The signals observed at 6.09 and 3.75 ppm were assigned to 1,3,5-TMB and the signals observed at 5.73, 5.43, 2.55, 2.4, 1.69 ppm were assigned to 2-methyl-1,4,5,8-tetrahydronaphthalene **2d** which resulted in 40% yield. The spectroscopic data matched those reported in literature. [2]

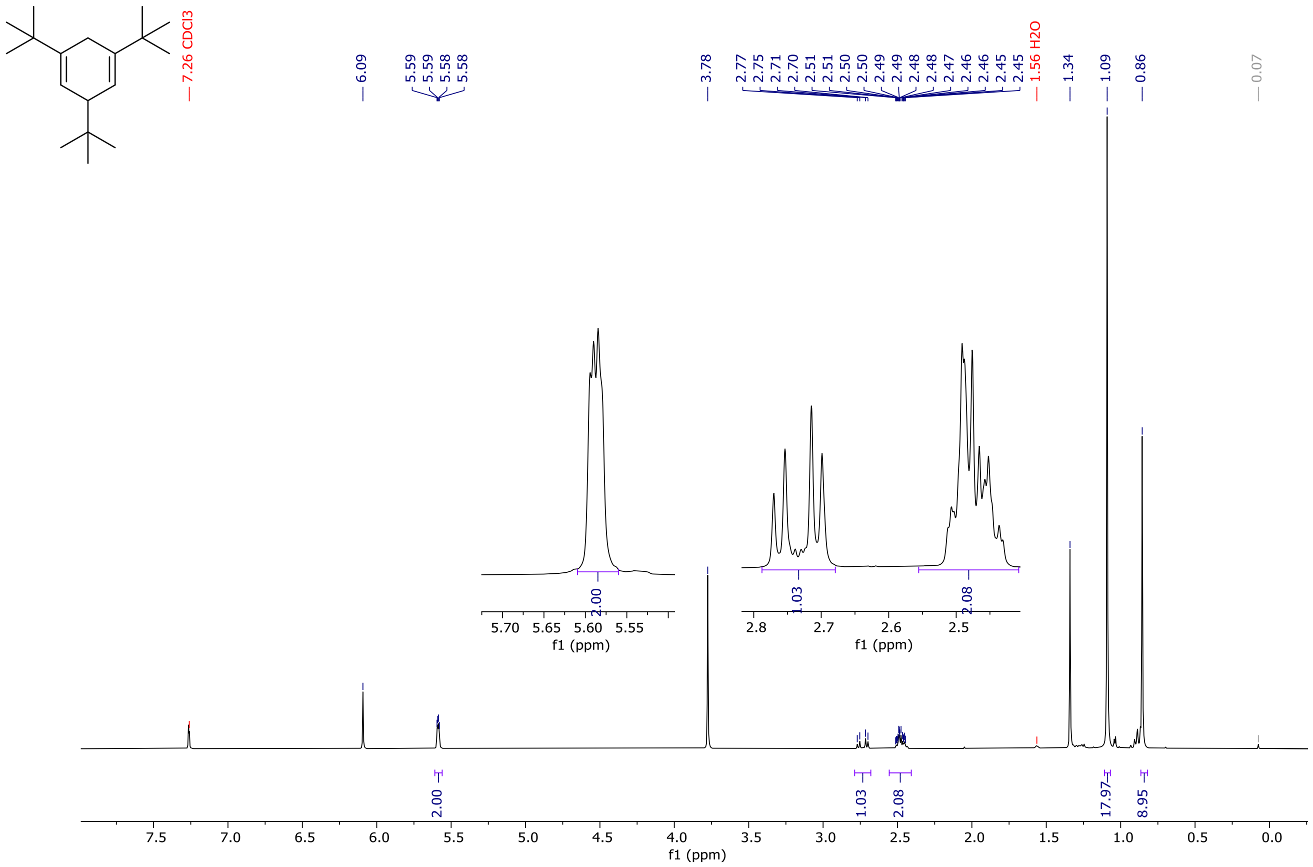

Figure S20. ^{1}H-NMR spectrum for 1,3,5-tri-tert-butylbenzene reduction using **Method F**. The signals observed at 5.58-5.59, 2.7-2.77, 2.45-2.51, 1.09 and 0.86 ppm were assigned to 1,3,5-tri-tert-butylcyclohexa-1,4-diene **3e**. The spectroscopic data matched those reported in literature. [2]

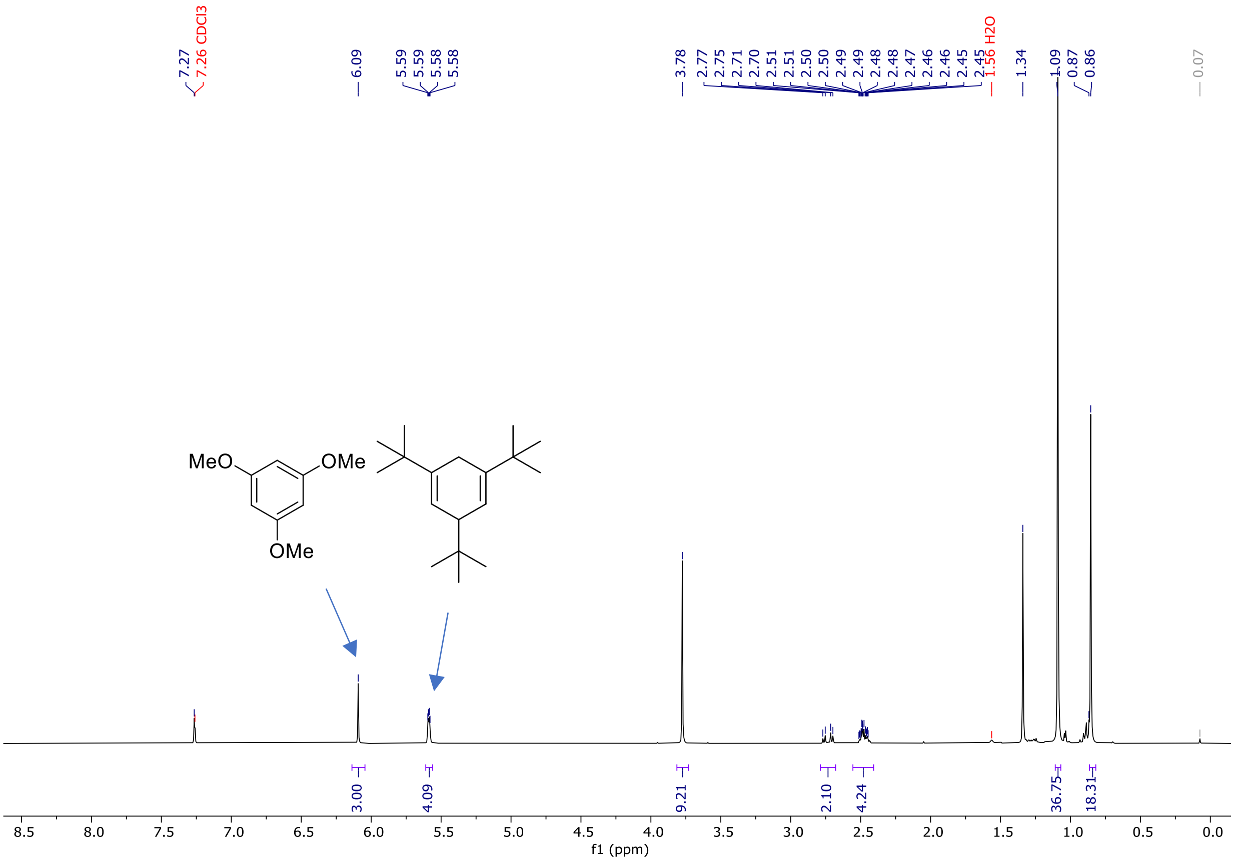

Figure S21. ^{1}H-NMR spectrum for 1,3,5-tri-tert-butylbenzene reduction using **Method F**. The signals observed at 6.09 and 3.78 ppm were assigned to 1,3,5-TMB and the signals 5.58-5.59, 2.7-2.77, 2.45-2.51, 1.09 and 0.86 ppm were assigned to 1,3,5-tri-tert-butylcyclohexa-1,4-diene **3e** which formed in 66% yield. The spectroscopic data matched those reported in literature. [2]

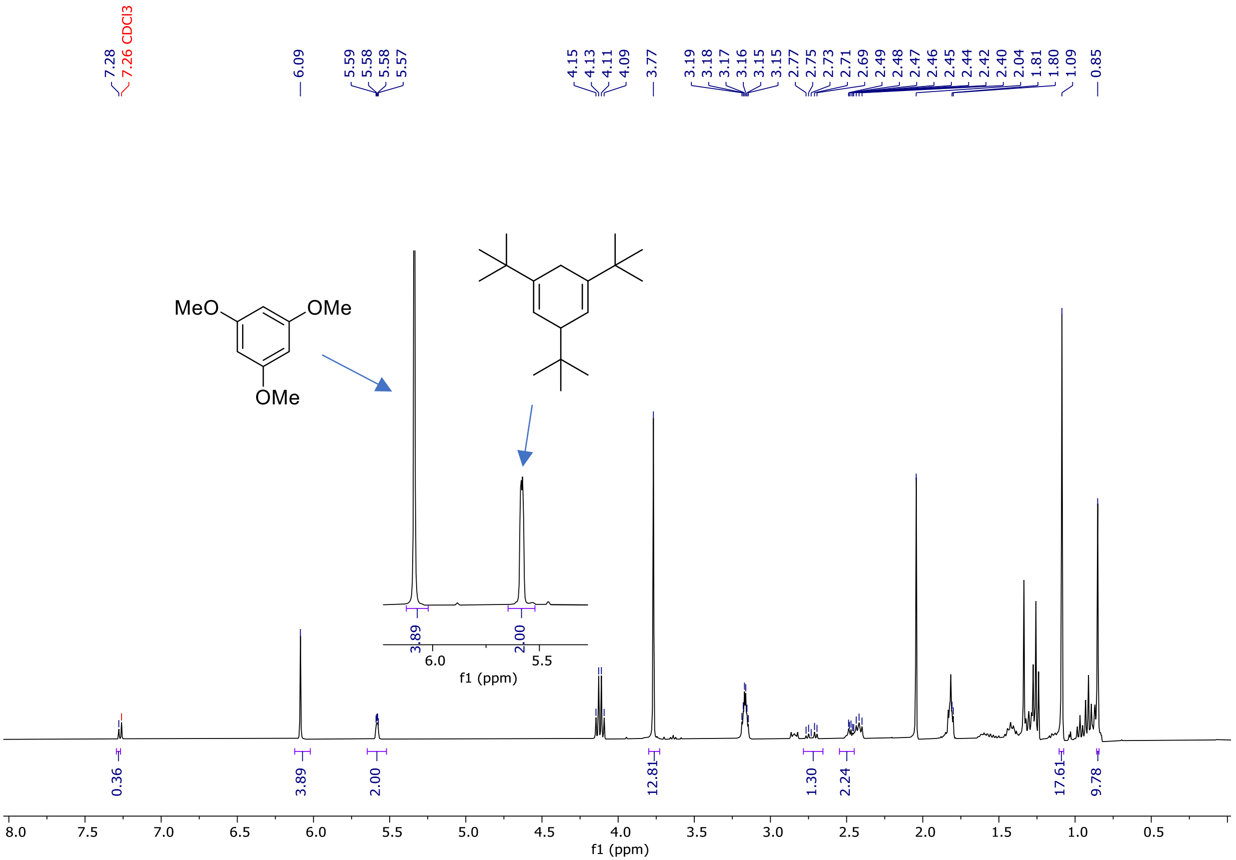

Figure S22. ^{1}H-NMR spectrum for 1,3, 5-tri-tert-butylbenzene reduction using **Method G**. The signals observed at 6.09 and 3.78 ppm were assigned to 1,3,5-TMB and the signals 5.58-5.59, 2.7-2.77, 2.45-2.51, 1.09 and 0.86 ppm were assigned to 1,3,5-tri-tert-butylcyclohexa-1,4-diene **3e** which formed in 77% yield. The spectroscopic data matched those reported in literature. [2]

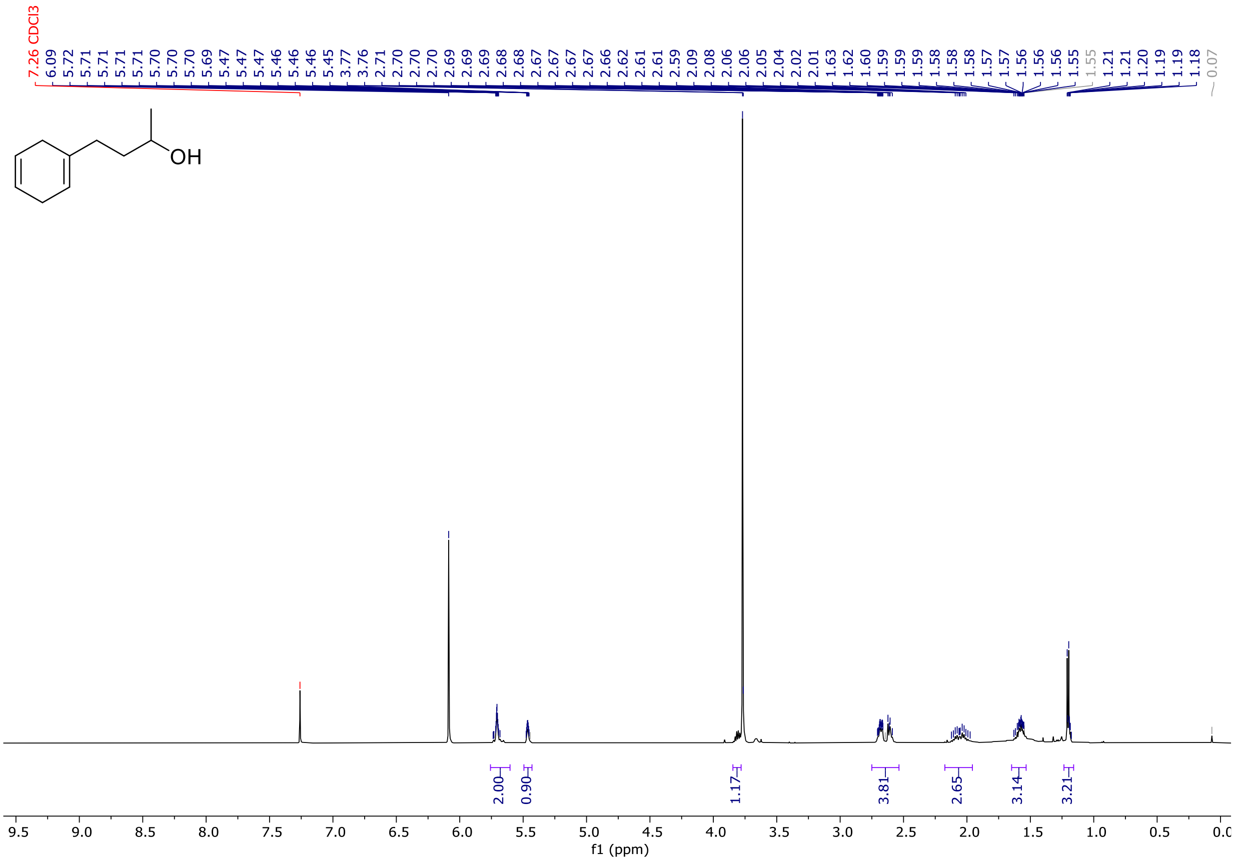

Figure S23. ^{1}H-NMR spectrum for 4-phenylbutan-2-ol reduction using **Method E**. The signals observed at 5.76 – 5.60, 5.46, 3.82, 2.75 – 2.54, 2.04, 1.65 – 1.53 and 1.23 – 1.16 ppm were assigned to 4-(cyclohexa-1,4-dien-1-yl)butan-2-ol **3f**. The spectroscopic data matched those reported in literature. [2]

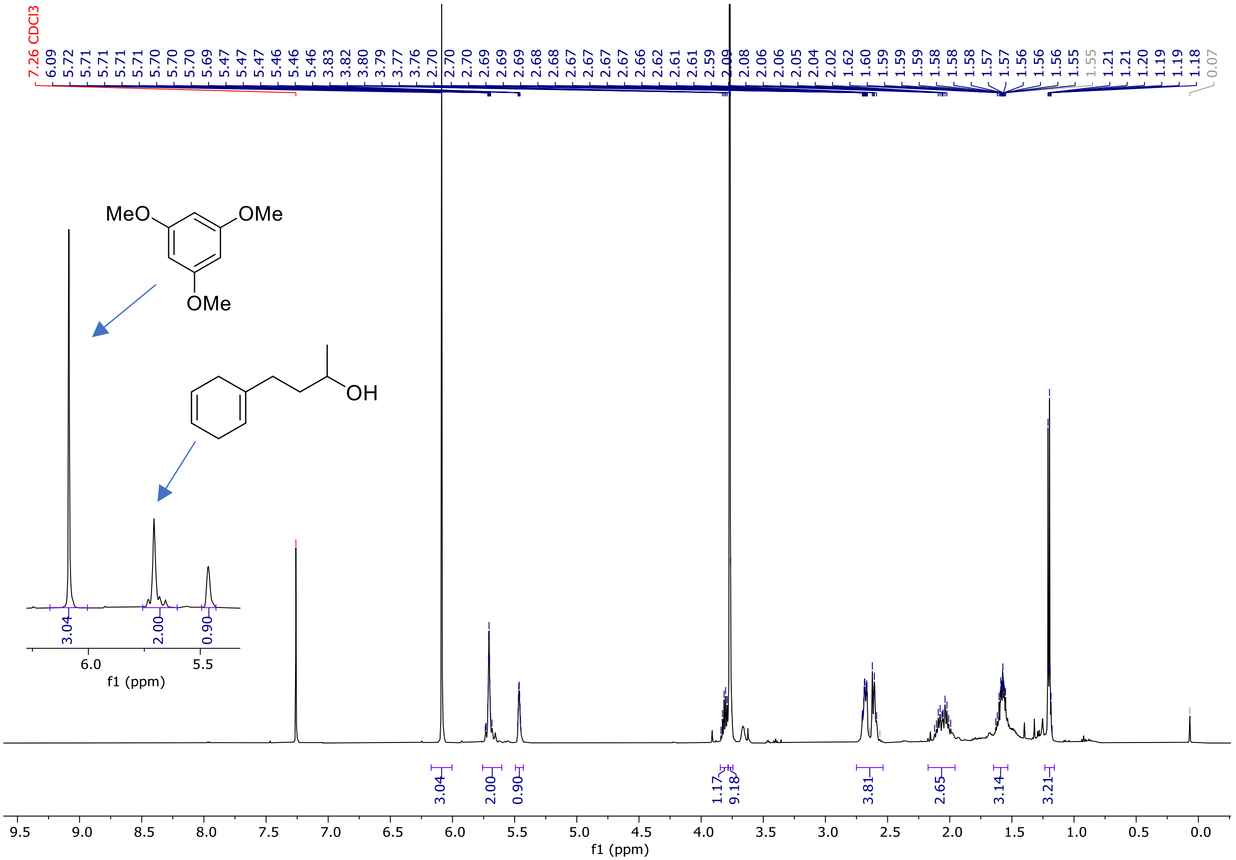

Figure S24. ^{1}H-NMR spectrum for 4-phenylbutan-2-ol reduction using **Method E**. The signals observed at 6.09 and 3.75 ppm were assigned to 1,3,5-TMB and the signals observed at δ 5.76 – 5.60, 5.46, 3.82, 2.75 – 2.54, 2.04, 1.65 – 1.53 and 1.23 – 1.16 ppm were assigned to 4-(cyclohexa-1,4-dien-1-yl)butan-2-ol **3f** which resulted in 32% yield. The spectroscopic data matched those reported in literature.[2]

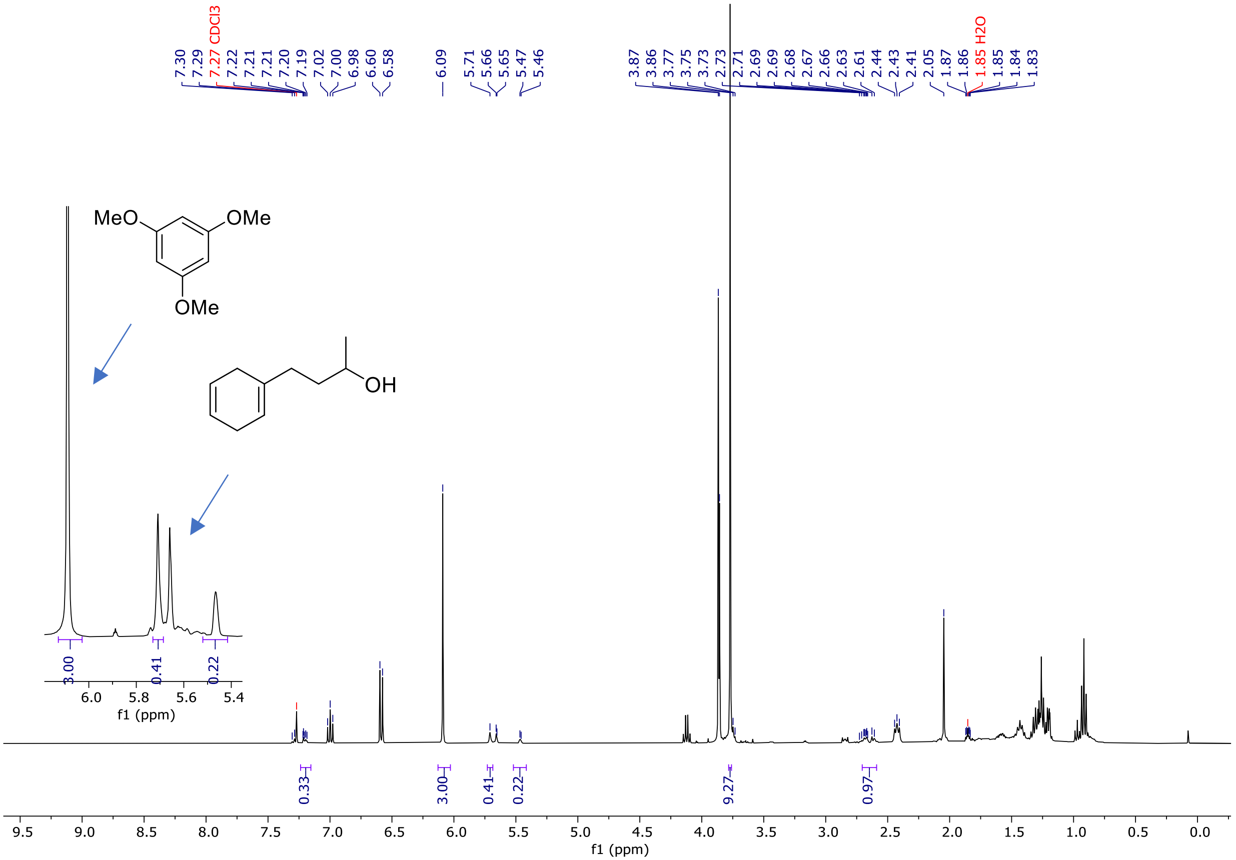

Figure S25. ^{1}H-NMR spectrum for 4-phenylbutan-2-ol reduction using **Method G**. The signals observed at 6.09 and 3.75 ppm were assigned to 1,3,5-TMB and the signals observed at 5.76 – 5.60, 5.46, 3.82, 2.75 – 2.54, 2.04, 1.65 – 1.53 and 1.23 – 1.16 ppm were assigned to 4-(cyclohexa-1,4-dien-1-yl)butan-2-ol **3f** which resulted in 41% yield. The spectroscopic data matched those reported in literature.[2]

S2.2.4 Products of cross-electrophile coupling of alkyl halides

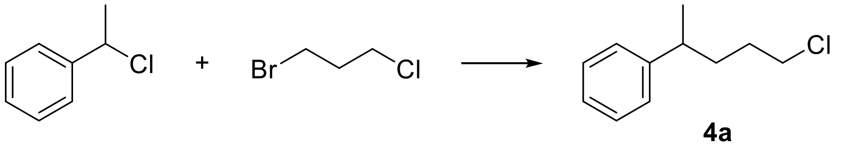

4a

(5-chloropentan-2-yl)benzene (**4a**): Synthesized using **Methods H** or **I** (S2 General procedure) with (1-chloroethyl)benzene (2.0 mmol, 0.28 g), 1-bromo-3-chloropropane (4.0 mmol, 0.63g) and purified by column chromatography (P.E). **4a** was obtained as a clear oil (**H**: 0.25 g, 70% yield; **I**: 0.11 g, 30% yield). ^{1}**H-NMR** (400 MHz, $CDCl_3$) δ 7.29 (m, 2H), 7.20 – 7.09 (m, 3H), 3.46 (m, 2H), 2.86 – 2.53 (m, 1H), 1.97 – 1.39 (m, 4H), 1.25 (d, J = 6.9 Hz, 3H). ^{13}**C-NMR** (101 MHz, $CDCl_3$) δ 146.9, 128.5, 127.0, 126.1, 45.3, 39.5, 35.5, 30.8, 22.5. The data matches that reported in the literature.[4]

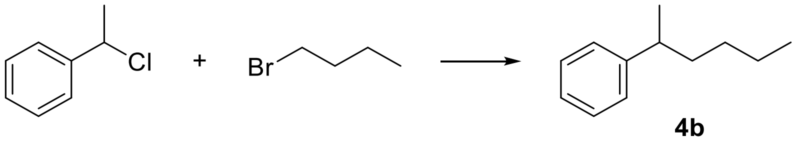

4b

2-phenylhexane (**4b**): Synthesized according to **Methods H** or **I** (S2 General procedure) with (1-chloroethyl)benzene (2.0 mmol, 0.28 g), 1-bromobutane (4.0 mmol, 0.55 g) and purified by column chromatography (P.E). **4b** was obtained as a clear oil (**H**: 0.16 g, 50% yield; **I**: 0.11 g. 35% yield). ^{1}**H-NMR** (400 MHz, $CDCl_3$) δ 7.36 – 7.27 (m, 2H), 7.20 (d, J = 6.9 Hz, 2H), 2.68 (q, J = 7.1 Hz, 1H), 1.68 – 1.48 (m, 2H), 1.40 – 1.13 (m, 7H), 0.86 (t, J = 7.1 Hz, 3H). ^{13}**C-NMR** (101 MHz, $CDCl_3$) δ 148.0, 128.3, 127.0, 125.7, 40.0, 38.2, 30.0, 22.8, 22.4, 14.1. The data matches that reported in the literature.[5]

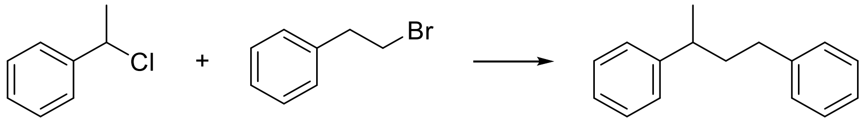

4c

1,3-diphenylbutane (**4c**): Synthesized according to **Methods H** or **I** (S2 General procedure) with (1-chloroethyl)benzene (2.0 mmol, 0.28 g), 1-bromo-3-phenylpropane (4.0 mmol, 0.80 g) and purified by column chromatography (P.E). **4c** was obtained as a clear oil (**H**: 0.33 g, 73% yield; **I**: 0.17 g 38% yield).^{1}**H-NMR** (400 MHz, $CDCl_3$) δ 7.37 – 7.24 (m, 4H), 7.21 – 7.05 (m, 6H), 2.83 – 2.46 (m, 3H), 1.78 – 1.41 (m, 4H), 1.24 (d, J = 6.9 Hz, 3H). ^{13}**C-NMR** (101 MHz, $CDCl_3$) δ 147.61, 142.63, 128.40, 128.32, 128.23, 127.84, 127.63, 127.00, 125.85, 125.62, 39.89, 38.00, 36.00, 29.57, 22.37. The data matches that reported in the literature.[6]

S2.2.5 Products of desulfurative borylation of thioethers

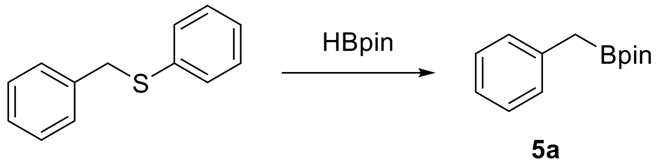

5a

Benzylboronic acid pinacol ester (5a): Synthesis according to **Method J** and **K**. Quantification using HPLC (**Method J** = 93%, and **Method K** = 99% yield).

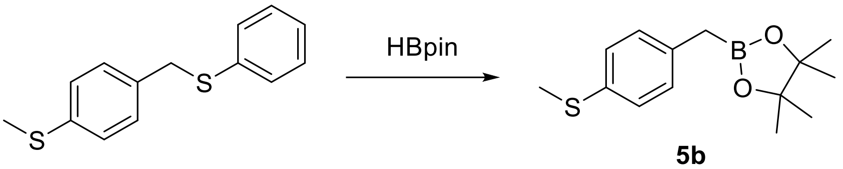

5b

4,4,5,5-tetramethyl-2-(4-(methylthio)benzyl)-1,3,2-dioxaborolane (5b): Synthesized according to **Methods J** and **K** and purified by column chromatography (PE/EtOAc 49:1) obtained **5b** as a clear liquid (**Method J**: 104 mg, 79% yield. **Method K**: 97 mg, 74% yield). **^{1}H NMR** (400 MHz, $CDCl_3$) δ = 7.16 (d, J=8.4, 1H), 7.11 (d, J=8.4, 2H), 2.45 (s, 3H), 2.25 (s, 2H), 1.23 (s, 12H). **^{13}C NMR** (101 MHz, $CDCl_3$) δ = 135.9, 134.0, 129.5, 127.4, 83.5, 24.8, 16.5. **^{11}B NMR** (128 MHz, $CDCl_3$) δ = 33.2. The spectroscopic data matched those reported in the literature.[7]

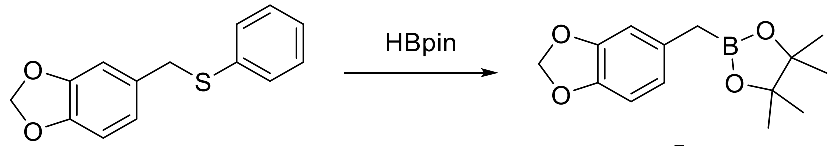

5c

2-(benzo[d][1,3]dioxol-5-ylmethyl)-4,4,5,5-tetramethyl-1,3,2-dioxaborolane (5c) : Synthesized according to **Methods J** and **K** with 3 equivalent pinacolborane. Purified by column chromatography (P.E/EtOAc 49:1), **5c** obtained as a clear liquid (**Method J**: 117 mg, 88% yield. **Method K**: 119 mg, 91% yield). **^{1}H NMR** (400 MHz, $CDCl_3$) 1H NMR (400 MHz, CDCl3) δ = 6.72 – 6.66 (m, 2H), 6.65 – 6.58 (m, 1H), 5.89 (s, 2H), 2.21 (s, 2H), 1.24 (s, 12H). **^{13}C NMR** (101 MHz, $CDCl_3$) δ = 147.5, 145.1, 132.3, 121.6, 109.7, 108.2, 100.7, 83.6, 24.9. **^{11}B NMR** (128 MHz, $CDCl_3$) δ = 33.2. **HRMS (ESI) m/z:** $[M+H]^+$ calculated for $C_{16}H_{26}BF_2O_4$ 263.1455; found 263.1452.

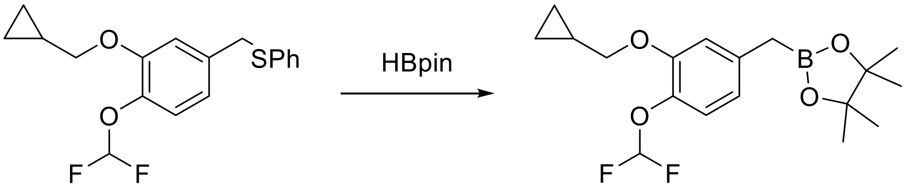

5d

2-(3-(cyclopropylmethoxy)-4-(difluoromethoxy)benzyl)-4,4,5,5-tetramethyl-1,3,2-dioxaborolane (5f): Synthesized according to **Methods J** and **K** and purified by column chromatography (P.E/EtOAc 19:1), **5f** obtained as a clear liquid (**Method J**: 99 mg, 56% yield. **Method K**: 122 mg, 69% yield). **^{1}H**

NMR (400 MHz, $CDCl_3$) δ = 7.0 (d, *J*=8.1, 1H), 6.8 (d, *J*=2.0, 1H), 6.7 (dd, *J*=8.1, 2.0, 1H), 6.6 (t, *J*=76.0, 1H), 3.8 (d, *J*=6.9, 2H), 2.2 (s, 2H), 1.2 (s, 13H), 0.7 – 0.6 (m, 2H), 0.4 – 0.3 (m, 2H). **^{13}C NMR** (101 MHz, $CDCl_3$) δ = 150.2, 138.1 (t, J=3.2), 137.6, 122.6, 121.6, 119.2, 116.7, 115.4, 114.1, 83.7, 73.8, 24.9, 10.3, 3.3. **^{11}B NMR** (128 MHz, $CDCl_3$) δ = 33.1. **^{19}F NMR** (377 MHz, $CDCl_3$) δ = -81.3. **HRMS (ESI) m/z:** $[M+H]^+$ calculated for $C_{16}H_{26}BF_2O_4$ 355.1892; found 355.1894.

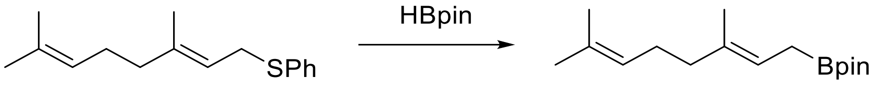

5e

(E)-2-(3,7-dimethylocta-2,6-dien-1-yl)-4,4,5,5-tetramethyl-1,3,2-dioxaborolane (5e): Synthesized according to **Methods J** and **K** and purified by column chromatography (49:1 P.E/EtOAc), **5e** was obtained as a clear liquid (**Method J**: 80 mg, 61% yield. **Method K**: 63 mg, 48% yield). **^{1}H NMR** (400 MHz, $CDCl_3$) δ = 5.26 (tdd, J=6.3, 2.6, 1.3, 1H), 5.12 (tdd, J=5.5, 3.0, 1.5, 1H), 2.12 – 1.97 (m, 4H), 1.69 (d, J=1.4, 3H), 1.65 – 1.58 (m, 8H), 1.26 (s, 12H). **^{13}C NMR** (101 MHz, $CDCl_3$) δ = 135.1, 131.1, 124.5, 118.5, 83.0, 39.8, 26.8, 25.7, 24.8, 17.7, 15.9. **^{11}B NMR** (128 MHz, $CDCl_3$) δ = 33.4. **HRMS (ESI) m/z**: $[M+H]^+$ calculated for $C_{16}H_{30}BO_2$ 265.2339; found 265.2343.

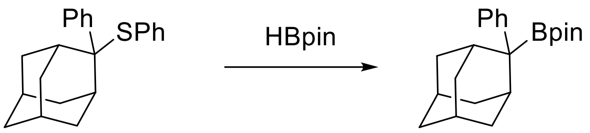

5f

4,4,5,5-tetramethyl-2-((1r,3r,5r,7r)-2-phenyladamantan-2-yl)-1,3,2-dioxaborolane (5d): Synthesized according to **Methods J** and **K** and purified by column chromatography (49:1 P.E/EtOAc), **5d** obtained as a white solid (Method J: 138 mg, 82% yield. Method K: 132 mg, 78% yield). **^{1}H NMR** (400 MHz, $CDCl_3$) δ = 7.42 – 7.38 (m, 2H), 7.31 (ddt, *J*=8.3, 3.6, 2.1, 2H), 7.13 (td, *J*=7.2, 1.7, 1H), 2.74 (s, 2H), 2.04 – 1.95 (m, 5H), 1.89 (d, *J*=12.5, 2H), 1.75 (s, 3H), 1.55 – 1.46 (m, 2H), 1.07 – 1.03 (m, 12H). **^{13}C NMR** (101 MHz, $CDCl_3$) δ = 144.2, 128.1, 127.3, 124.6, 83.0, 38.0, 37.5, 32.1, 31.1, 28.1, 27.5, 24.2. **^{11}B NMR** (128 MHz, $CDCl_3$) δ = 32.5. **HRMS (ESI) m/z**: $[M+H]^+$ calculated for $C_{22}H_{32}BO_2$ 339.2495; found 339.2496.

S3 Electrochemical measurements

General information: Potentiostat instrument CH instruments CHI650E was used for all electrochemical measurements, which were carried out in a single-compartment cell with a three-electrode configuration that consisted of a 1.0 mm glassy carbon working electrode (ALS catalogue No. 002411), Pt-wire counter electrode, and an Ag/$AgNO_3$ reference electrode (with internal solution: 0.1 M NBu_4PF_6 and 0.01 M $AgNO_3$ in MeCN). For determining the oxidative onset potential, the first cycle of each voltammogram was used. The onset potential was quantified through linear interpolation by selecting a region of the oxidative current response which showed linearity between the curvature of the initial oxidative current and the peak oxidative current.

Cyclic voltammetry measurements: Prior to each measurement, the 5-10 mM substrate solution in 0.1 M NBu_4PF_6-THF (5 mL) was purged with argon for a duration of 10 minutes. The measurements were performed using a scan rate of 50-100 mV s^{-1} and were referenced to ferrocenium/ferrocene redox couple ($Fc^{+/0}$) by shifting the V values to the $Fc^{+/0}$ redox couple potential ($E_{1/2}$ = 0.19 V, see Figure S27). After each experiment, the disk electrode was polished using 0.3 µm alumina polishing powder on a microcloth polishing flannel, the platinum wire was flame dried and the reference electrode was washed with THF.

Electroplating of magnesium and zinc metal: Prior to each measurement, 10 mM of the metal salt was dissolved in 0.1 M NBu_4PF_6 in THF (5 mL) by stirring and purging the solution with argon for a duration of 10 minutes. A cylic voltammogram was recorded (see procedure above) to find a suitable reduction potential for the metal. Potentiostatic measurement was performed at the found potential during 1-6 min under constant flow of argon gas. Once finished, a new vial containing 0.1 M 1 M NBu_4PF_6 in THF (5 mL) was purged with argon for 5-10 minutes and a cyclic voltammogram was recorded. After each set of experiments, the disk electrode was polished using 0.3 µm alumina polishing powder on a microcloth polishing flannel, the platinum wire was flame dried and the reference electrode was washed with THF.

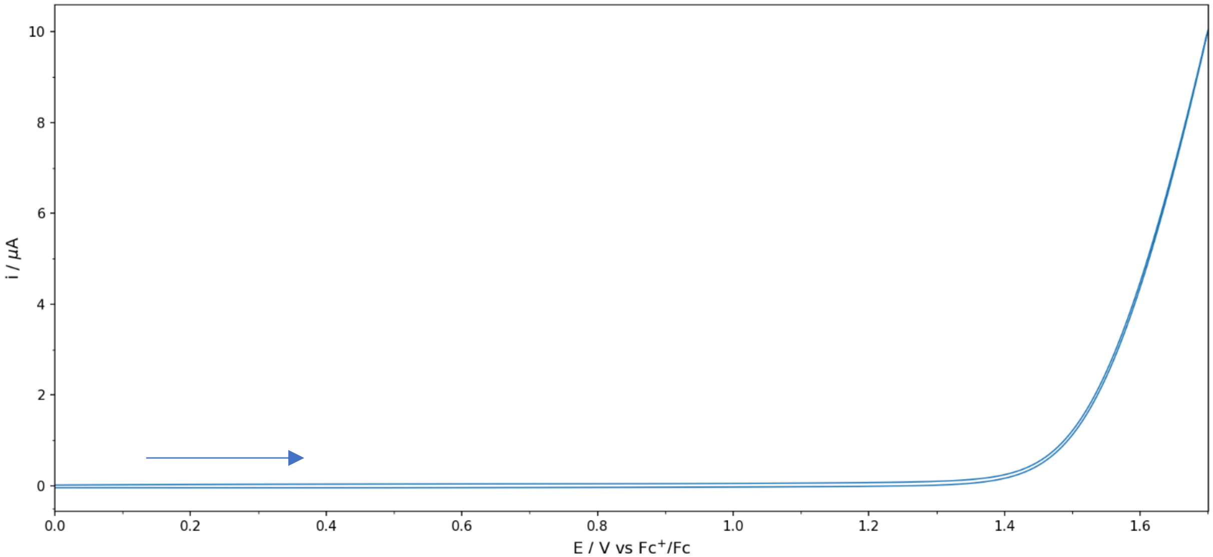

Figure S26. Cyclic voltammogram of 0.1 M NBu_4PF_6 in THF using 100 mVs^{-1} scan rate.

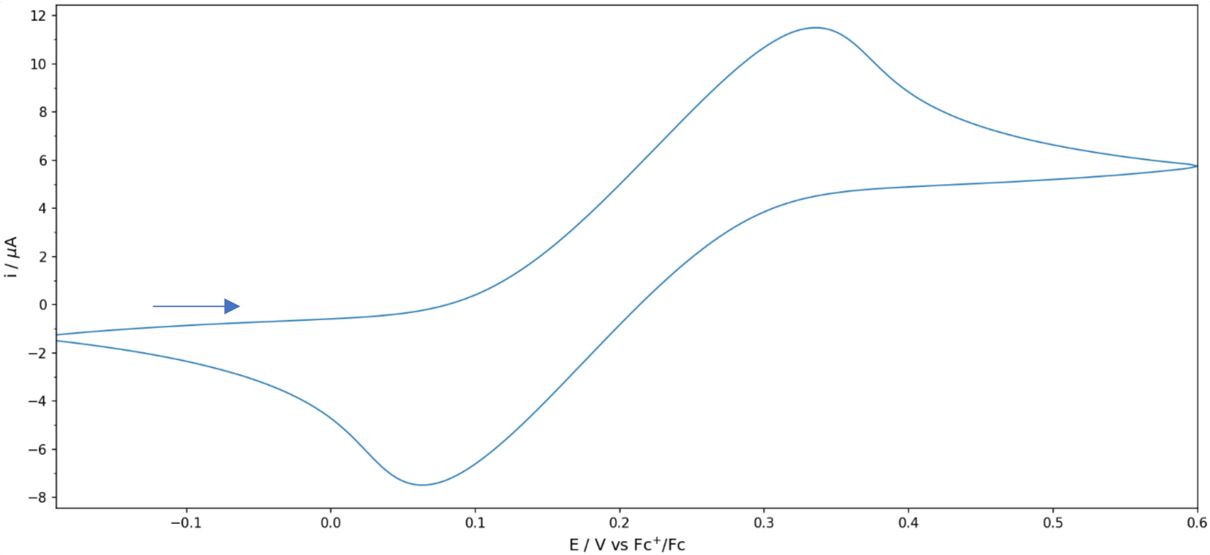

Figure S27. Cyclic voltammogram of 5 mM $Fc^{+/0}$ in THF with 0.1 M NBu_4PF_6 using 100 mVs^{-1} scan rate.

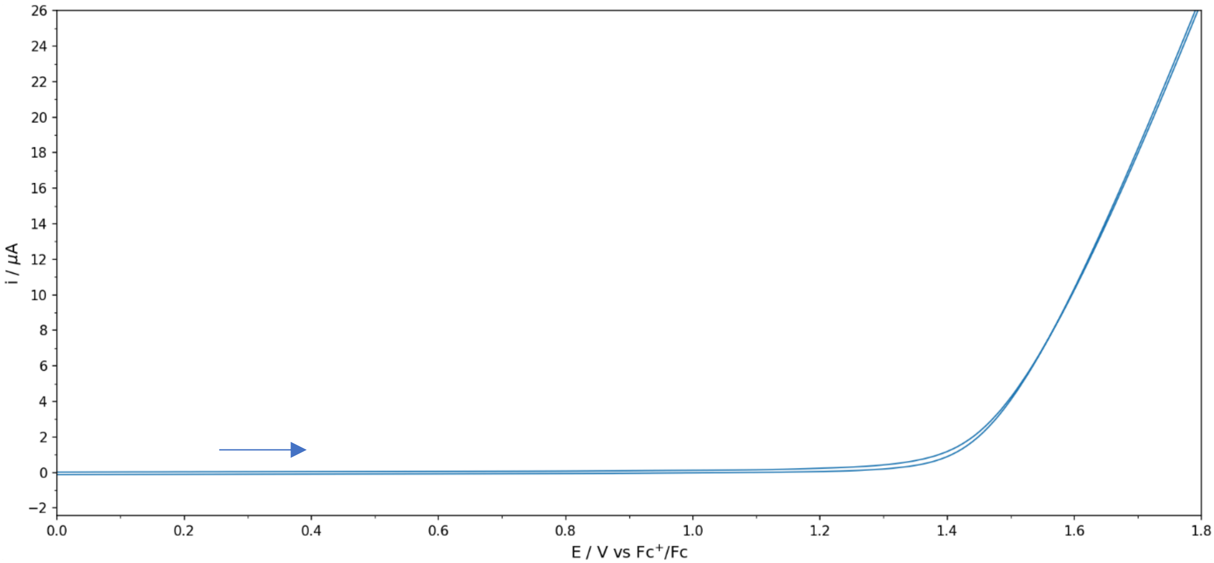

Figure S28. Cyclic voltammogram of 5 mM AcOH in THF with 0.1 M NBu_4PF_6 using 50 mVs^{-1} scan rate.

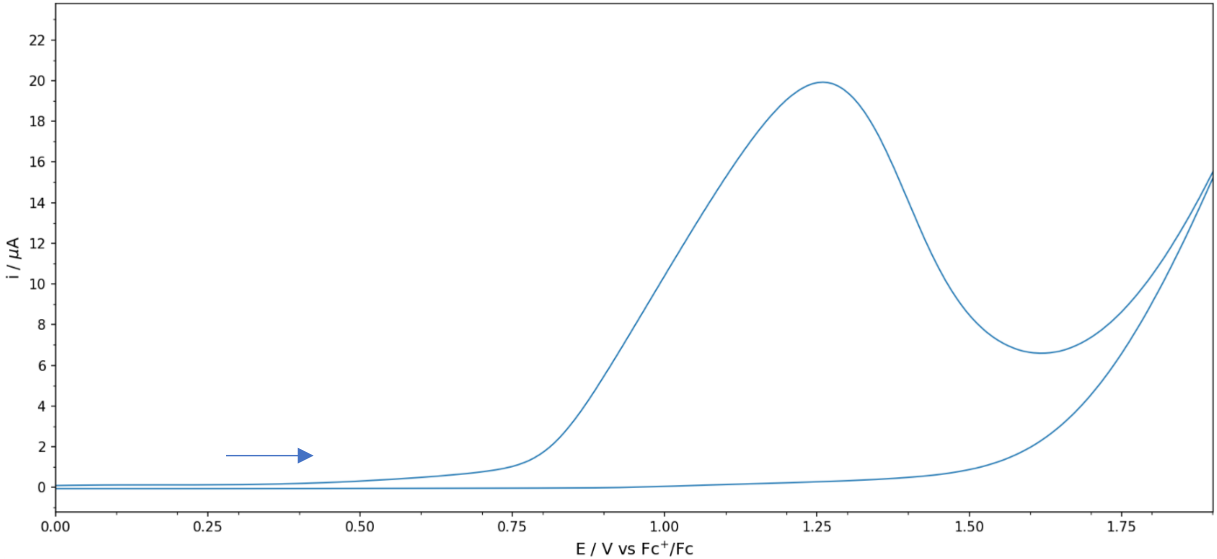

Figure S29. Cyclic voltammogram of 5 mM phenol in THF with 0.1 M NBu_4PF_6 using 100 mVs^{-1} scan rate.

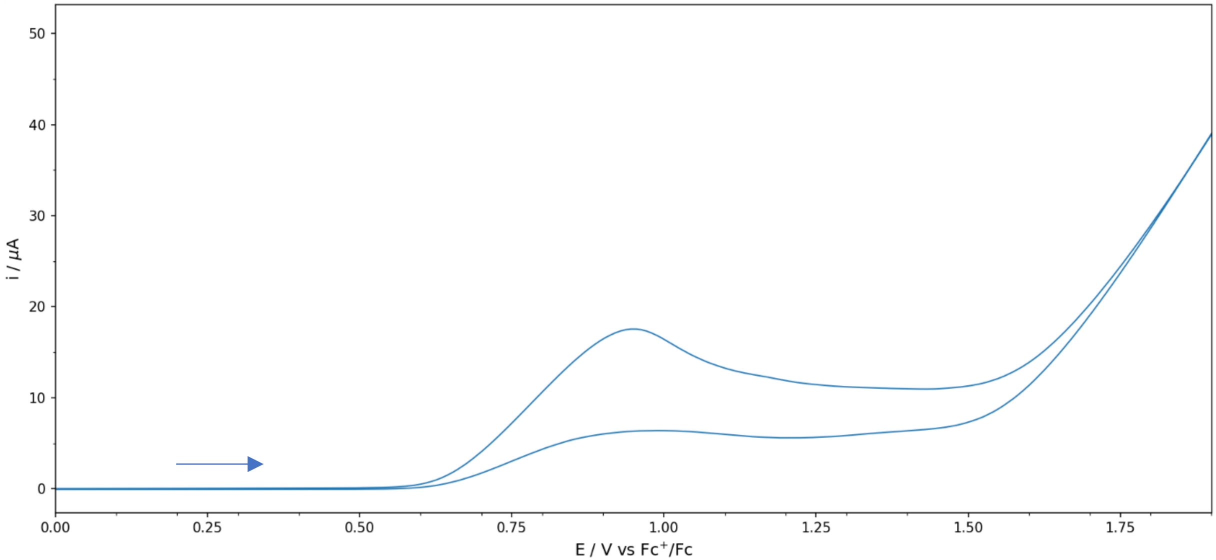

Figure S30. Cyclic voltammogram of 5 mM triphenylphosphine in THF with 0.1 M NBu_4PF_6 using 100 mVs^{-1} scan rate.

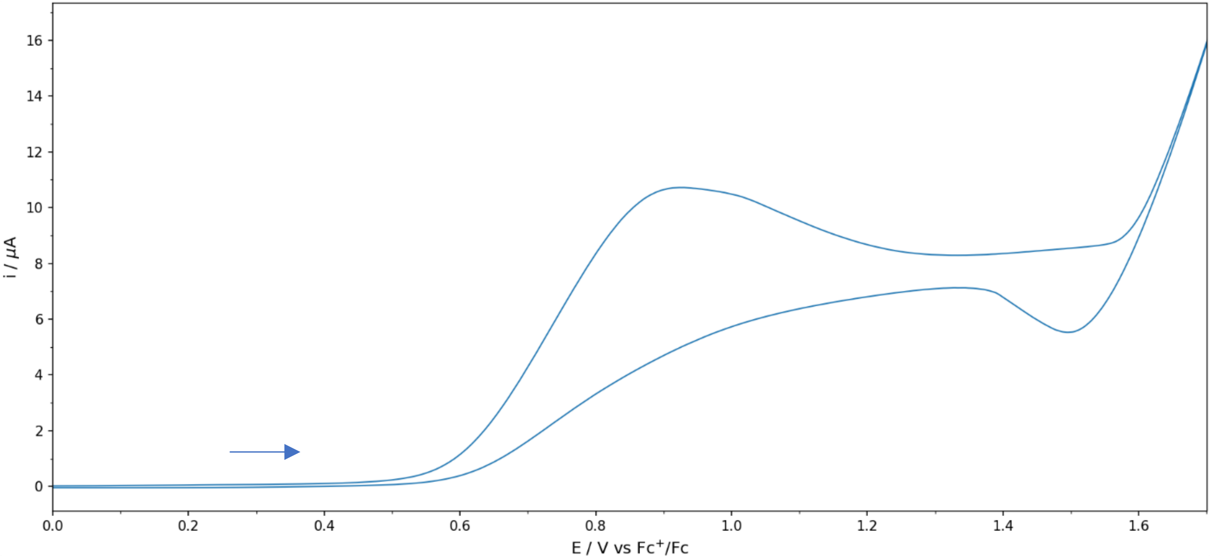

Figure S31. Cyclic voltammogram of 5 mM diisopropylamine in THF with 0.1 M NBu_4PF_6 using 100 mVs^{-1} scan rate.

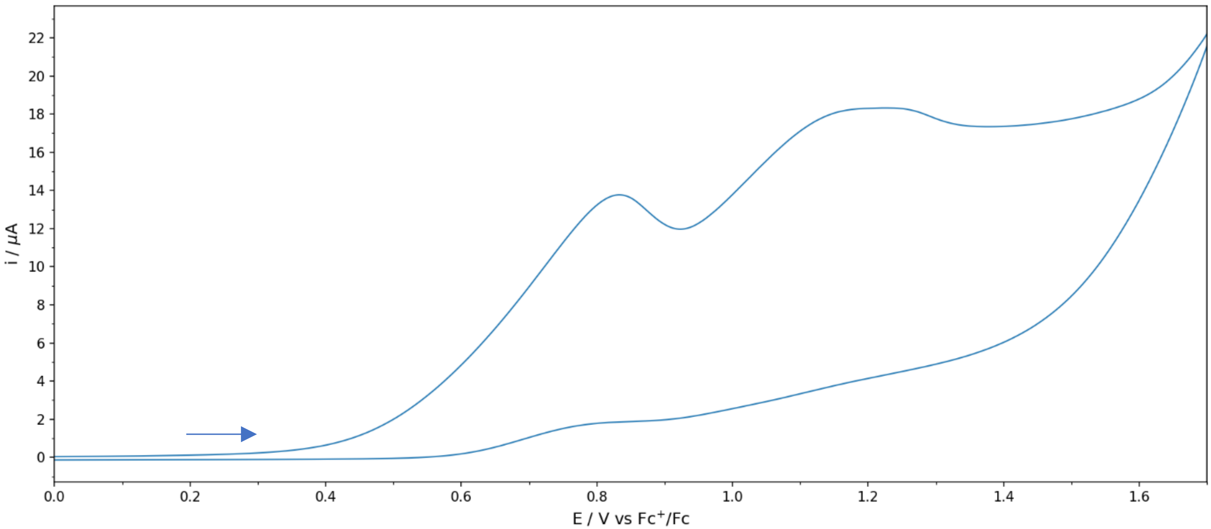

Figure S32. Cyclic voltammogram of 5 mM diphenylthiourea in THF with 0.1 M NBu_4PF_6 using 100 mVs^{-1} scan rate.

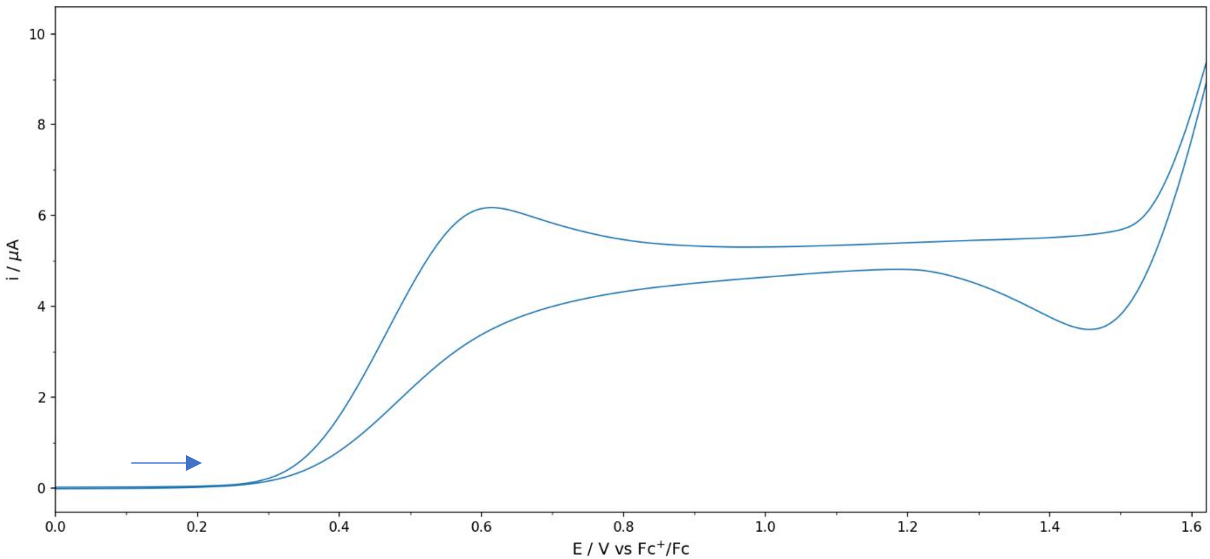

Figure S33. Cyclic voltammogram of 5 mM triethylamine in THF with 0.1 M NBu_4PF_6 using 50 mVs^{-1} scan rate.

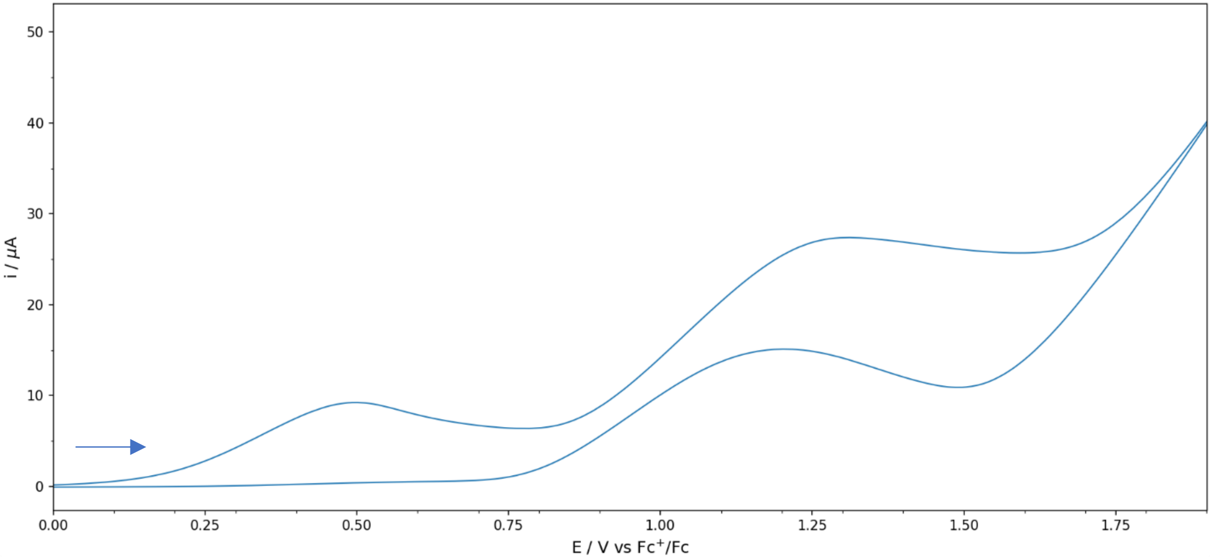

Figure S34. Cyclic voltammogram of 5 mM thiourea in THF with 0.1 M NBu_4PF_6 using 100 mVs^{-1} scan rate.

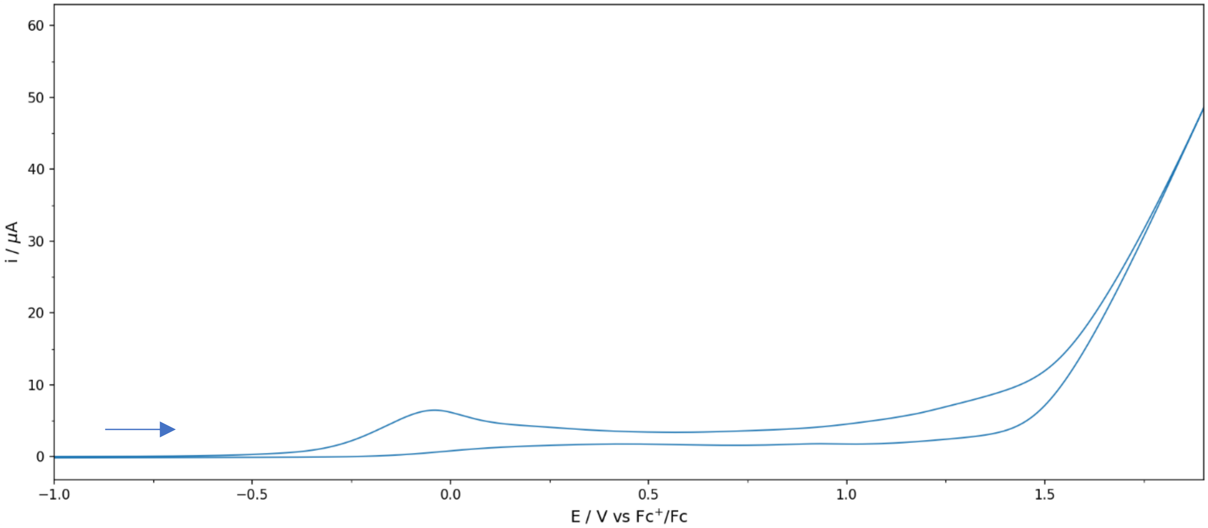

Figure S35. Cyclic voltammogram of 5 mM NBu_4BH_4 in THF with 0.1 M NBu_4PF_6 using 100 mVs^{-1} scan rate.

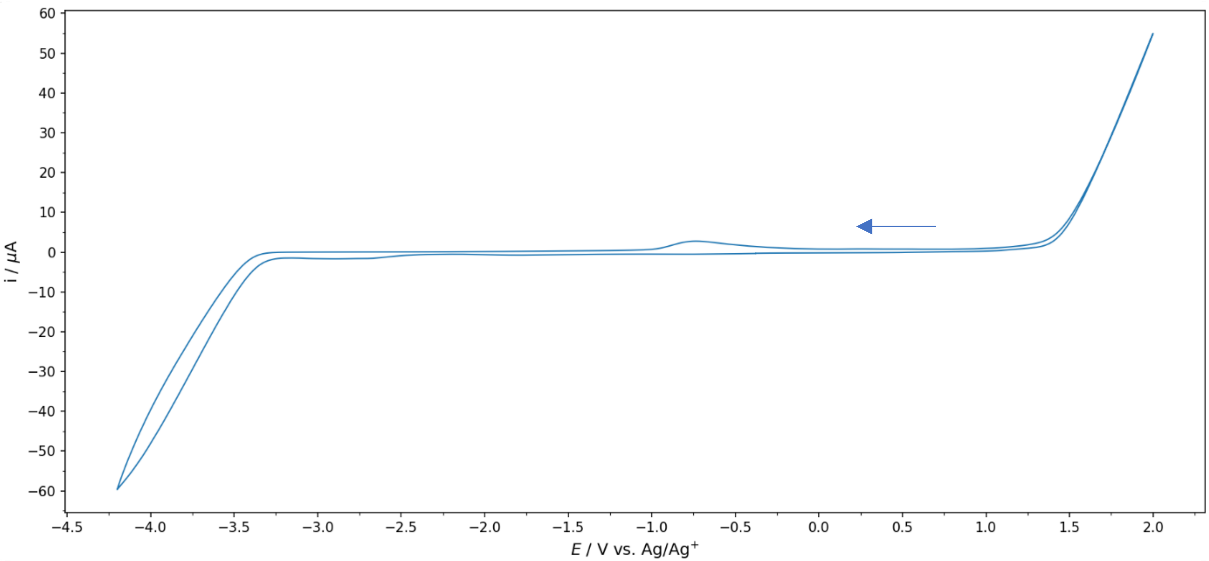

Figure S36. Cyclic voltammogram of 10 mM Mg(II) trifluoromethanesulfonate in THF with 0.1 M NBu_4PF_6 using 50 mVs^{-1} scan rate to investigate the reduction potential of Mg^{2+}.

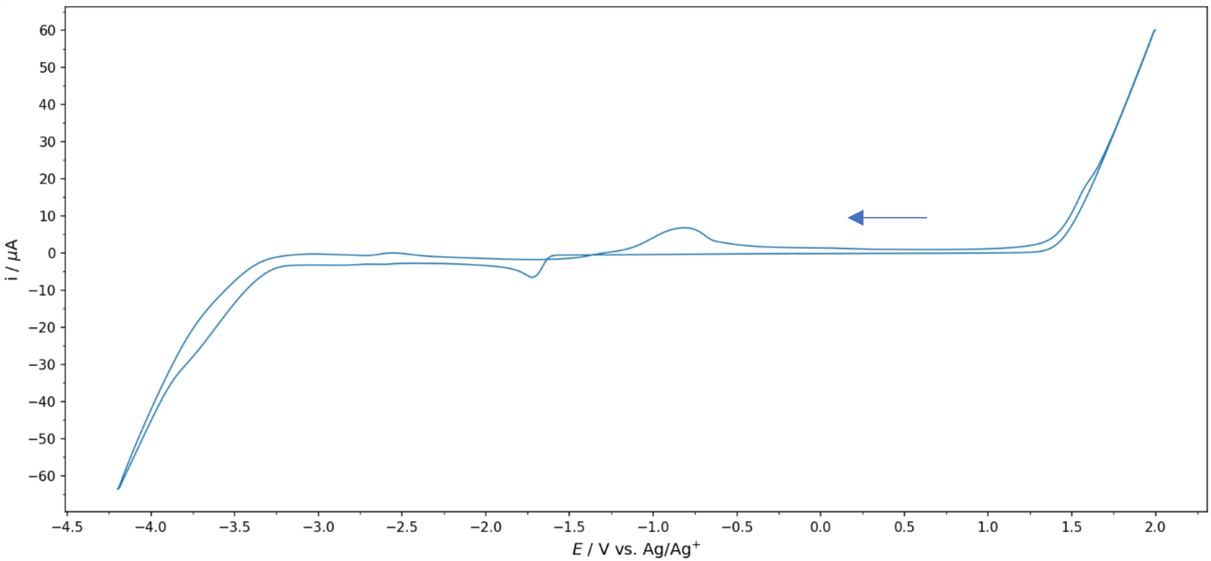

Figure S37. Cyclic voltammogram of 10 mM Zn(II) trifluoromethanesulfonate in THF with 0.1 M NBu_4PF_6 using 50 mVs^{-1} scan rate to investigate the reduction potential of Zn^{2+}.

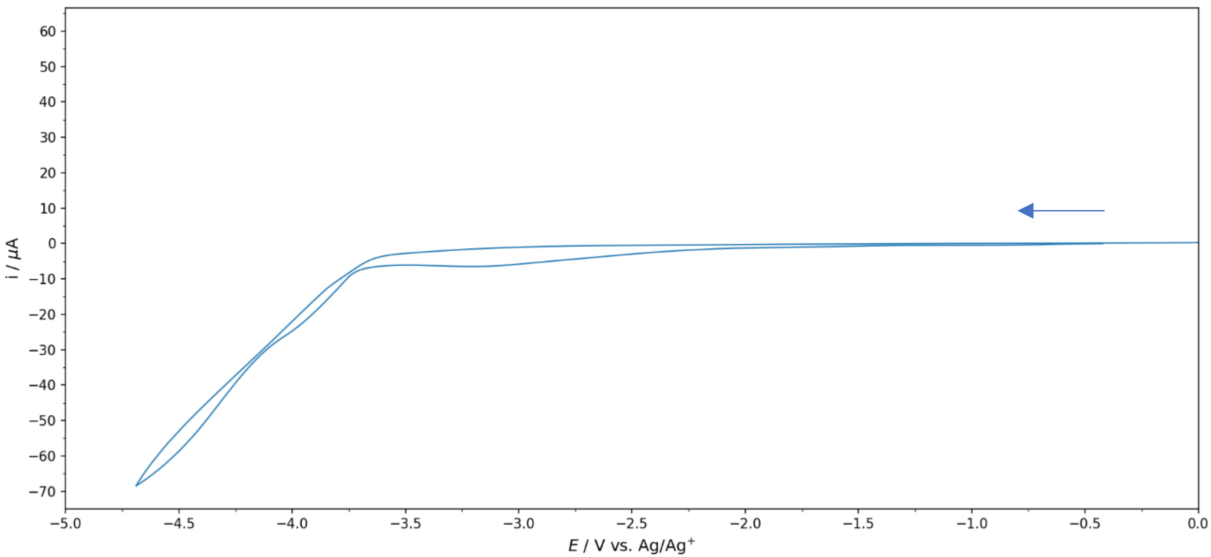

Figure S38. Cyclic voltammograms of Mg(II) perchlorate in THF with 0.1 M NBu_4PF_6 using 50 mVs^{-1} scan rate to investigate the reduction potential of Mg^{2+}.

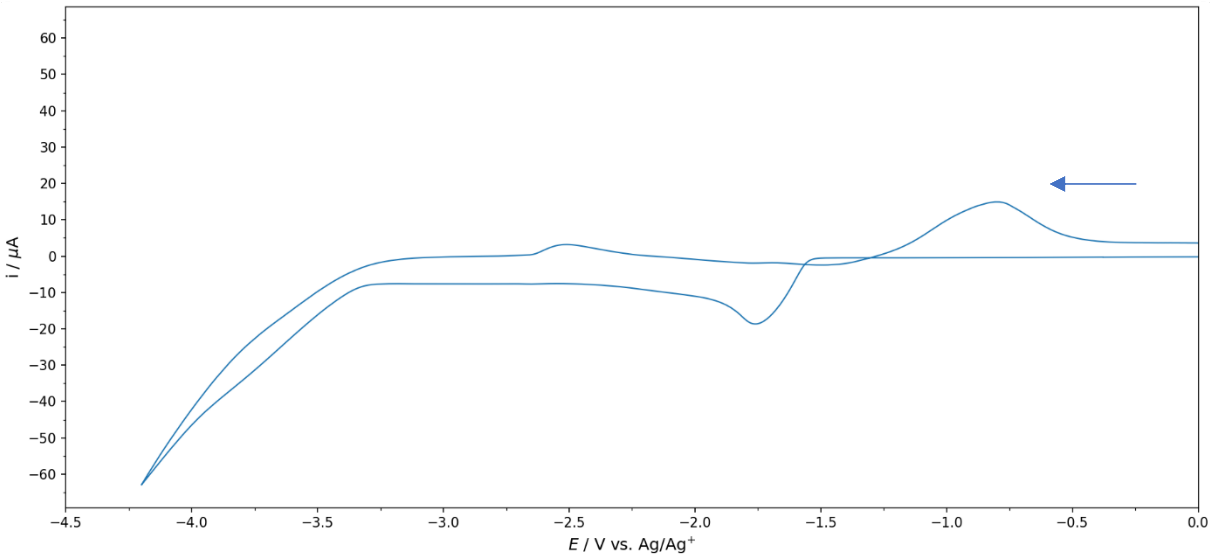

Figure S39. Cyclic voltammogram of 10 mM Zn(II) perchlorate in THF with 0.1 M NBu_4PF_6 using 50 mVs^{-1} scan rate to investigate the reduction potential of Zn^{2+}.

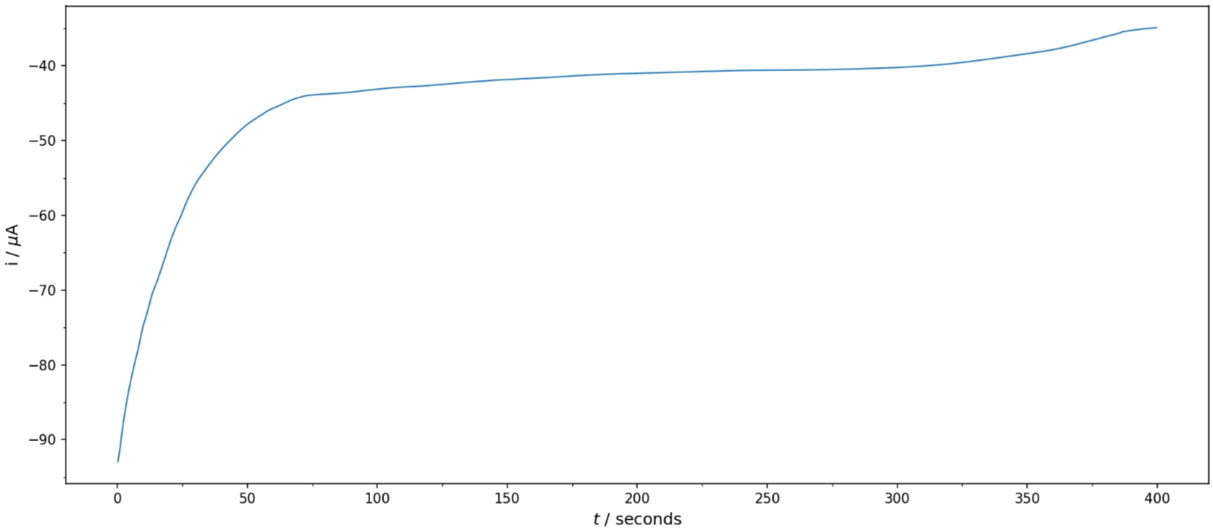

Figure S40. Amperometric i-t curve for electroplating of Mg (from Mg(II) perchlorate) on glassy carbon electrode (WE) at -4.5 V vs Ag/$AgNO_3$ in THF with 0.1 M NBu_4PF_6.

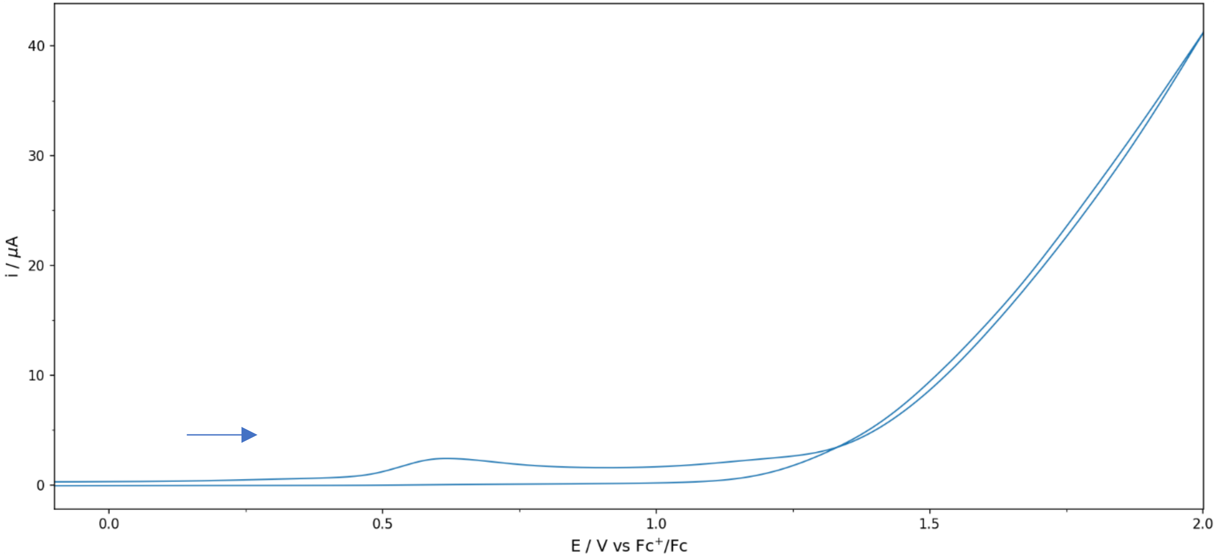

Figure S41. Cyclic voltammogram recorded after electroplating Mg on glassy carbon electrode (WE) with 10 mM Mg(II) perchlorate at -4.5 V vs Ag/$AgNO_3$. The CV was recorded with the Mg-plated glassy carbon electrode (WE), in a new vial containing THF with 0.1 M NBu_4PF_6 using 50 mVs^{-1} scan rate.

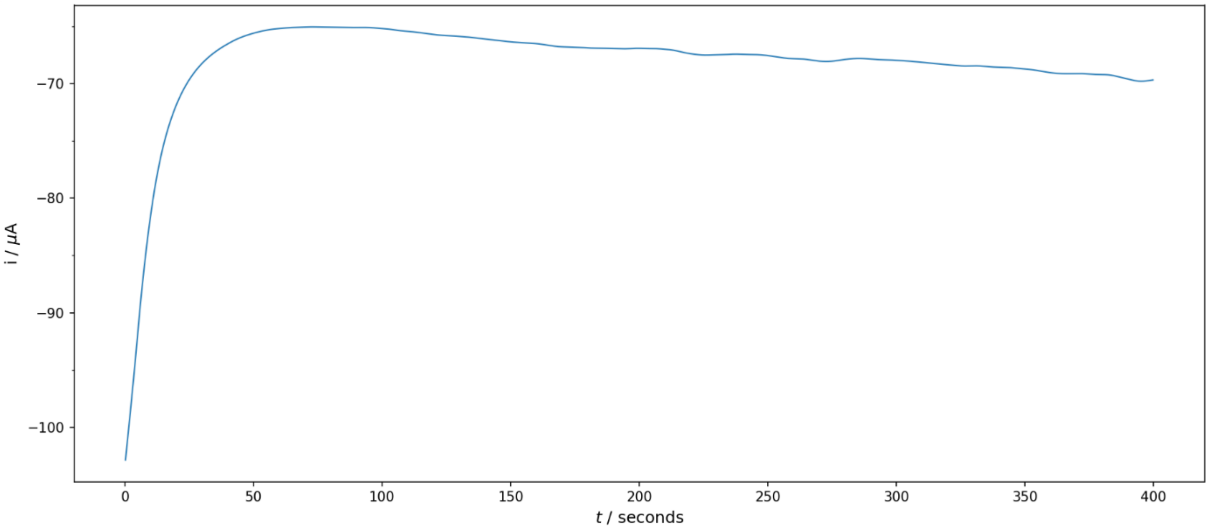

Figure S42. Amperometric i-t curve for electroplating of Mg (from Mg(II) trifluoromethanesulfonate) on glassy carbon electrode (WE) at -4.5 V vs $Ag/AgNO_3$ in THF with 0.1 M NBu_4PF_6.

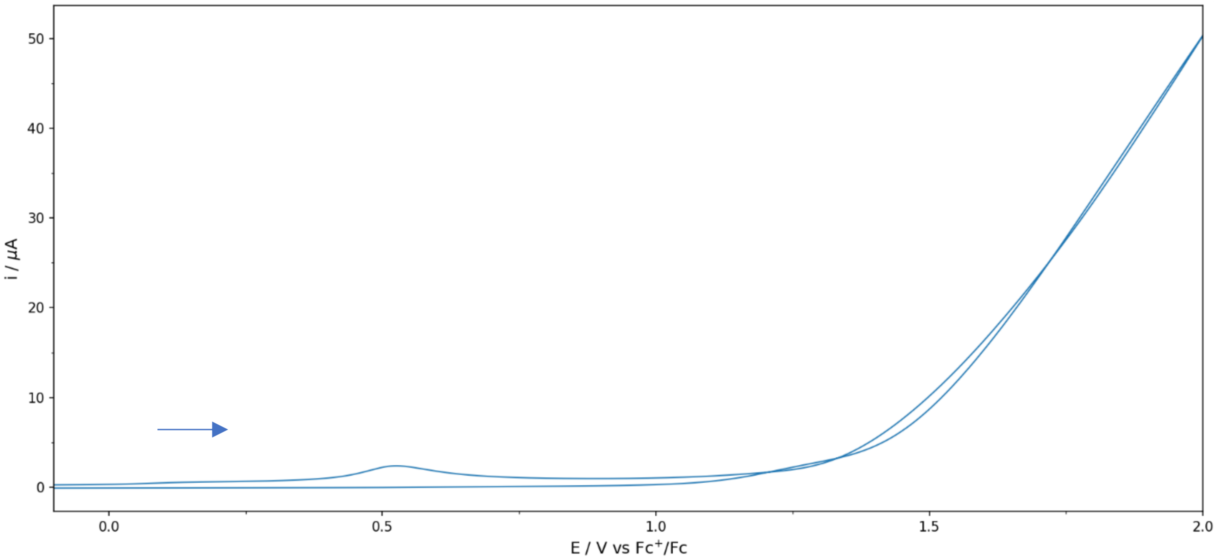

Figure S43. Cyclic voltammogram recorded after electroplating Mg on glassy carbon electrode (WE) with 10 mM Mg(II) trifluoromethanesulfonate at -4.5 V vs Ag/$AgNO_3$. The CV was recorded with the Mg-plated glassy carbon electrode (WE), in a new vial containing THF with 0.1 M NBu_4PF_6 using 50 mVs^{-1} scan rate.

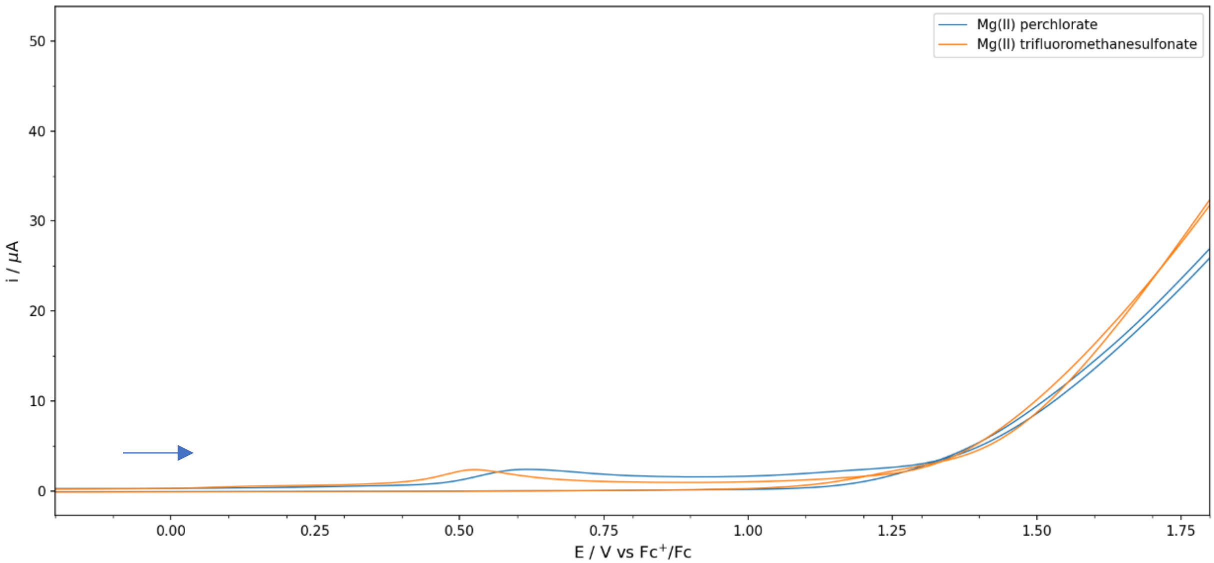

Figure S44. Overlap of cyclic voltammograms recorded after electroplating two different Mg(II) salts on glassy carbon electrode (WE) at -4.5 V vs Ag/$AgNO_3$, respectively. The CVs were recorded with the Mg-plated glassy carbon electrode (WE), in a new vial containing THF with 0.1 M NBu_4PF_6 using 50 mVs^{-1} scan rate.

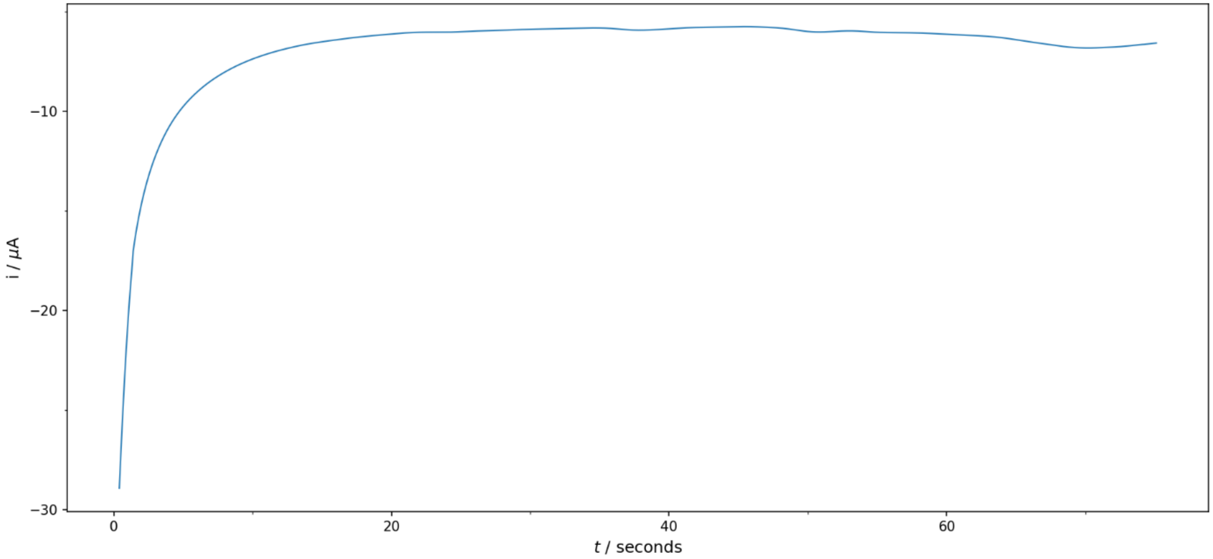

Figure S45. Amperometric i-t curve for electroplating of Zn (from Zn(II) perchlorate) on glassy carbon electrode (WE) at -1.7 V vs Ag/$AgNO_3$ in THF with 0.1 M NBu_4PF_6.

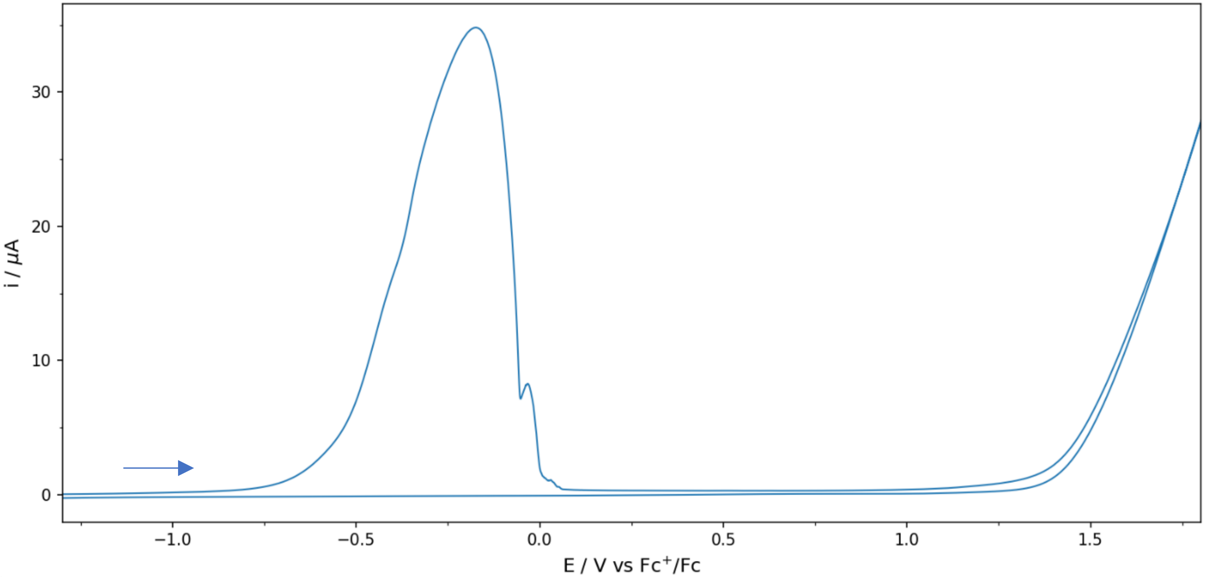

Figure S46. Cyclic voltammogram recorded after electroplating Zn on glassy carbon electrode (WE) with 10 mM Zn(II) perchlorate at -1.7 V vs Ag/$AgNO_3$. The CV was recorded with the Zn-plated glassy carbon electrode (WE), in a new vial containing THF with 0.1 M NBu_4PF_6 using 50 mVs^{-1} scan rate.

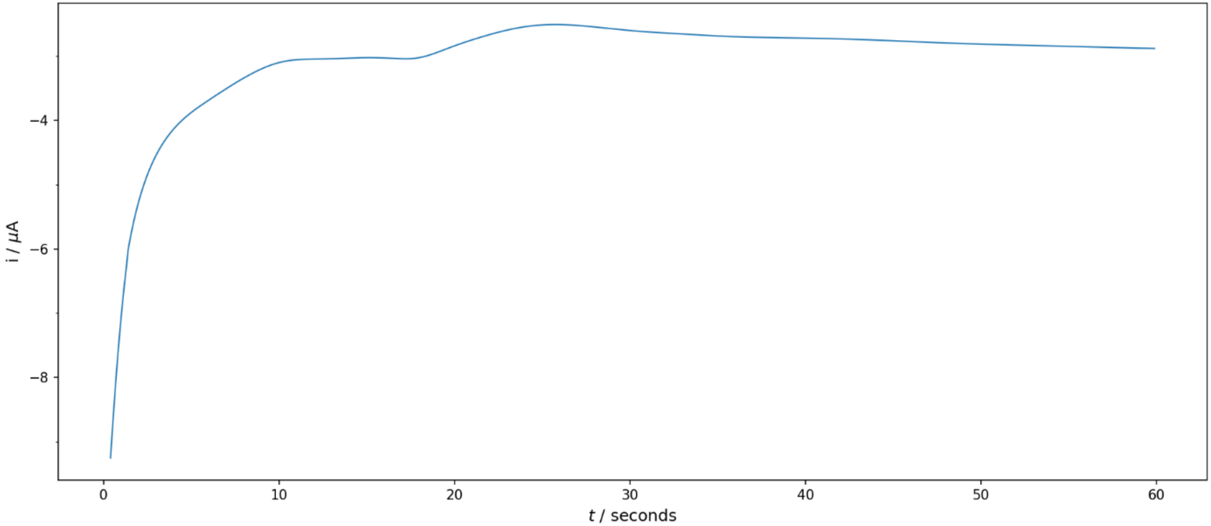

Figure S47. Amperometric i-t curve for electroplating of Zn (from Zn(II) trifluoromethanesulfonate) on glassy carbon electrode (WE) at -1.7 V vs Ag/$AgNO_3$ in THF with 0.1 M NBu_4PF_6.

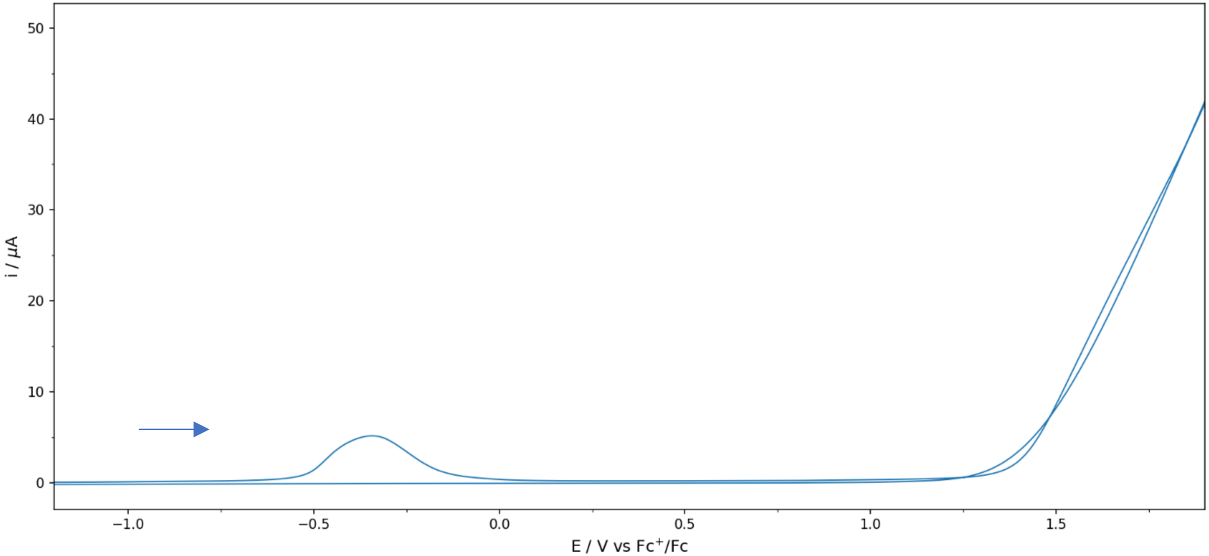

Figure S48. Cyclic voltammogram recorded after electroplating Zn on glassy carbon electrode (WE) with 10 mM Zn(II) trifluoromethanesulfonate at -1.7 V vs Ag/$AgNO_3$. The CV was recorded with the Zn-plated glassy carbon electrode (WE), in a new vial containing THF with 0.1 M NBu_4PF_6 using 50 mVs^{-1} scan rate.

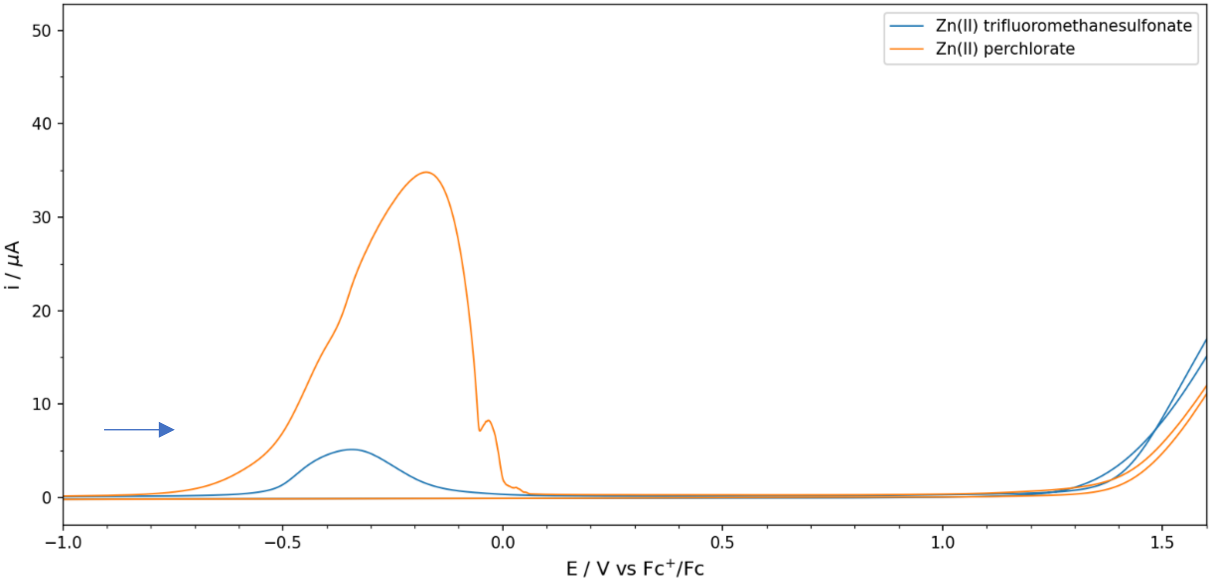

Figure S49. Overlap of cyclic voltammograms recorded after electroplating two different Zn(II) salts on glassy carbon electrode (WE) at -1.7 V vs Ag/$AgNO_3$, respectively. The CVs were recorded with the Zn-coated glassy carbon electrode (WE), in a new vial containing THF with 0.1 M NBu_4PF_6 using 50 mVs^{-1} scan rate.

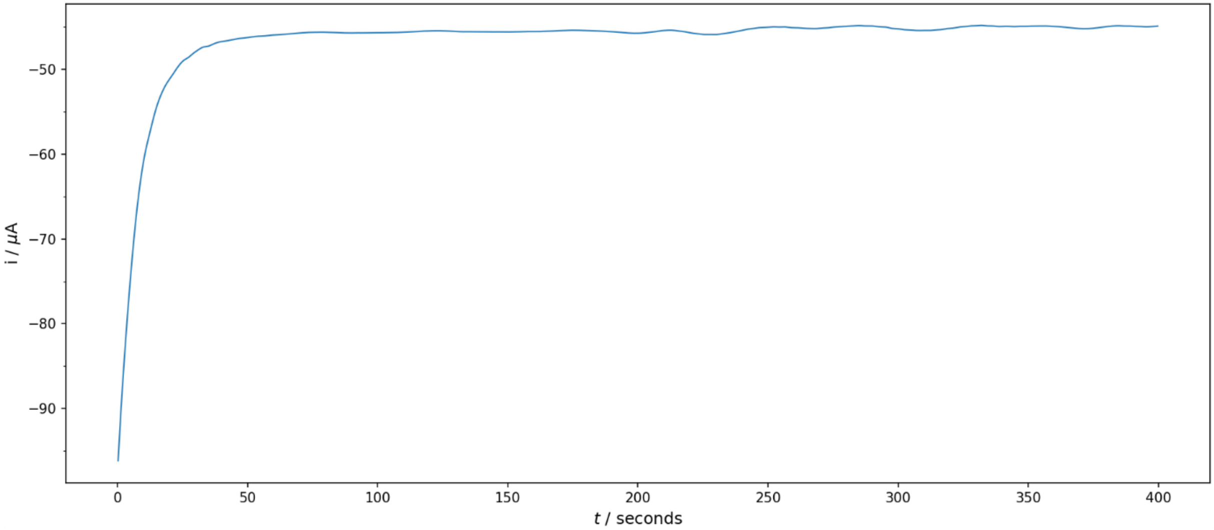

Figure S50. Potentiostatic measurement at -4.5 V in THF with 0.1 M NBu_4PF_6.

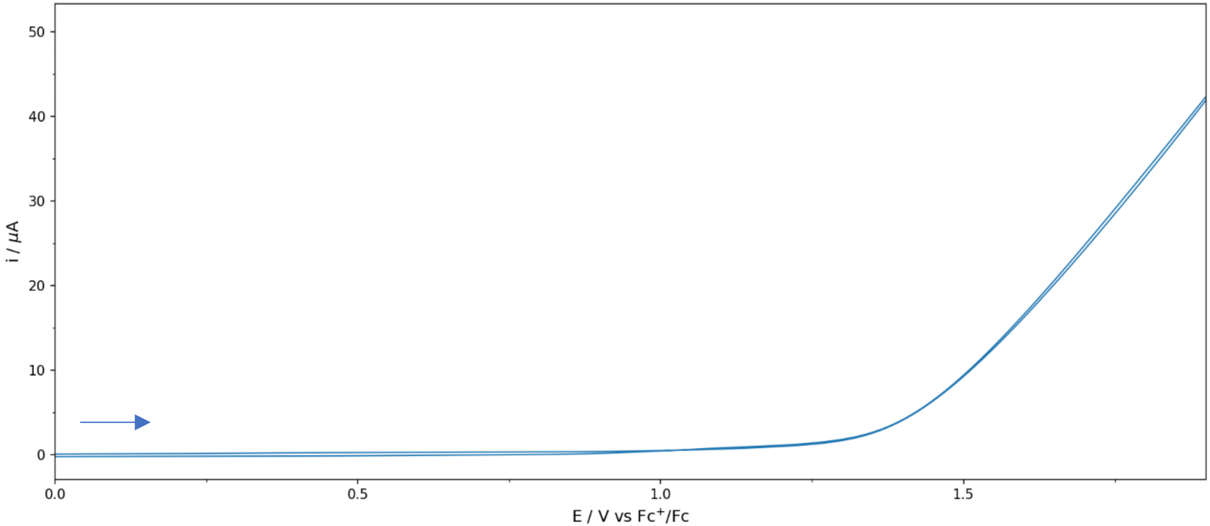

Figure S51. Cyclic voltammogram recorded after amperometry of THF with 0.1 M NBu_4PF_6 on a glassy carbon electrode (WE) at -4.5 V vs Ag/$AgNO_3$. The CV was recorded with the same glassy carbon electrode (WE), in a new vial containing THF with 0.1 M NBu_4PF_6 using 50 mVs^{-1} scan rate.

References

[1] J. Kuzmin, H. Lundberg, **2024**, DOI 10.26434/chemrxiv-2024-9r9hl.

[2] B. K. Peters, K. X. Rodriguez, S. H. Reisberg, S. B. Beil, D. P. Hickey, Y. Kawamata, M. Collins, J. Starr, L. Chen, S. Udyavara, K. Klunder, T. J. Gorey, S. L. Anderson, M. Neurock, S. D. Minteer, P. S. Baran, *Science* **2019**, *363*, 838–845.

[3] J. Kuzmin, J. Röckl, N. Schwarz, J. Djossou, G. Ahumada, M. Ahlquist, H. Lundberg, *Angew. Chem. Int. Ed.* **2023**, *62*, e202304272.

[4] S. Kolb, D. B. Werz, *Chem. – Eur. J.* **2023**, *29*, e202300849.

[5] S. Kail, G. Hilt, *Synlett* **2024**, *35*, 1011–1014.

[6] B. C. Figula, D. L. Kane, K. Balaraman, C. Wolf, *Org. Lett.* **2022**, *24*, 8719–8723.

[7] W. Guan, Y. Chang, S. Lin, *J. Am. Chem. Soc.* **2023**, *145*, 16966–16972.

[8] R. J. Perkins, D. J. Pedro, E. C. Hansen, *Org. Lett.* **2017**, *19*, 3755–3758.

[9] L. Lu, J. C. Siu, Y. Lai, S. Lin, *J. Am. Chem. Soc.* **2020**, *142*, 21272–21278.

S4 NMR data

S4.1 Hydrodesulfurization of thioethers ^{1}H- and ^{13}C-NMR spectrum

^{1}H NMR (500 MHz, $CDCl_3$)

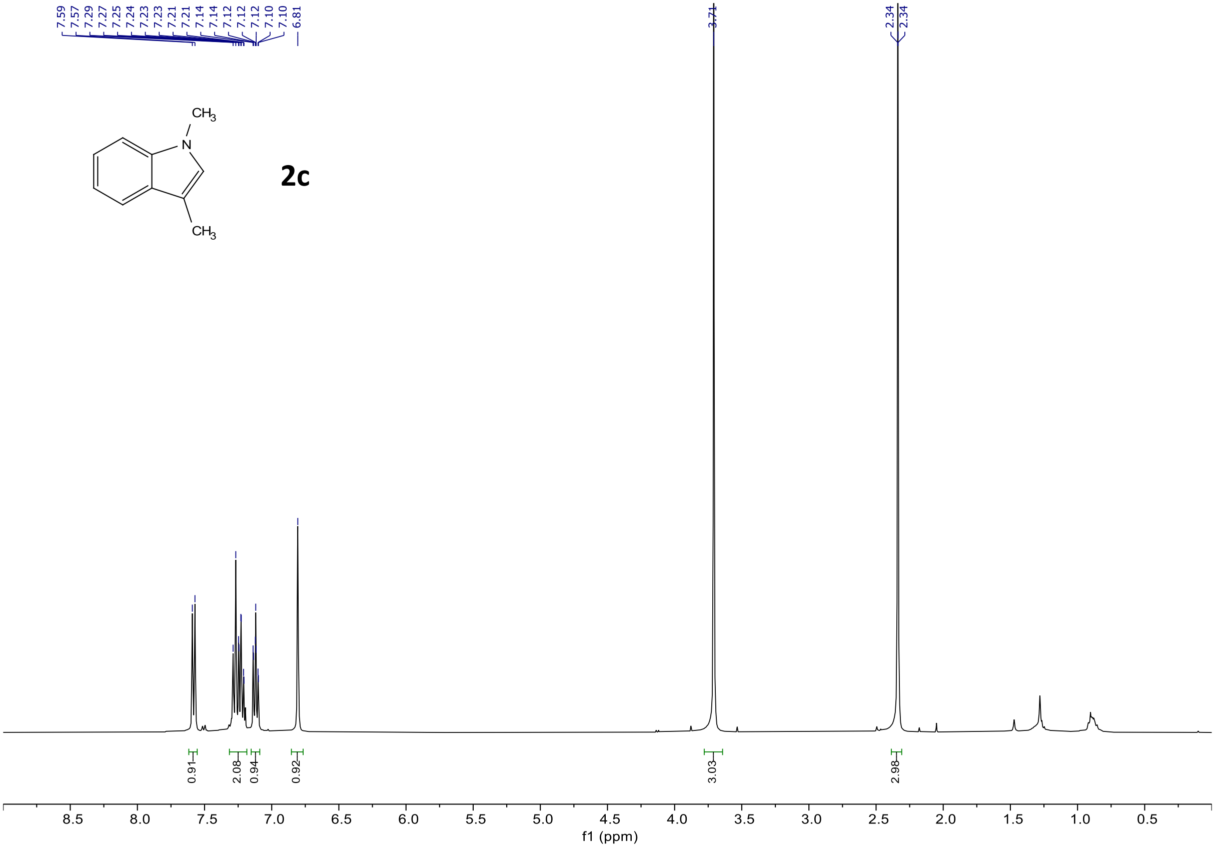

2c

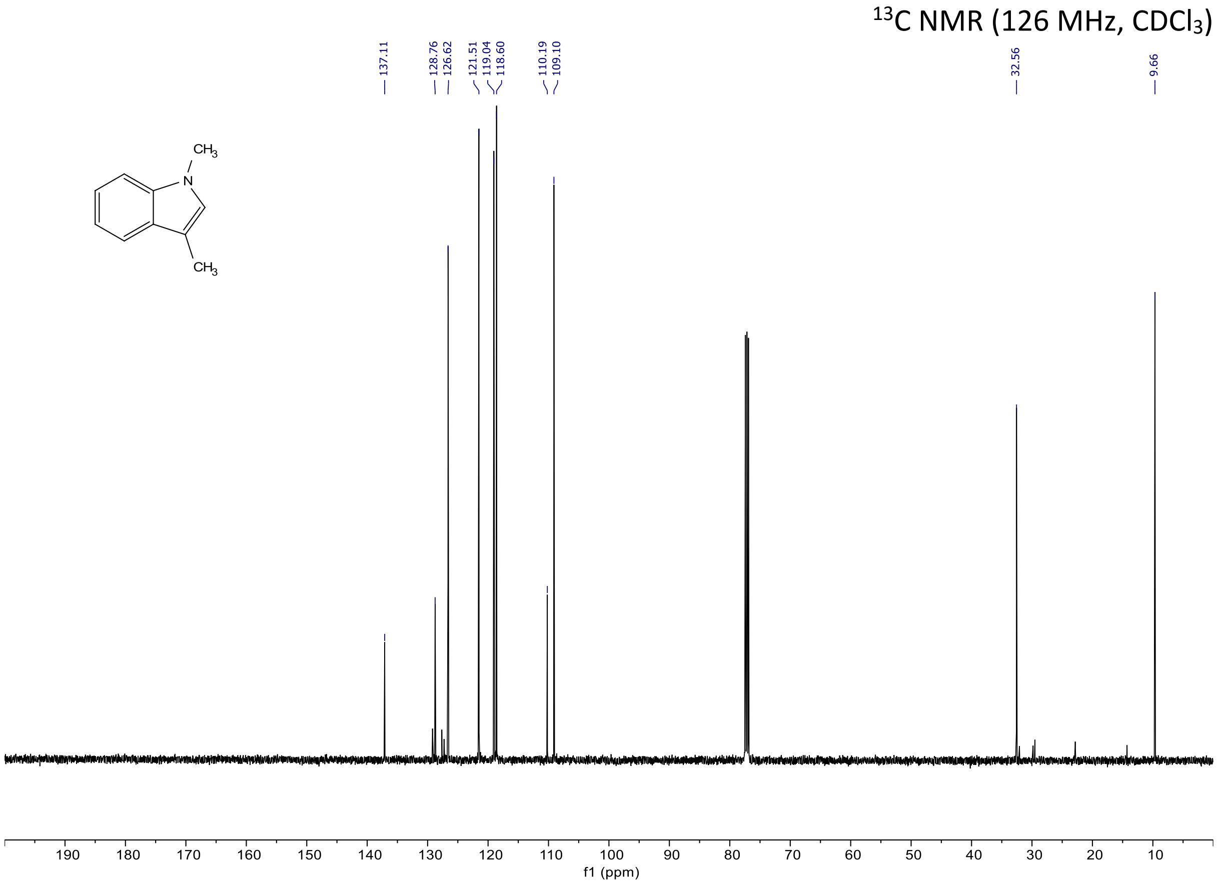
^{13}C NMR (126 MHz, $CDCl_3$)
137.11
128.76
126.62
121.51
119.04
118.60
110.19
109.10
32.56
9.66
CH3
N
CH3
190
180
170
160
150
140
130
120
110
100
90
80
70
60
50
40
30
20
10
f1 (ppm)

S4.2 Cross-electrophile coupling ^{1}H- and ^{13}C-NMR spectrum

S4.3 4a

^{1}H NMR (500 MHz, $CDCl_3$)

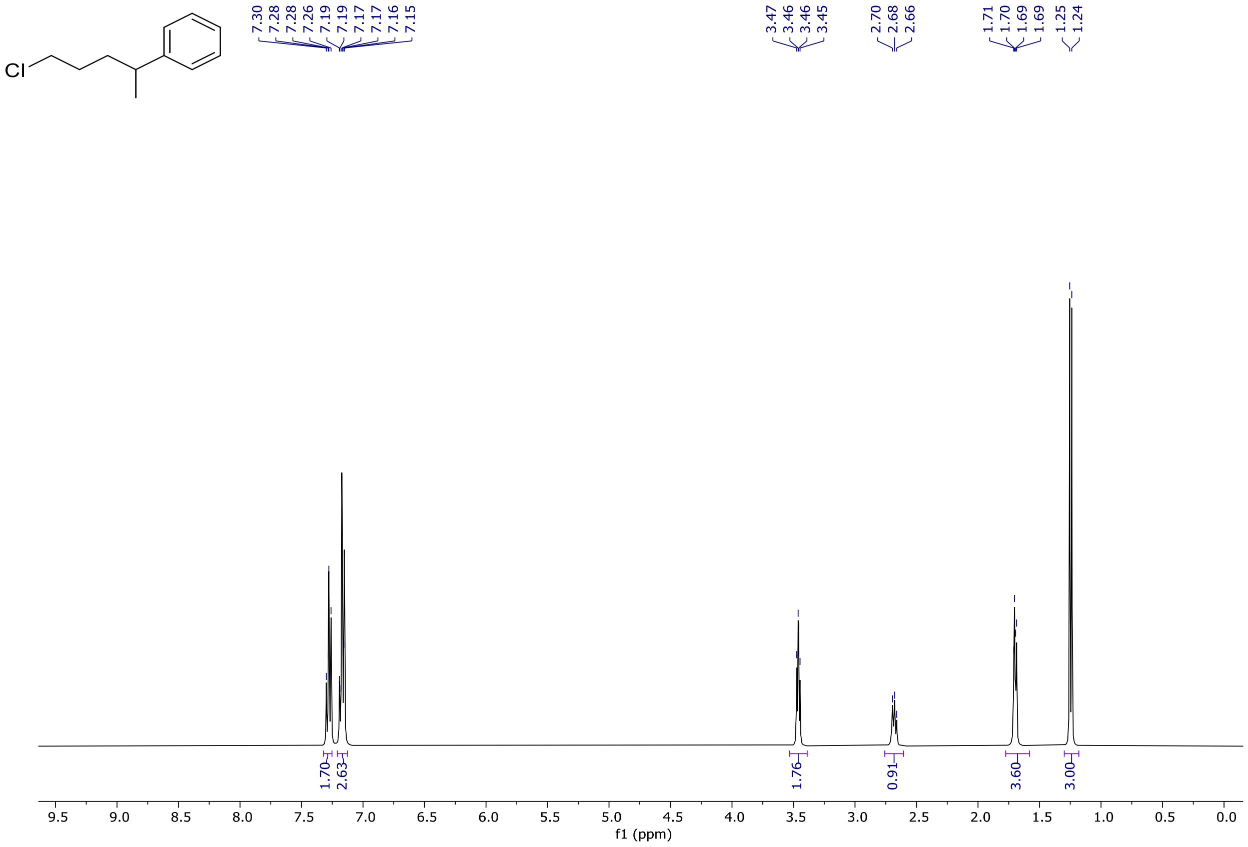

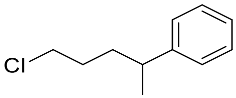

^{13}C NMR (126 MHz, $CDCl_3$)

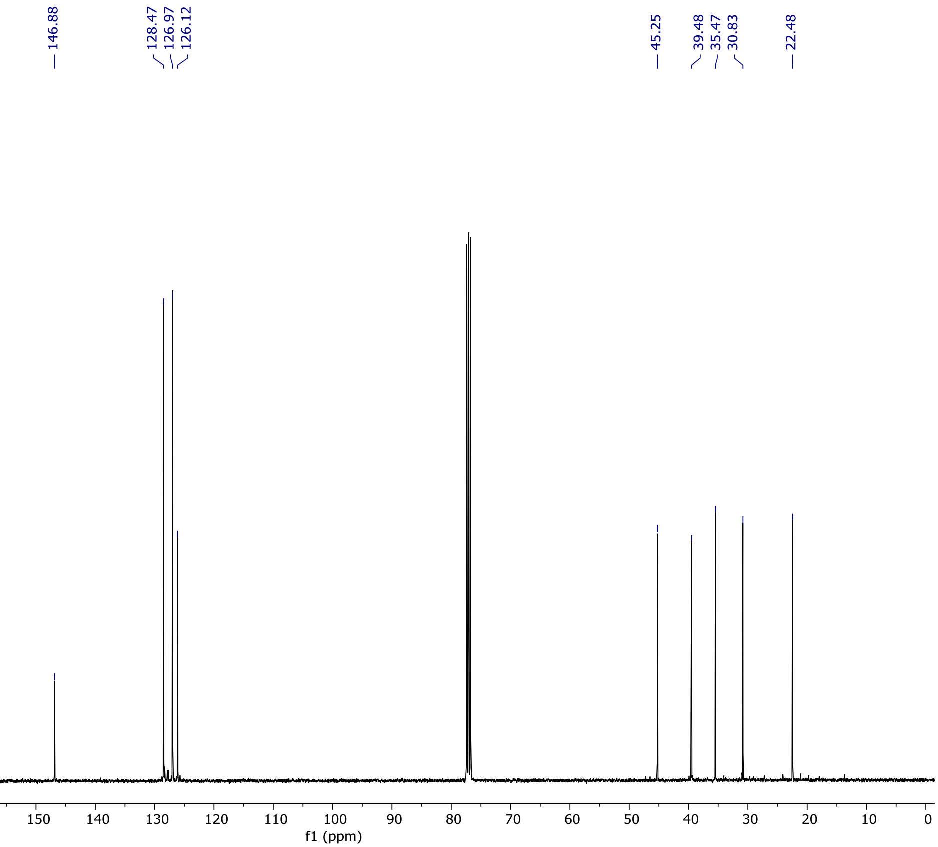

S4.4 4b

^{1}H NMR (500 MHz, $CDCl_3$)

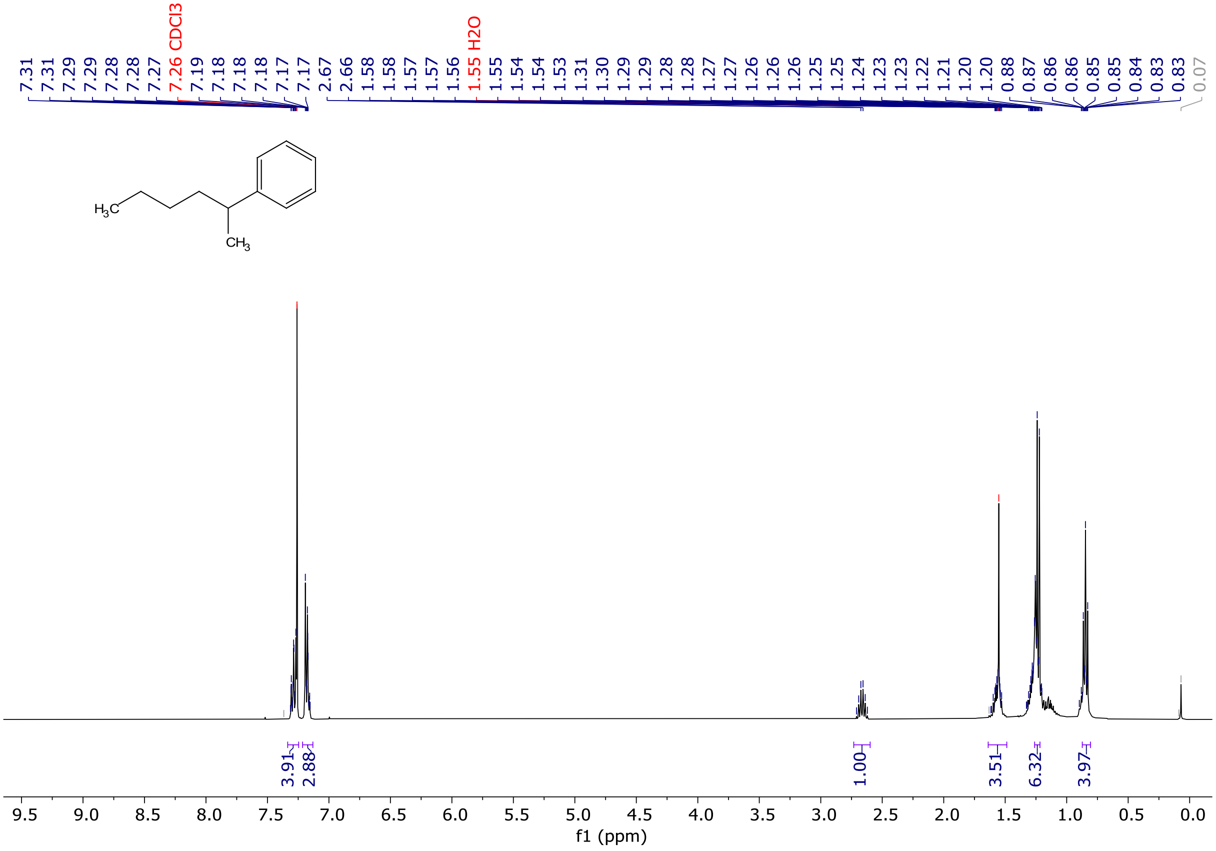

^{13}C NMR (126 MHz, $CDCl_3$)

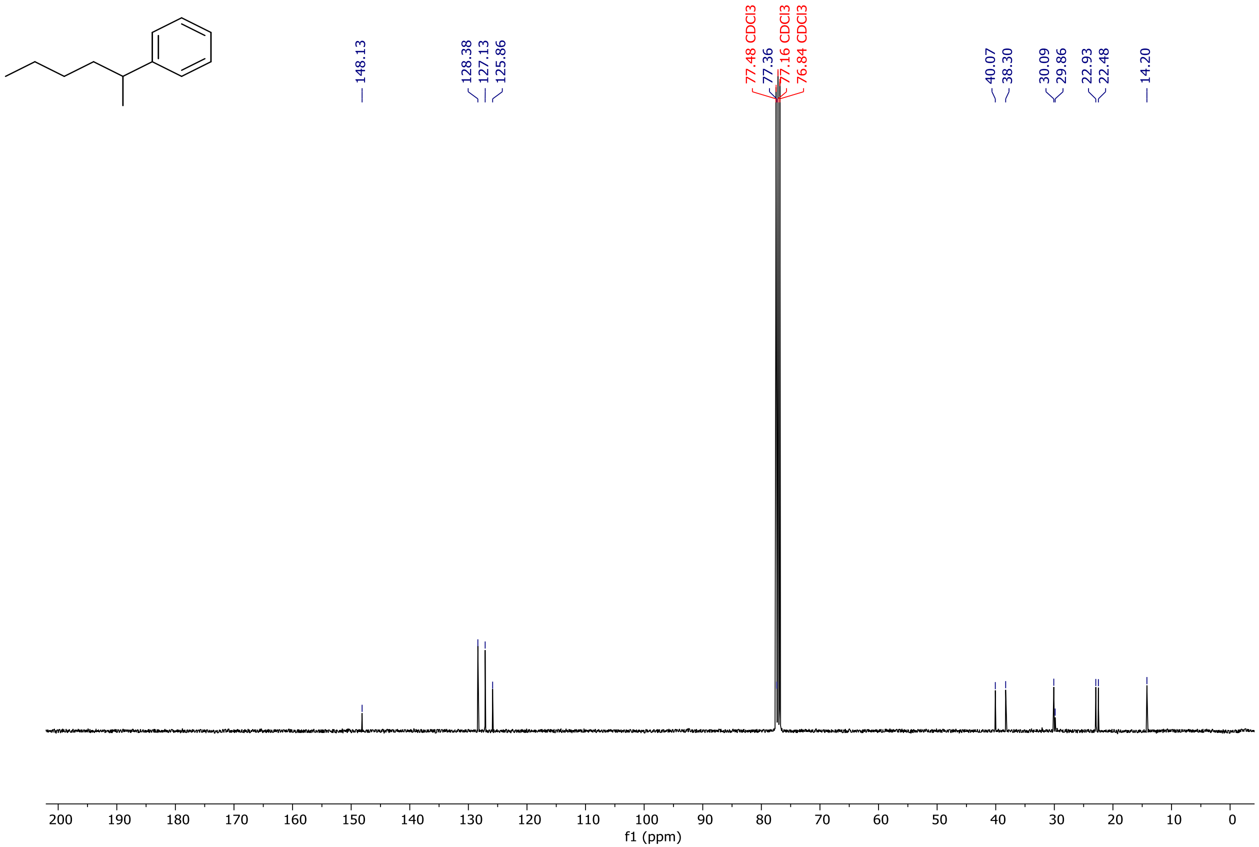

S4.5 4c

^{1}H NMR (500 MHz, $CDCl_3$)

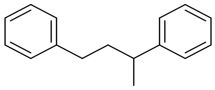

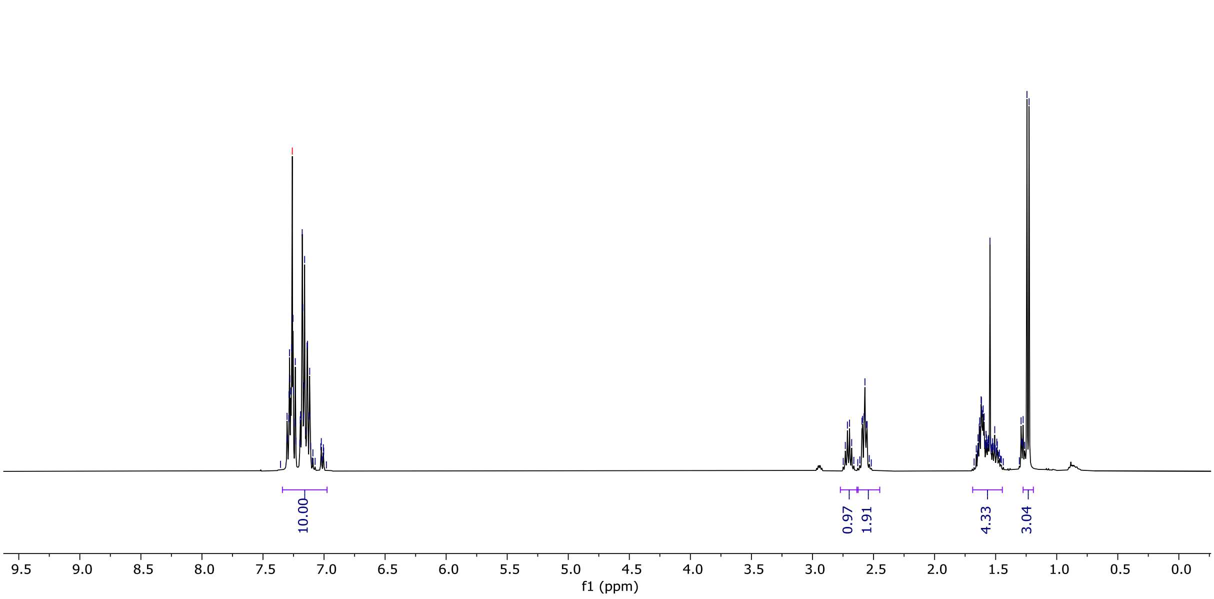

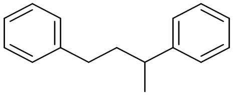

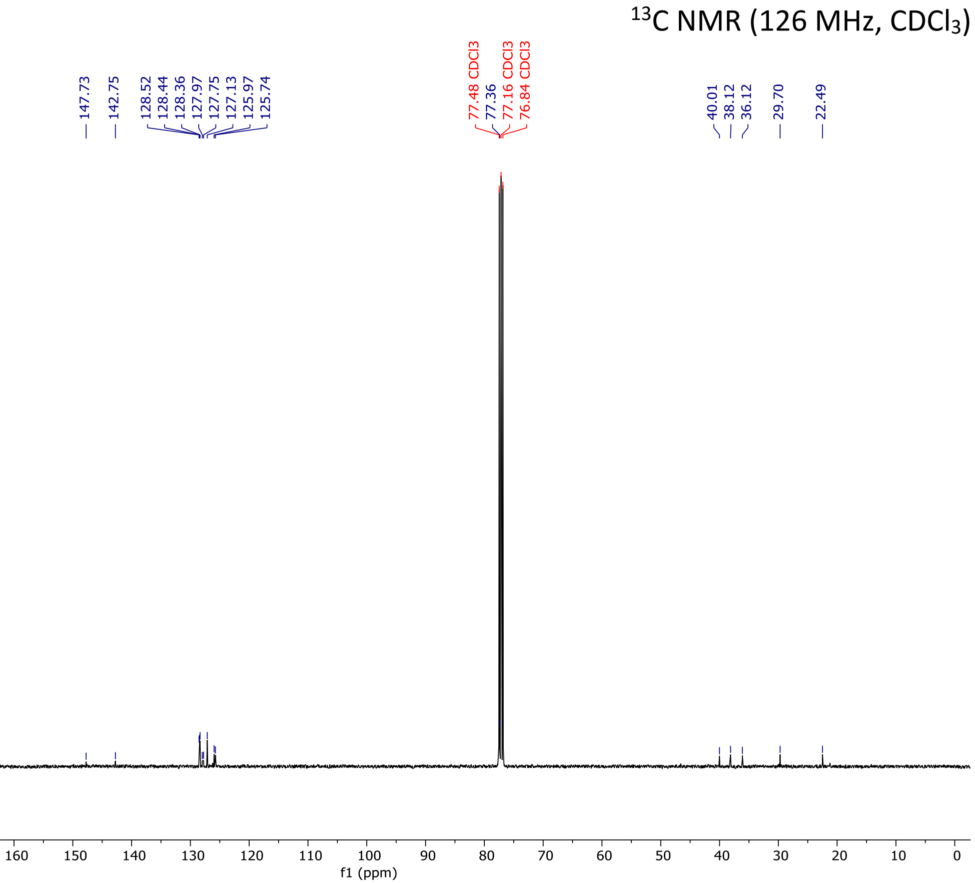

^{13}C NMR (126 MHz, CDCl$_3$)
147.73
142.75
128.52
128.44
128.36
127.97
127.75
127.13
125.97
125.74
77.48 CDCl3
77.36
77.16 CDCl3
76.84 CDCl3
40.01
38.12
36.12
29.70
22.49
200
190
180
170
160
150
140
130
120
110
100
90
80
70
60
50
40
30
20
10
0
f1 (ppm)

S4.6 Borylation ^{1}H-, ^{13}C, and ^{11}B-NMR spectrum

S4.7 5b

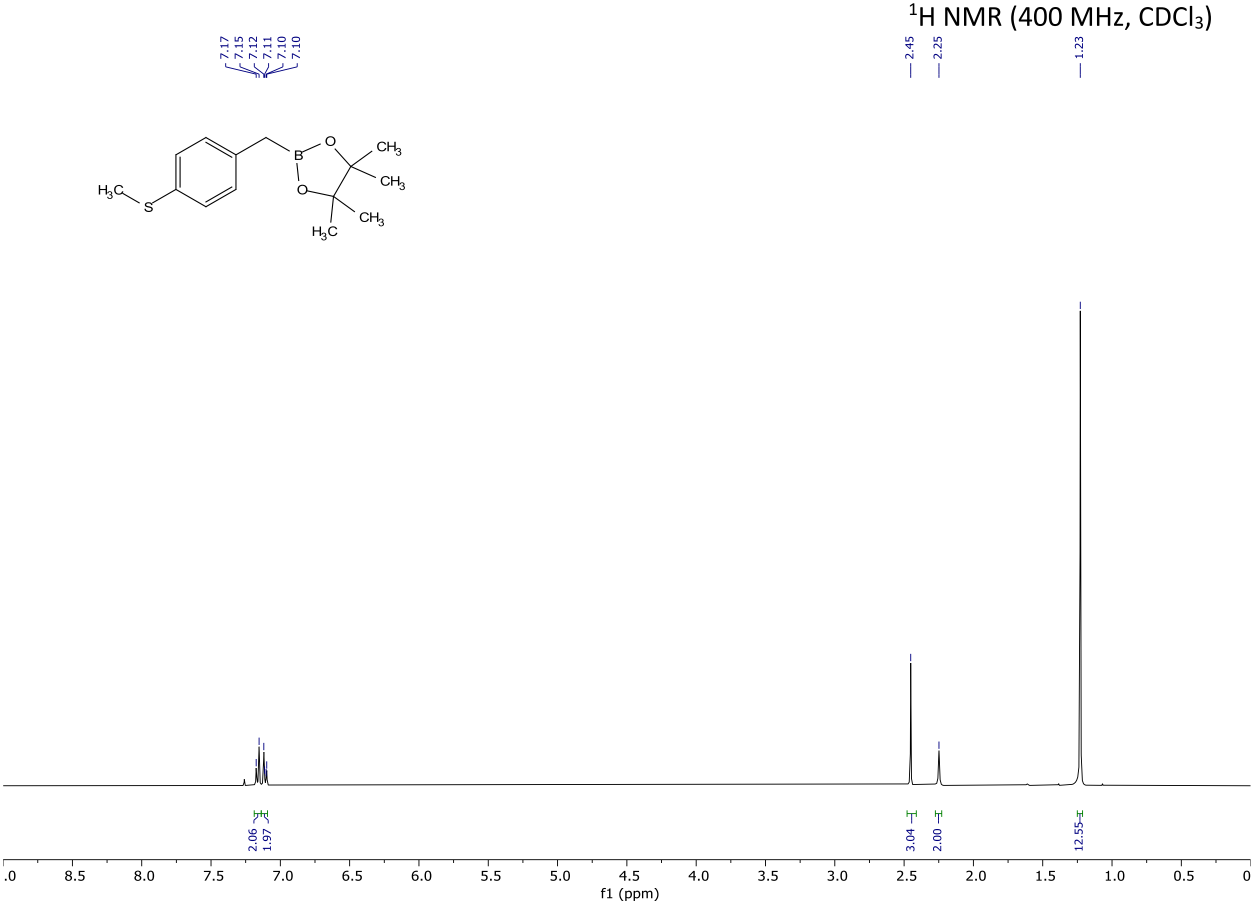

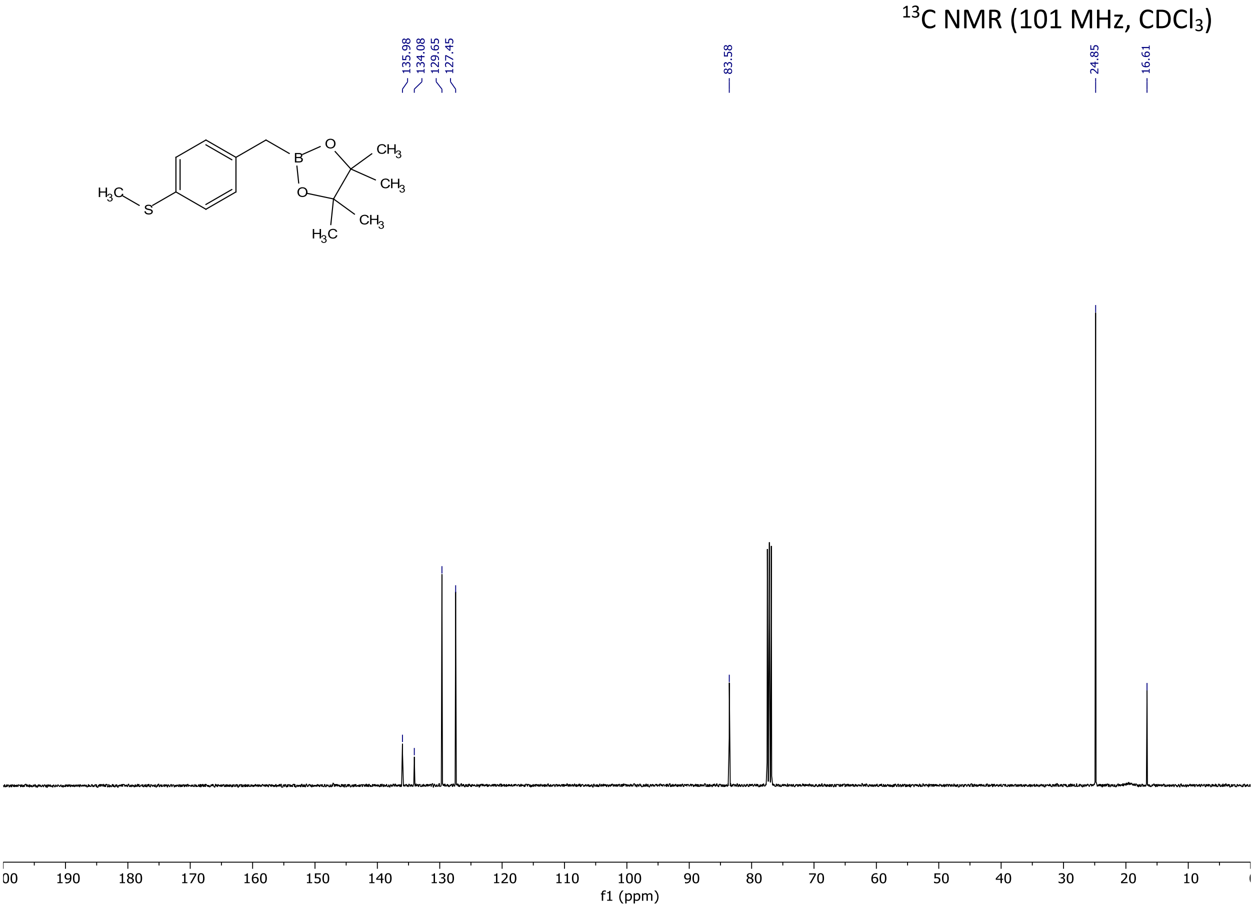

^{13}C NMR (101 MHz, CDCl$_3$)
135.98
134.08
129.65
127.45
83.58
24.85
16.61
H3C
S
B
O
CH3
f1 (ppm)

^{11}B NMR (128 MHz, $CDCl_3$)

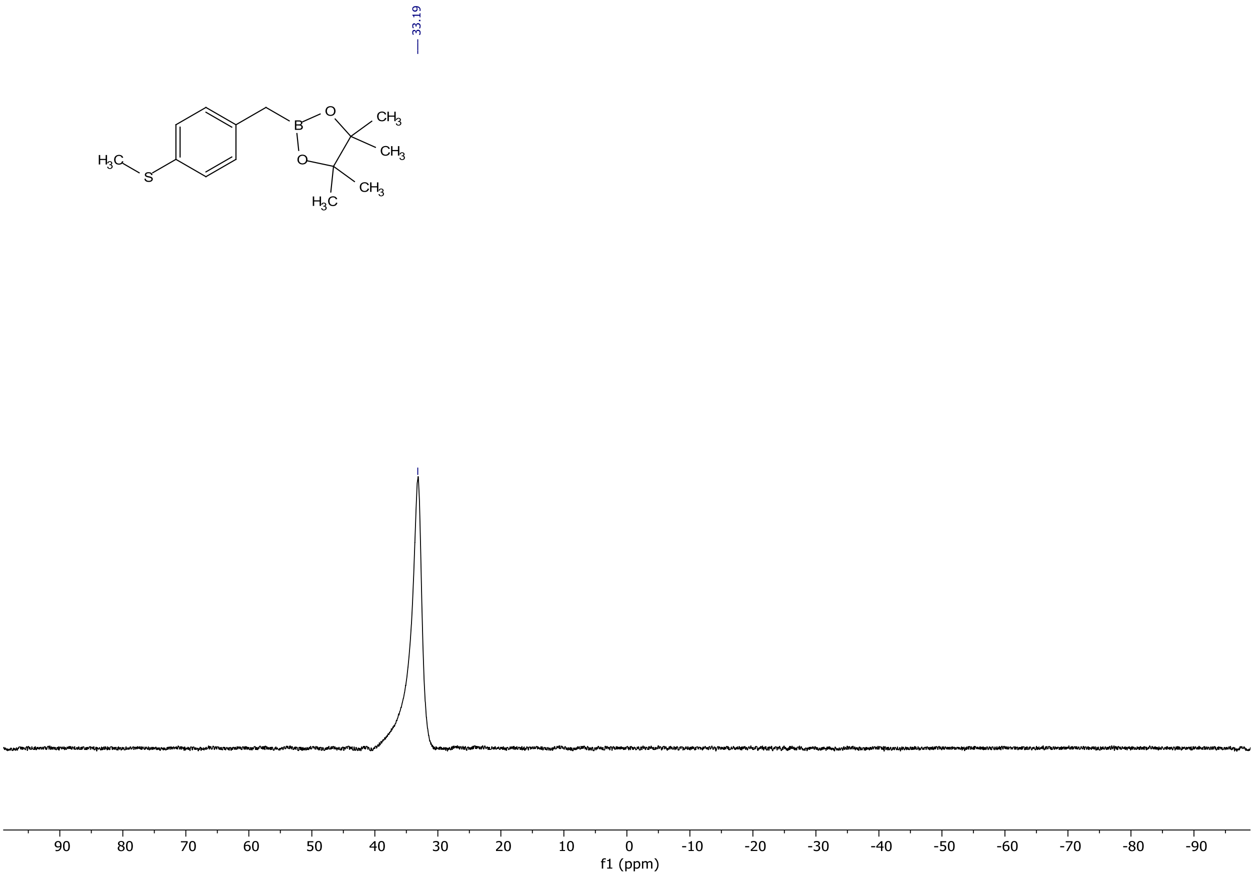

S4.8 5c

^{1}H NMR (400 MHz, $CDCl_3$)

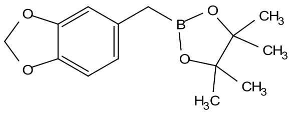

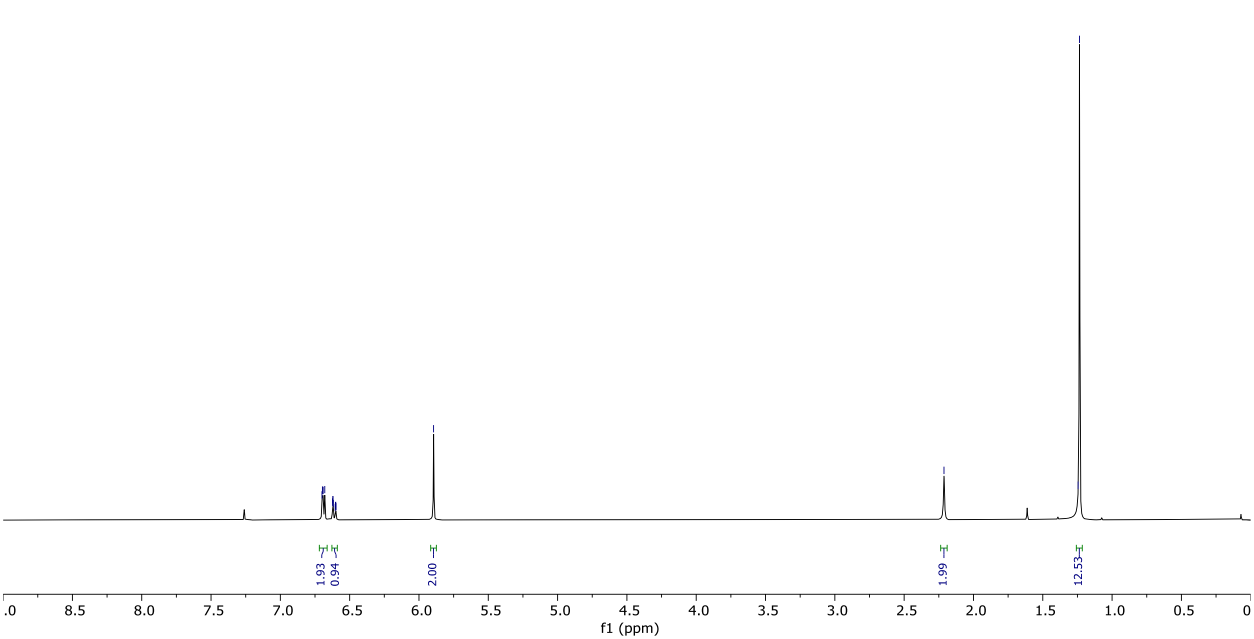

^{13}C NMR (101 MHz, $CDCl_3$)

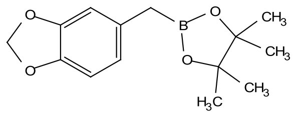

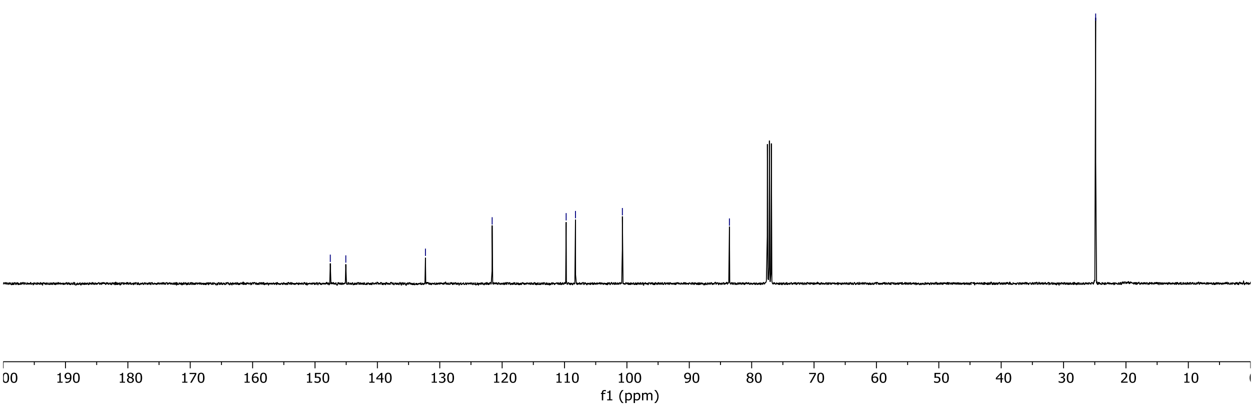

^{11}B NMR (128 MHz, $CDCl_3$)

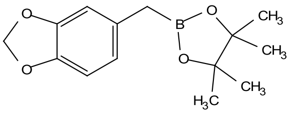

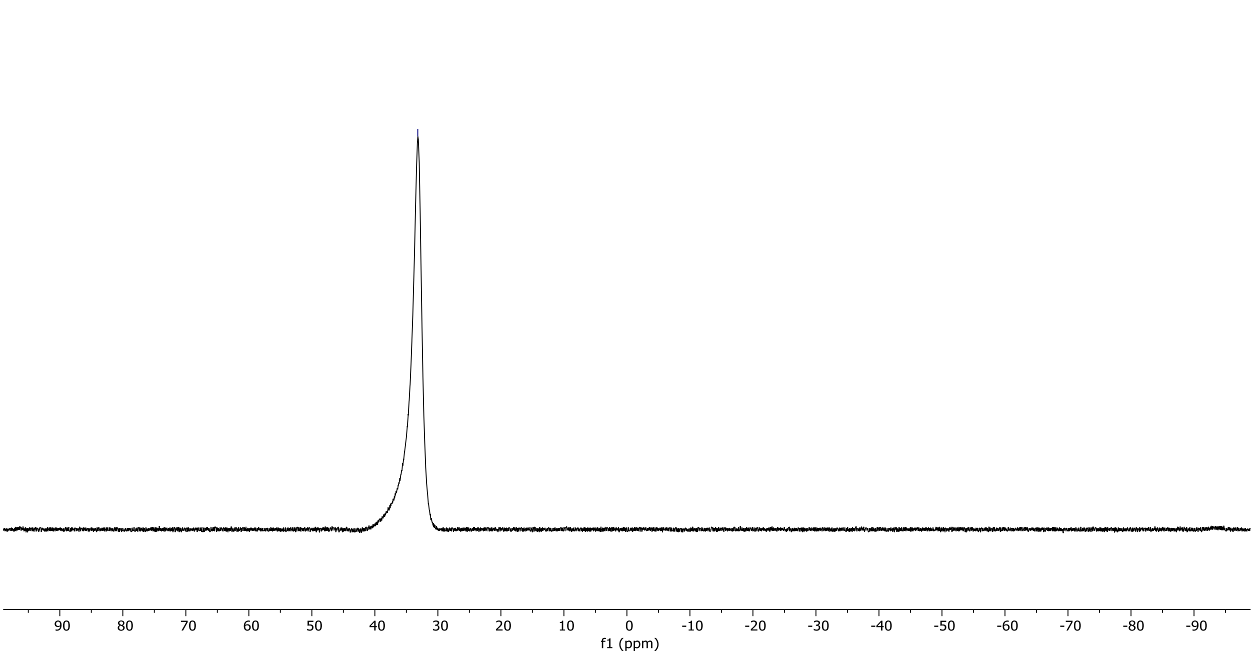

S4.9 5d

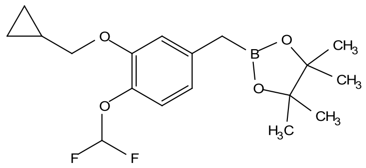

O
O
F
F
B
O
O
CH_3
CH_3
CH_3
H_3C

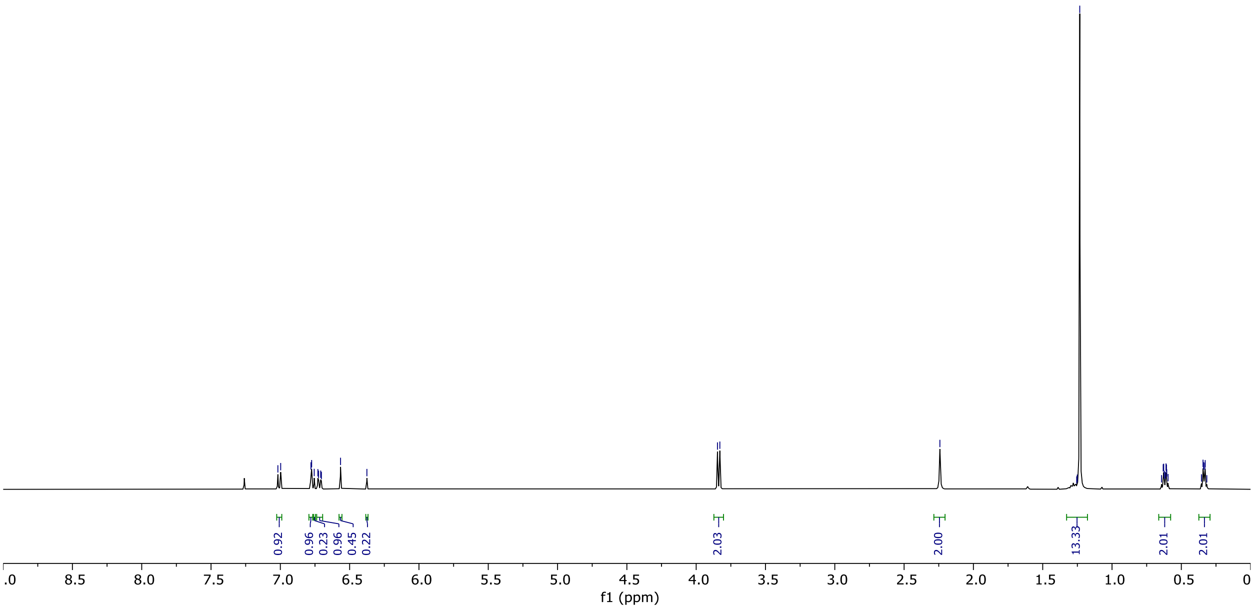

^{1}H NMR (400 MHz, $CDCl_3$)
7.02
7.00
6.78
6.77
6.76
6.73
6.72
6.71
6.70
6.57
6.38
3.85
3.83
2.24
1.25
1.25
1.23
0.64
0.63
0.63
0.62
0.62
0.61
0.61
0.61
0.61
0.60
0.34
0.34
0.33
0.92
0.96
0.23
0.96
0.45
0.22
2.03
2.00
13.33
2.01
2.01
8.5
8.0
7.5
7.0
6.5
6.0
5.5
5.0
4.5
4.0
3.5
3.0
2.5
2.0
1.5
1.0
0.5
0
f1 (ppm)

^{13}C NMR (101 MHz, $CDCl_3$)

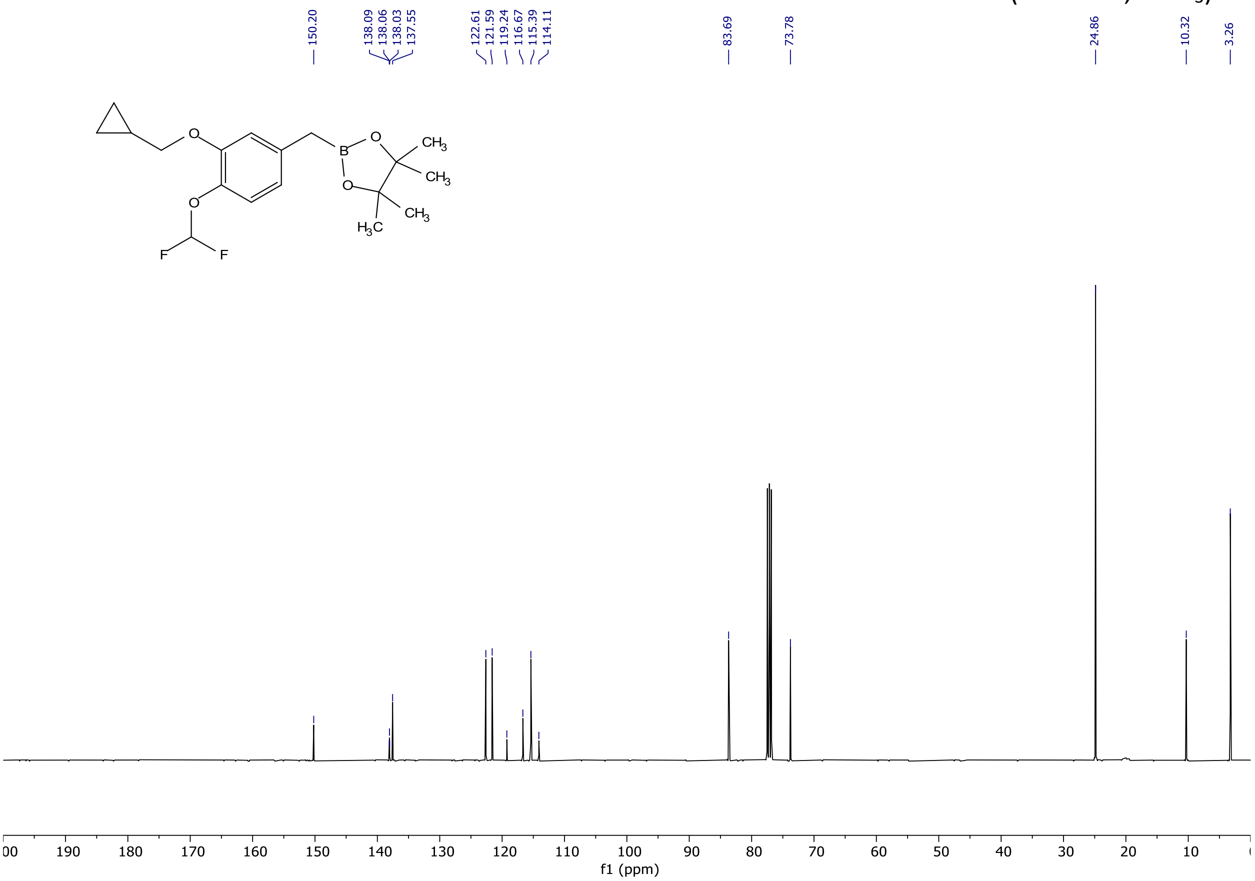

^{11}B NMR (128 MHz, $CDCl_3$)

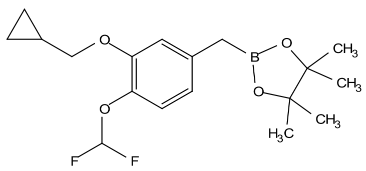

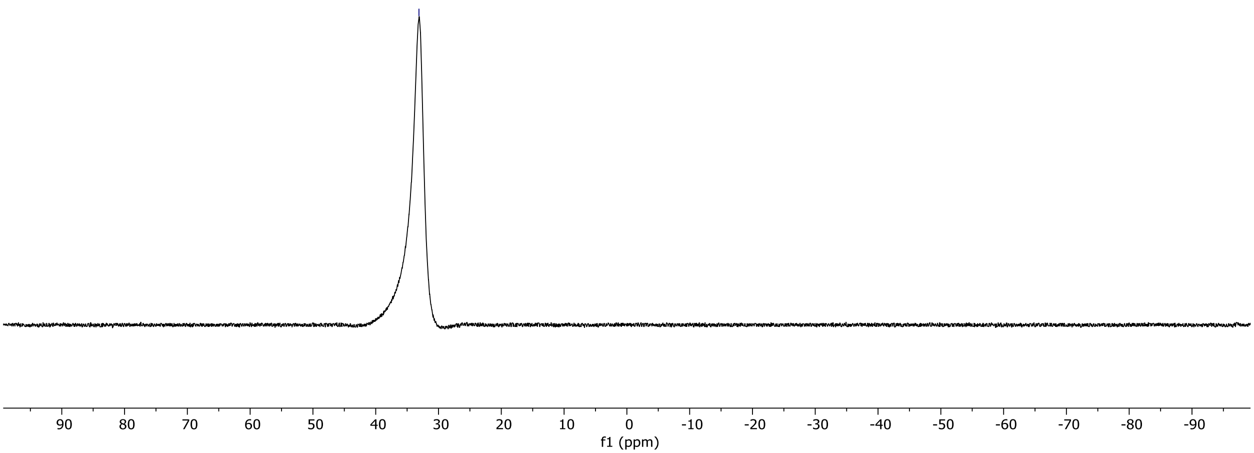

^{19}F NMR (377 MHz, $CDCl_3$)

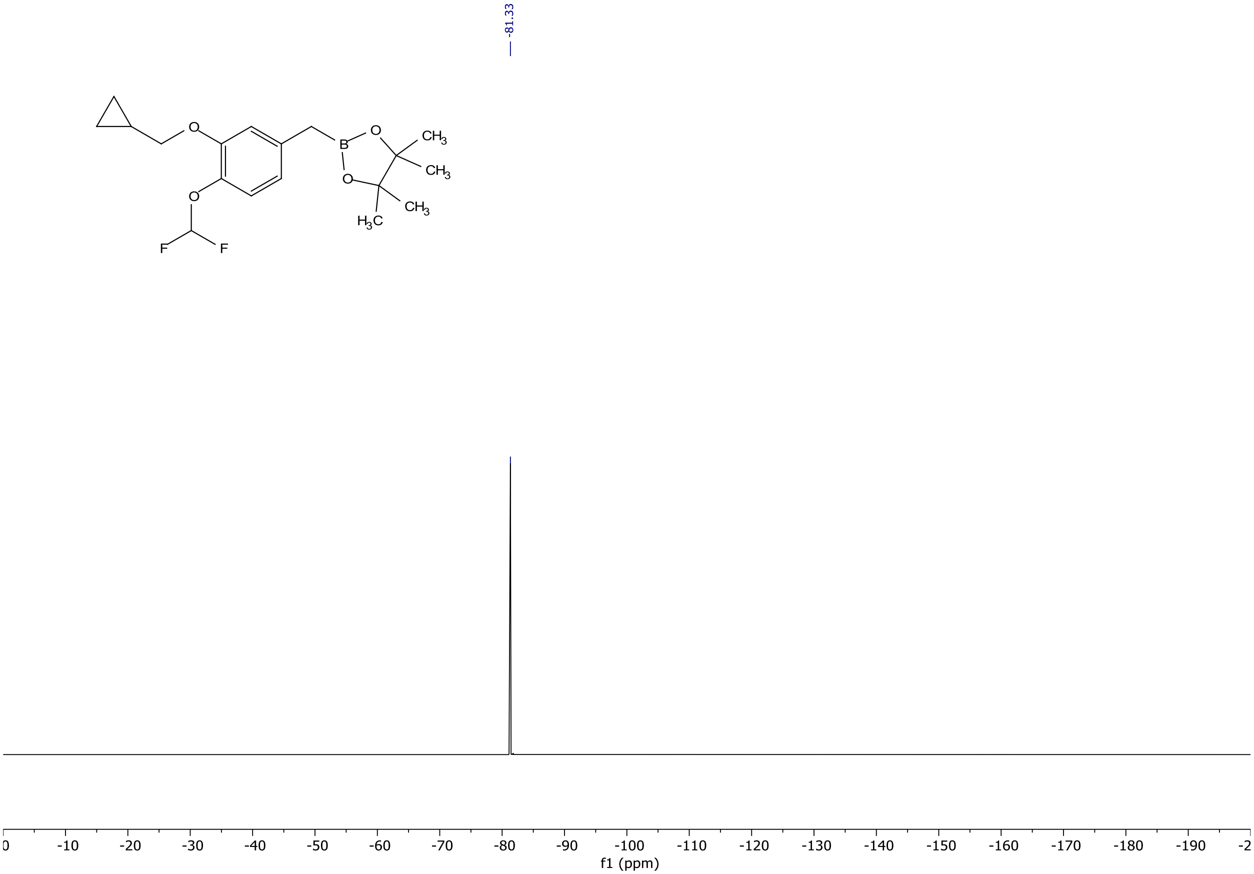

S4.10 5e

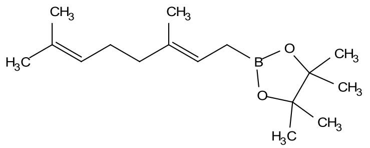
CH3
CH3
H3C
B
O
O
CH3
CH3
CH3
H3C

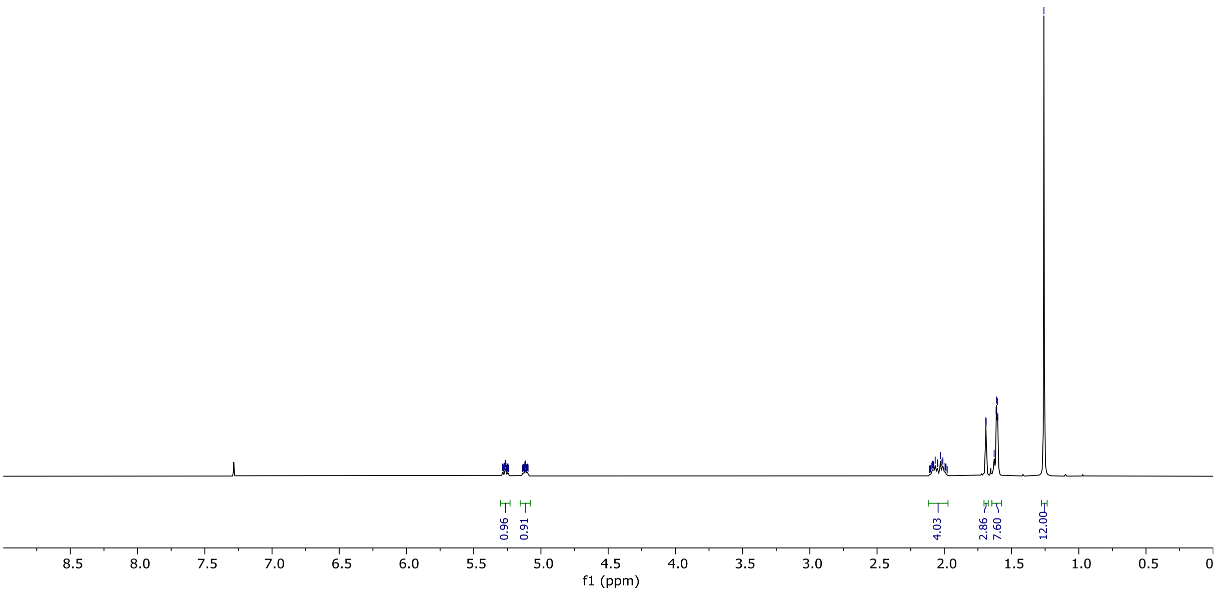
1H NMR (400 MHz, CDCl3)
5.29
5.28
5.28
5.27
5.27
5.26
5.26
5.25
5.25
5.24
5.24
5.14
5.13
5.13
5.12
5.12
5.12
5.11
5.11
5.11
5.10
5.10
2.11
2.11
2.11
2.10
2.09
2.09
2.09
2.08
2.08
2.08
2.07
2.05
2.05
2.03
2.02
2.01
1.99
1.99
1.99
1.98
1.69
1.69
1.63
1.61
1.61
1.61
1.60
1.26
0.96
0.91
4.03
2.86
7.60
12.00
8.5
8.0
7.5
7.0
6.5
6.0
5.5
5.0
4.5
4.0
3.5
3.0
2.5
2.0
1.5
1.0
0.5
0
f1 (ppm)

^{13}C NMR (101 MHz, $CDCl_3$)

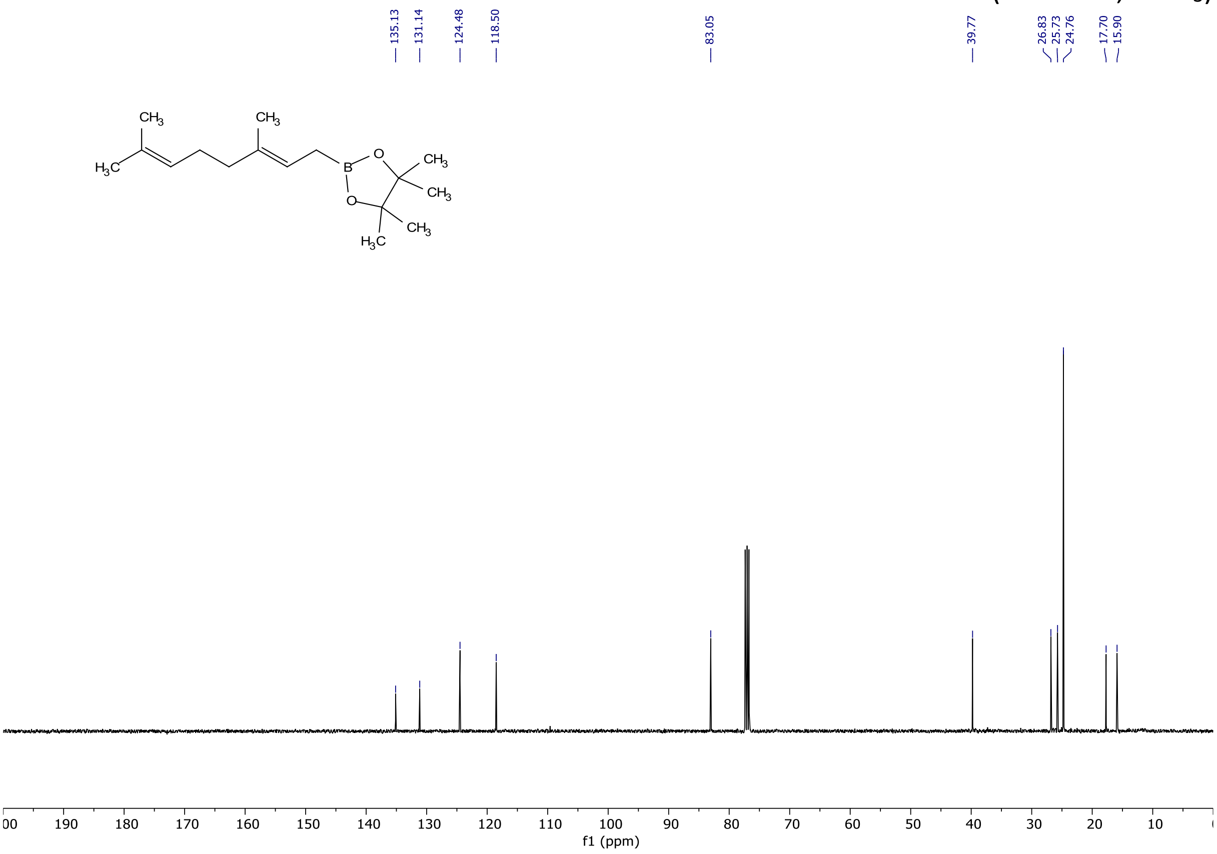

^{11}B NMR (128 MHz, $CDCl_3$)

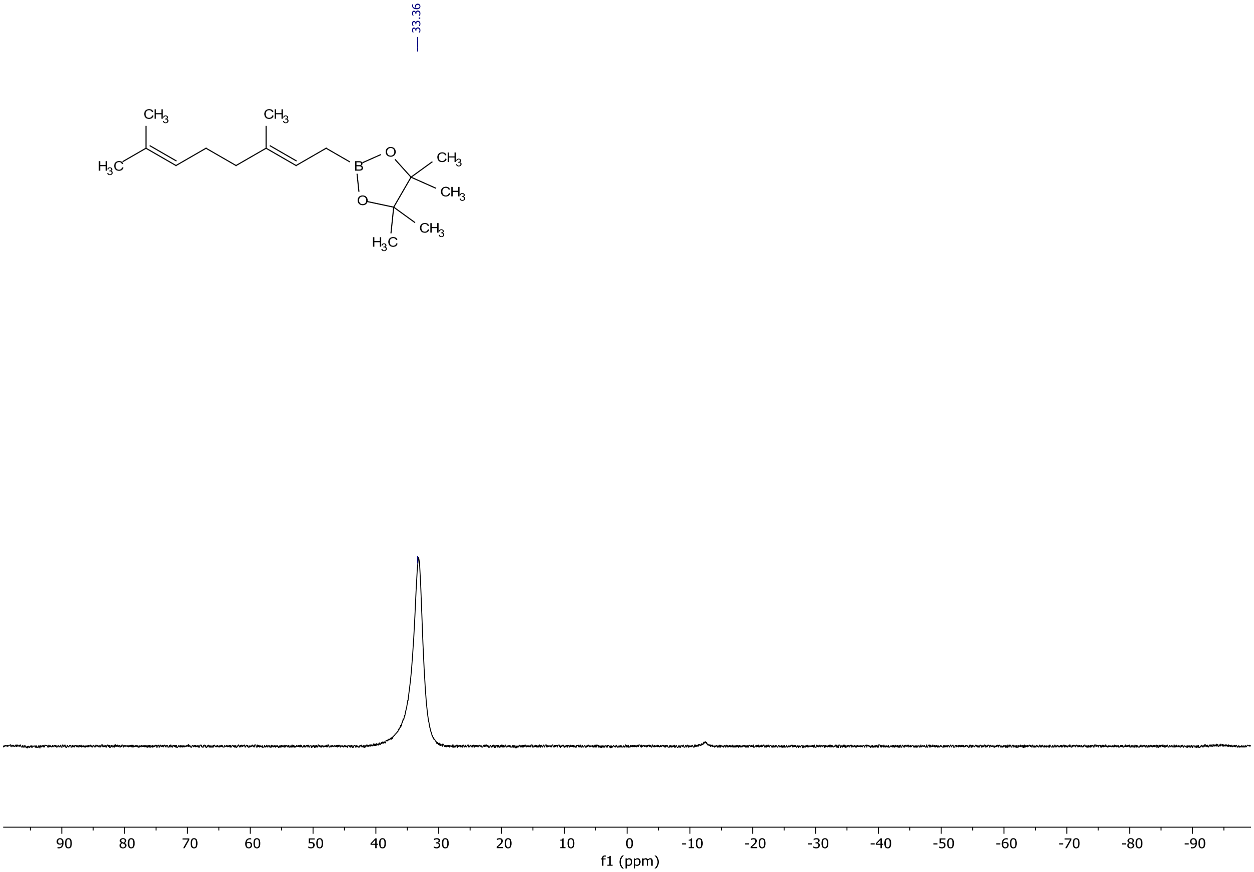

S4.11 5f

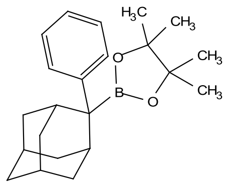

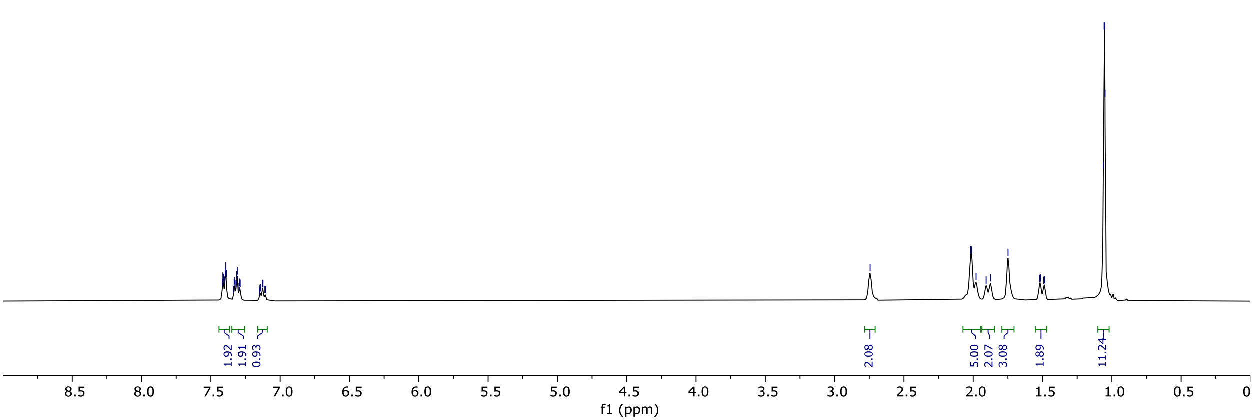
^{1}H NMR (400 MHz, CDCl$_3$)
7.42 7.41 7.41 7.40 7.40 7.39 7.39 7.34 7.33 7.33 7.32 7.31 7.31 7.30 7.29 7.29 7.15 7.15 7.14 7.13 7.12 7.11 7.11
2.74
2.02 2.01 1.98 1.91 1.88 1.75 1.52 1.52 1.49 1.49 1.06 1.06 1.05 1.05
1.92 1.91 0.93 2.08 5.00 2.07 3.08 1.89 11.24
8.5 8.0 7.5 7.0 6.5 6.0 5.5 5.0 4.5 4.0 3.5 3.0 2.5 2.0 1.5 1.0 0.5 0
f1 (ppm)

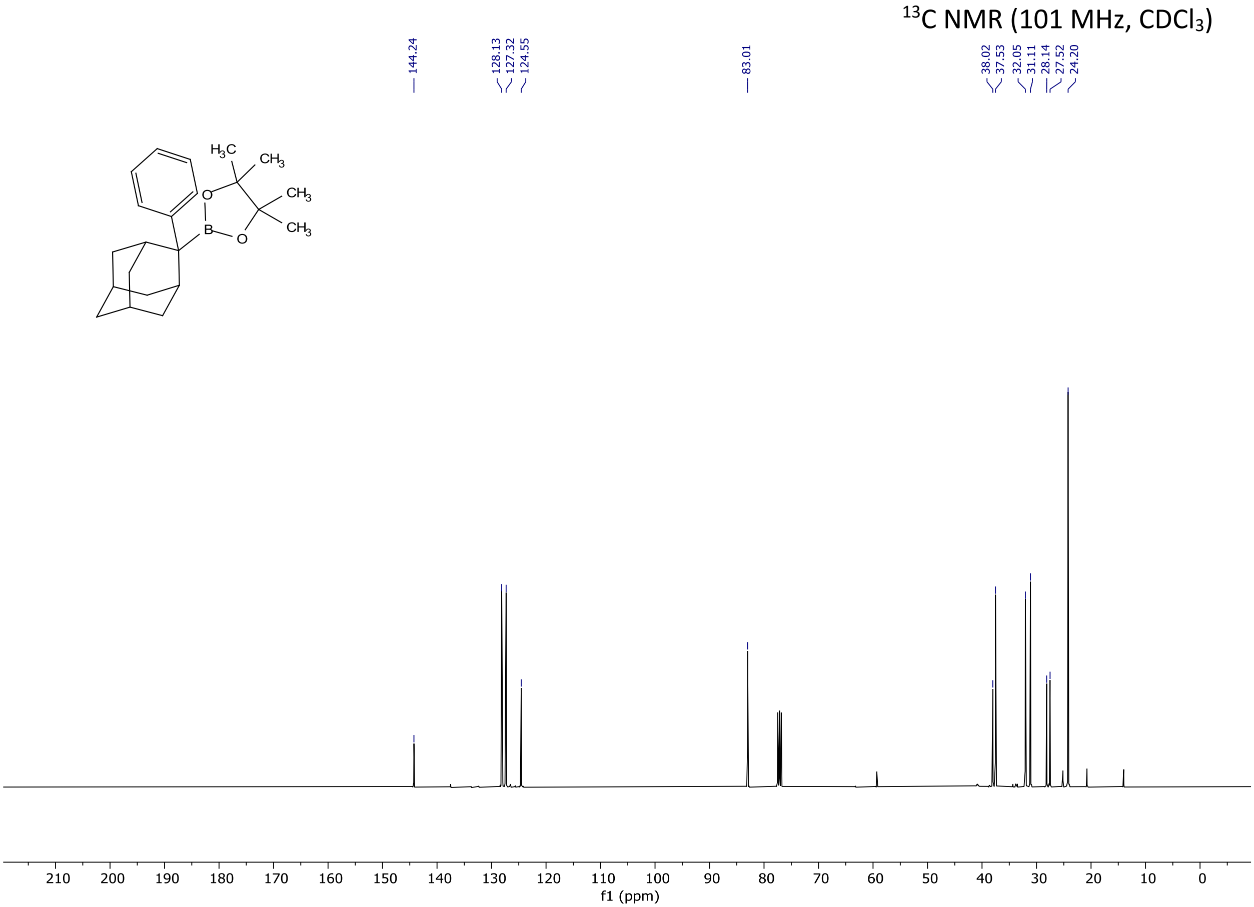
13C NMR (101 MHz, CDCl3)
144.24
128.13
127.32
124.55
83.01
38.02
37.53
32.05
31.11
28.14
27.52
24.20
H3C
CH3
CH3
CH3
O
B
O
210
200
190
180
170
160
150
140
130
120
110
100
90
80
70
60
50
40
30
20
10
0
f1 (ppm)

^{11}B NMR (128 MHz, CDCl3)

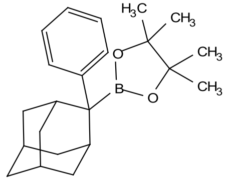

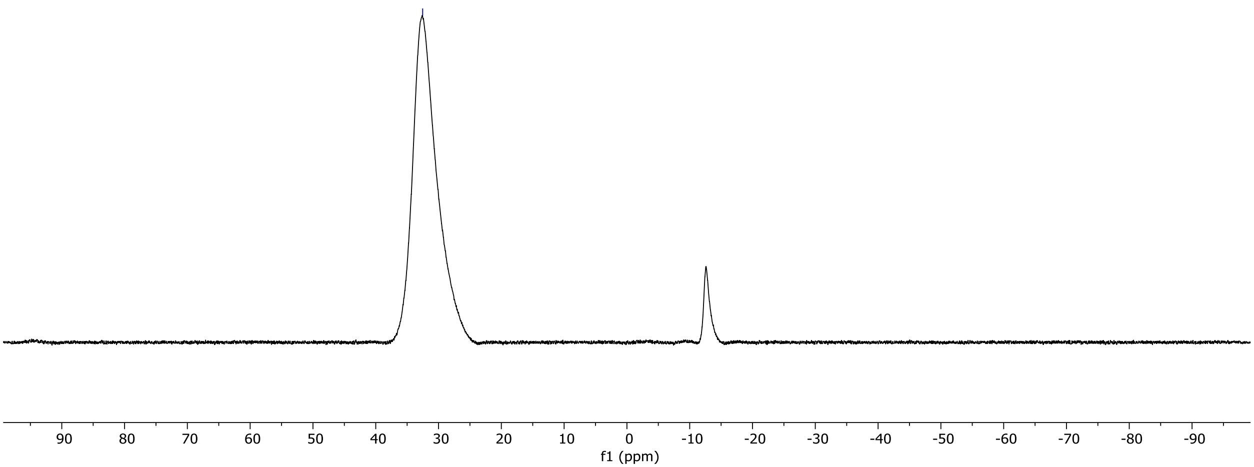

Supplement: Supplementary file 1 — Supporting Information [file ANIE-64-e202501653-s001.pdf]
